# Medication Non‐Adherence in First Episode Psychosis: A Systematic Review

**DOI:** 10.1111/eip.70234

**Published:** 2026-07-30

**Authors:** James Hunt, Anna Georgiades

**Affiliations:** ^1^ Department of Psychosis Studies Institute of Psychiatry, Psychology, and Neuroscience (IoPPN), King's College London London UK; ^2^ Brent Early Intervention Service CNWL, NHS Foundation Trust London UK

**Keywords:** CBT strategies, medication adherence, medication non‐adherence, psychosis, schizophrenia

## Abstract

**Objective:**

Despite the efficacy of antipsychotics, medication non‐adherence remains common among individuals with a First Episode of Psychosis (FEP).

**Methods:**

A systematic review was conducted in accordance with the PRISMA 2020 guidelines to summarise the existing evidence base regarding the reasons, attitudes, and beliefs underlying both medication non‐adherence and adherence in FEP, as well as psychosocial interventions designed to promote medication adherence in psychosis.

**Results:**

Seventy‐six studies were eligible for inclusion. Nineteen reasons for medication non‐adherence in FEP were identified, which were synthesised into 10 overarching themes: Insight and Perceived Need, Autonomy and Control, Trust and the Therapeutic Relationship, Cultural and Spiritual Factors, Side Effects and Perceived Inefficacy, Social and Family Influences, Stigma, Practical Barriers, Substance Use and Symptom‐Driven Factors. Facilitators of adherence included illness insight, motivation to recover and fear of relapse, trust in clinicians, acceptance of the bio‐psychosocial model, experiencing positive treatment outcomes, use of digital reminders and social support. A range of psychosocial interventions demonstrated effectiveness for enhancing medication adherence in psychosis. This included Cognitive Behaviour Therapy, psychoeducation, motivational interviewing, adherence therapy, assertive community treatment, and adherence‐focused psychosocial skills training, particularly when delivered in combination.

**Conclusion:**

Medication non‐adherence in FEP is shaped by a complex interplay of personal, relational, cognitive, cultural and systemic influences. Addressing these factors in the design and delivery of psychosocial interventions may serve to improve medication adherence outcomes in early psychosis. To support clinical application, this review also provides a fictitious clinician–client transcript along with Socratic questions and CBT strategies to assist clinicians in assessing and promoting medication adherence in FEP.

## 1 Introduction

Medication non‐adherence refers to the extent to which a patient's medication‐taking behaviour, such as timing, dosage, or frequency, does not align with clinical recommendations. Medication non‐adherence represents a major challenge in the management of First Episode Psychosis (FEP). Medication adherence in FEP is a critical determinant of clinical outcomes, yet rates remain suboptimal. Clinical guidelines recommend that individuals with FEP are offered oral antipsychotics, typically continued for the first 1–2 years after psychosis onset to reduce the risk of relapse, alongside Cognitive Behavioural Therapy for psychosis (CBTp) (NICE 2014). However, evidence indicates that only 41% adhere consistently, while 39% are non‐adherent, and 20% are inadequately adherent (Coldham et al. 2002). Discontinuation is particularly high early in treatment, with up to 58% of patients stopping medication within the first month and up to 90% within the first year (Mullins et al. 2008; Tiihonen et al. 2011). Deleterious consequences of medication non‐adherence include psychosis symptom exacerbation, increased risk of relapse and suicide, reduced treatment efficacy and ongoing residual symptomatology (Coldham et al. 2002; Hawton et al. 2005; El Abdellati et al. 2020; Lambert, Conus, et al. 2010; Hill et al. 2010). Notably, the risk of suicide has been reported to be 3.75 times higher in non‐adherent patients compared with those who take their medication as prescribed (Hawton et al. 2005). Medication non‐adherence has also been associated with reduced insight, impaired social and occupational functioning, increased substance misuse and reduced quality of life (Robinson 2011; Higashi et al. 2013).

Recent meta‐analytic evidence has found that discontinuation of antipsychotic medication for 2 months or more in FEP is associated with a significantly increased risk of relapse compared with continued treatment, with risk ratios indicating that maintenance treatment approximately halved the relapse rate at 12 months (RR≈0.47) (Kishi et al. 2019). Pooled analyses further estimate that FEP patients who discontinue antipsychotic medication experience relapse rates of around 53%, compared with 19% among those who maintain adherence, highlighting the substantial clinical impact of medication adherence on relapse outcomes in FEP (Thompson et al. 2018). Compared to individuals with chronic psychosis, those with FEP demonstrate greater responsiveness to antipsychotic medication, emphasising the need to promote adherence early in the course of the illness (Ohlsen et al. 2004). Attitudes towards medication formed after a first episode of psychosis predict long‐term adherence patterns, underscoring the importance of addressing barriers early in the treatment trajectory (Birchwood et al. 1998; Harrison et al. 2001; Dai et al. 2023).

A comprehensive review found that factors contributing to medication non‐adherence in FEP include patient‐related factors (e.g., substance use), environmental factors (e.g., limited family involvement), medication‐related factors (e.g., rapid remission of negative symptoms), and illness‐related determinants (e.g., more positive symptoms) (Leclerc et al. 2015). This review also highlighted the importance of therapeutic alliance and voluntary admission in supporting adherence (Leclerc et al. 2015). While previous reviews have examined non‐adherence factors or interventions separately, few have systematically integrated patient‐reported reasons, attitudes, and beliefs alongside evidence on psychosocial interventions targeting adherence. Identifying these factors would enable the development of personalised formulations and targeted interventions within CBTp, which would improve client outcomes. Psychosocial interventions can target attitudinal, cognitive, and behavioural barriers—such as stigma, mistrust, and negative perceptions—that medication alone cannot address, making them essential for holistic management. This review therefore seeks to address this gap by identifying barriers, facilitators, and strategies to support medication adherence, thereby promoting recovery in individuals with FEP within early intervention services.

### 1.1 Aims

The aim of this systematic review was to synthesise the existing evidence regarding the reasons, attitudes, and beliefs underlying both medication non‐adherence and adherence in FEP, as well as to identify psychosocial interventions designed to promote medication adherence in psychosis.

## 2 Methods

The review adhered to the Preferred Reporting Items for Systematic Reviews and Meta‐Analyses (PRISMA 2020) reporting guidelines (Page et al. 2021). Methodological procedures and eligibility criteria were defined a priori and detailed in a protocol registered with the PROSPERO International Prospective Register of Systematic Reviews (CRD420251066438). A completed PRISMA 2020 checklist is provided in the Supporting Information.

### 2.1 Search Strategy

A systematic database search of MEDLINE (including PubMed), EMBASE, Global Health and APA PsycINFO was conducted using an OVID search tool from database inception to 30th June 2025. The following search strings were used: Psychosis OR Psychotic OR Schizophreni* AND Medication adheren* OR Medication nonadheren* OR Medication non‐adheren* OR Medication complian* OR Medication noncomplian* OR Medication non‐complian* AND Attitude* OR Perspective* OR Reason* OR Belief* OR Experience* OR View* OR Trial* OR Intervention* OR CBT OR Cognitive behavio* therap* OR Therap*. These databases were selected to ensure comprehensive coverage of biomedical, psychological, and global mental health literature relevant to early psychosis and medication adherence.

### 2.2 Study Selection

All study designs, publication dates, and follow‐up durations were considered eligible. This inclusive approach was intended to capture the full breadth of evidence in a field characterised by heterogeneous methodologies, encompassing both quantitative and qualitative research. No date restrictions were applied to allow for identification of temporal trends and to maximise search sensitivity.

Studies were included if they:

1. Explored the reasons, attitudes, or beliefs regarding medication non‐adherence in early psychosis; and/or
2. Explored the reasons, attitudes, or beliefs regarding medication adherence in early psychosis; and/or
3. Evaluated psychosocial interventions aimed at promoting medication adherence in individuals with psychosis.

**FIGURE 1:**
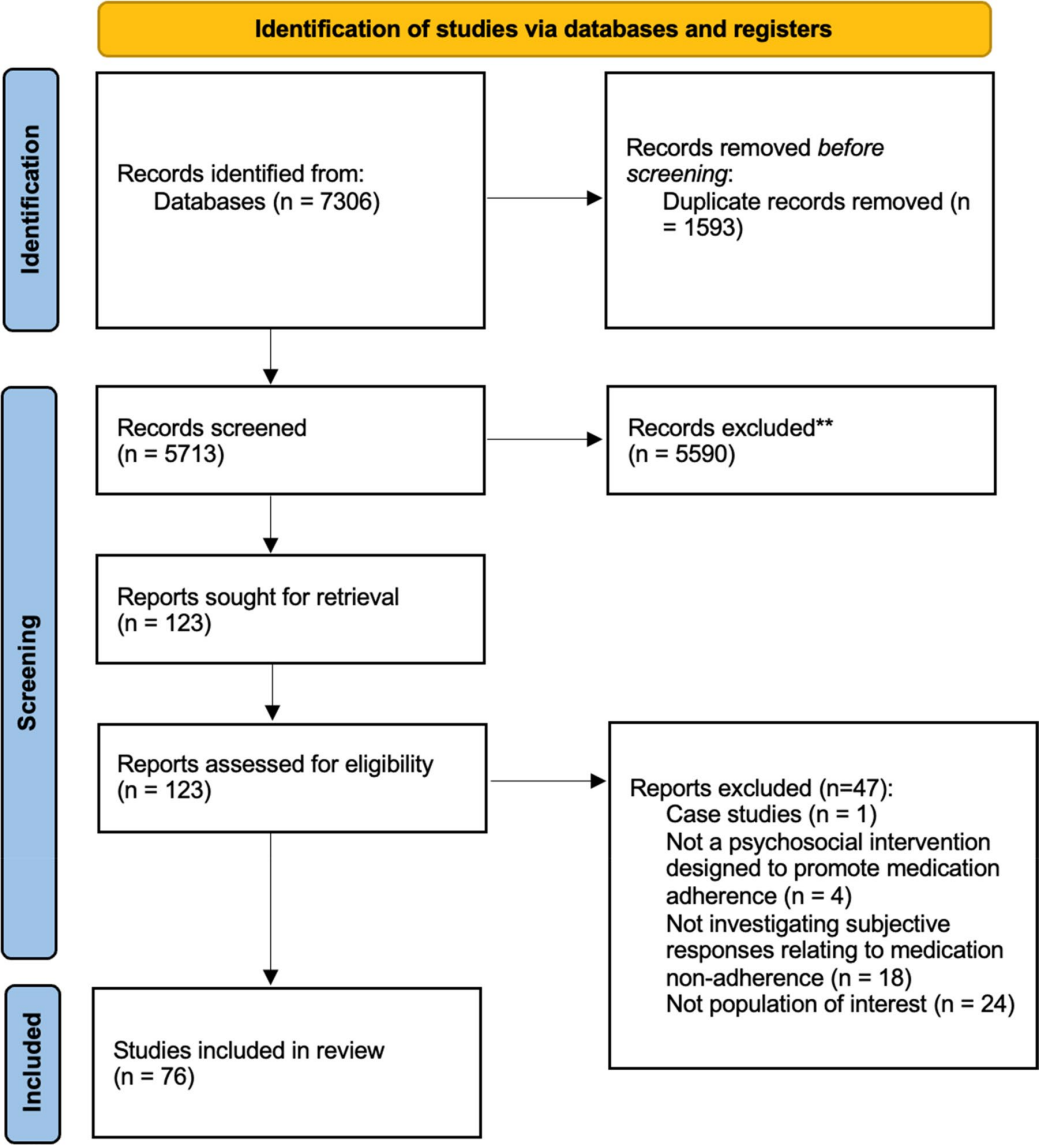
PRISMA 2020 flow diagram illustrating study selection for medication adherence and non‐adherence themes in FEP, as well as psychosocial interventions promoting adherence.

Eligibility was limited to studies published in peer‐reviewed journals and in English to ensure a minimum standard of methodological rigour and reporting. Participants were required to have a diagnosed primary psychotic disorder, specifically schizophrenia or First Episode Psychosis, using a reliable and validated clinical diagnostic tool. Examples include DSM‐5‐TR or ICD‐11 (APA 2022; World Health Organization 2019), but studies using DSM‐IV or other validated instruments were also eligible (American Psychiatric Association 2013). This approach followed the registered PROSPERO protocol and accommodated changes in diagnostic frameworks over time, while ensuring methodological rigour and comparability across studies. The Prisma flow diagram details the study selection process (Figure 1).

### 2.3 Exclusion Criteria

Studies not published in English, unpublished or grey literature, conference abstracts, book chapters, meta‐analyses, reviews of any type, and case reports were excluded. Reviews were excluded because this review focused on primary empirical studies to ensure synthesis of original evidence rather than summarising secondary literature. Studies involving drug‐induced or organic psychosis, or bipolar disorder with psychotic features, were omitted to maintain clinical and etiological homogeneity. These conditions differ substantially in prognosis, treatment pathways, and the psychosocial and cognitive mechanisms underlying medication adherence. These decisions were made a priori to enhance the interpretability and applicability of our findings to the early psychosis population.

### 2.4 Data Extraction Process

All relevant studies were uploaded into Microsoft Excel, where a dedicated worksheet was created to systematically document each study's characteristics. Data extracted included: author (name and year), country, study design, sample size and setting, mean age (±SD), diagnostic criteria or assessment tools, measures of medication adherence, reported reasons, attitudes, or beliefs regarding medication non‐adherence, intervention type and duration (for psychosocial intervention studies), follow‐up duration, and key quantitative and qualitative findings. Clinical implications relevant to medication adherence were also captured. Data extraction was performed by one reviewer (J.H.) and subsequently verified by a second reviewer (A.G.) to ensure accuracy and consistency. Discrepancies were resolved through discussion.

### 2.5 Quality Appraisal

Methodological quality of quantitative studies (see Tables 1 and 2) was evaluated using the Effective Public Health Practice Project (EPHPP) tool (Thomas 2003), while qualitative studies (see Table 3) were appraised using the Joanna Briggs Institute (JBI) Critical Appraisal Checklist for Qualitative Research (Lockwood et al. 2015).

**TABLE 1:** Quality assessment for quantitative studies of medication non‐adherence—EPHPP tool.

Author (year)	Selection bias	Study design	Confounders	Data collection methods	Withdrawals and dropouts	Analyses (appropriateness)	Global rating
Baloush‐Kleinman et al. (2011)	Weak	Moderate	Moderate	Strong	Strong	Yes	Moderate
Choi and Kweon (2023)	Strong	Moderate	Moderate	Strong	Moderate	Yes	Strong
Elowe et al. (2022)	Strong	Moderate	Moderate	Moderate	Weak	Yes	Moderate
Endale Gurmu et al. (2014)	Moderate	Weak	Moderate	Weak	Moderate	Yes	Weak
Hickling et al. (2018)	Strong	Moderate	Weak	Strong	Moderate	Yes	Moderate
Hui et al. (2006)	Strong	Weak	Moderate	Moderate	Moderate	Yes	Moderate
Kolliakou et al. (2015)	Moderate	Weak	Moderate	Moderate	Moderate	Yes	Moderate
Mané et al. (2015)	Strong	Moderate	Moderate	Moderate	Moderate	Yes	Strong
Miller and McCormack (2006)	Weak	Moderate	Weak	Moderate	Moderate	Yes	Weak
Mutsatsa et al. (2003)	Moderate	Moderate	Moderate	Weak	Moderate	Yes	Moderate
Ocansey et al. (2025)	Moderate	Moderate	Moderate	Strong	Moderate	Yes	Strong
Perkins et al. (2006)	Moderate	Strong	Strong	Moderate	Weak	Yes	Moderate
Polillo et al. (2022)	Weak	Moderate	Weak	Moderate	Strong	Yes	Weak
Quach et al. (2009)	Strong	Strong	Strong	Moderate	Moderate	Yes	Strong
Sapra et al. (2014)	Moderate	Moderate	Weak	Strong	Moderate	Yes	Moderate
So et al. (2024)	Weak	Moderate	Weak	Strong	Moderate	Yes	Weak
Stürup et al. (2023)	Weak	Moderate	Strong	Strong	Moderate	Yes	Moderate
Tan et al. (2019)	Moderate	Moderate	Moderate	Moderate	Moderate	Yes	Moderate

Abbreviation: EPHPP, Effective Public Health Practice Project (Thomas 2003).

**TABLE 2:** Quality assessment for psychosocial interventions to promote medication adherence in psychosis–EPHPP tool.

Author (year)	Selection bias	Study design	Confounders	Data collection methods	Withdrawals and dropouts	Analyses (appropriateness)	Global rating
Alinaitwe et al. (2024)	Moderate	Strong	Strong	Strong	Strong	Yes	Strong
Anderson et al. (2010)	Weak	Strong	Moderate	Strong	Strong	Yes	Moderate
Barkhof et al. (2013)	Moderate	Strong	Moderate	Strong	Strong	Yes	Moderate
Bhawana et al. (2022)	Weak	Moderate	Weak	Strong	Weak	Yes	Weak
Brown et al. (2013)	Strong	Moderate	Weak	Weak	Strong	Yes	Weak
Budiono et al. (2021)	Weak	Strong	Moderate	Strong	Strong	Yes	Moderate
Byerly et al. (2005)	Moderate	Moderate	Moderate	Strong	Moderate	Yes	Strong
Can and Budak (2025)	Strong	Moderate	Moderate	Strong	Strong	Yes	Strong
Chien et al. (2024)	Strong	Strong	Strong	Strong	Strong	Yes	Strong
Chien et al. (2015)	Moderate	Strong	Strong	Strong	Strong	Yes	Strong
Chien et al. (2016)	Strong	Strong	Strong	Strong	Strong	Yes	Strong
Dahan et al. (2016)	Moderate	Strong	Strong	Moderate	Strong	Yes	Strong
Dikec and Kutlu (2016)	Weak	Strong	Strong	Moderate	Strong	Yes	Moderate
Gleeson et al. (2013)	Strong	Strong	Strong	Strong	Moderate	Yes	Strong
Gray et al. (2006)	Moderate	Strong	Strong	Strong	Moderate	Yes	Strong
Harmanci and Budak (2022)	Weak	Moderate	Moderate	Moderate	Strong	Yes	Moderate
Kızılırmak Tatu and Demir (2020)	Weak	Moderate	Moderate	Strong	Strong	Yes	Moderate
Kopelowicz et al. (2012)	Moderate	Strong	Strong	Moderate	Moderate	Yes	Strong
Lambert, Bock, et al. (2010); Lambert, Conus, et al. (2010)	Moderate	Moderate	Strong	Strong	Strong	Yes	Strong
Maneesakorn et al. (2007)	Moderate	Strong	Strong	Strong	Strong	Yes	Strong
Marchira et al. (2019)	Moderate	Strong	Weak	Weak	Weak	Yes	Weak
Ngoc et al. (2016)	Moderate	Strong	Strong	Weak	Strong	Yes	Moderate
O'Donnell et al. (2003)	Moderate	Strong	Strong	Moderate	Strong	Yes	Strong
Oneib et al. (2025)	Moderate	Moderate	Moderate	Strong	Weak	Yes	Moderate
Pitschel‐Walz et al. (2006)	Strong	Strong	Strong	Moderate	Moderate	Yes	Strong
Randall et al. (2017)	Moderate	Moderate	Strong	Strong	Moderate	Yes	Strong
Robinson et al. (2018)	Strong	Strong	Moderate	Weak	Weak	Yes	Weak
Schulz et al. (2013)	Moderate	Strong	Strong	Strong	Moderate	Yes	Strong
Skarsholm et al. (2014)	Moderate	Moderate	Strong	Strong	Moderate	Yes	Strong
Staring et al. (2010)	Moderate	Strong	Strong	Strong	Strong	Yes	Strong
Tessier et al. (2023)	Weak	Strong	Moderate	Strong	Strong	Yes	Moderate
Uzenoff et al. (2008)	Weak	Strong	Moderate	Moderate	Moderate	Yes	Moderate
Valencia et al. (2012)	Strong	Strong	Moderate	Weak	Strong	Yes	Moderate
Zhu et al. (2021)	Moderate	Strong	Strong	Strong	Strong	Yes	Strong

Abbreviation: EPHPP, Effective Public Health Practice Project (Thomas 2003).

**TABLE 3:** Quality assessment for qualitative studies of medication non‐adherence—JBI tool.

Author (year)	1. Is there congruity between the stated philosophical perspective and the research methodology?	2. Is there congruity between the research methodology and the research question or objectives?	3. Is there congruity between the research methodology and the methods used to collect data?	4. Is there congruity between the research methodology and the representation and analysis of data?	5. Is there congruity between the research methodology and the interpretation of results?	8. Are participants, and their voices, adequately represented?	9. Is the research ethical according to current criteria or, for recent studies, and is there evidence of ethical approval by an appropriate body?	10. Do the conclusions drawn in the research report flow from the analysis, or interpretation, of the data?	Include if yes to 2–5, 8–10
Archie et al. (2013)	No	Yes	Yes	Yes	Yes	Yes	Yes	Yes	✓✓
Artaud et al. (2020)	Yes	Yes	Yes	Yes	Yes	Yes	Yes	Yes	✓✓
Baker et al. (2015)	No	Yes	Yes	Yes	Yes	Yes	Yes	Yes	✓✓
Bhikha et al. (2015)	Yes	Yes	Yes	Yes	Yes	Yes	Yes	Yes	✓✓
Bjornestad et al. (2017)	Yes	Yes	Yes	Yes	Yes	Yes	Yes	Yes	✓✓
Brown et al. (2013)	Yes	Yes	Yes	Yes	Yes	Yes	Yes	Yes	✓✓
Chua et al. (2024)	No	Yes	Yes	Yes	Yes	Yes	Yes	Yes	✓✓
Cowan et al. (2020)	Yes	Yes	Yes	Yes	Yes	Yes	Yes	Yes	✓✓
Dijkstra et al. (2024)	Yes	Yes	Yes	Yes	Yes	Yes	Yes	Yes	✓✓
Eisner et al. (2014)	No	Yes	Yes	Yes	Yes	Yes	Yes	Yes	✓✓
Gray and Deane (2016)	No	Yes	Yes	Yes	Yes	Yes	Yes	Yes	✓✓
Hon (2012)	No	Yes	Yes	Yes	Yes	Yes	Yes	Yes	✓✓
Hui et al. (2018)	No	Yes	Yes	Yes	Yes	Yes	Yes	Yes	✓✓
Intharit et al. (2021)	No	Yes	Yes	Yes	Yes	Yes	Yes	Yes	✓✓
Islam et al. (2015)	No	Yes	Yes	Yes	Yes	Yes	Yes	Yes	✓✓
Lobbana et al. (2010)	No	Yes	Yes	Yes	Yes	Yes	Yes	Yes	✓✓
Niemeyer et al. (2018)	No	Yes	Yes	Yes	Yes	Yes	Yes	Yes	✓✓
Penny et al. (2009)	Yes	Yes	Yes	Yes	Yes	Yes	Yes	Yes	✓✓
Perry et al. (2007)	Yes	Yes	Yes	Yes	Yes	Yes	No	Yes	✓
Rathod et al. (2023)	No	Yes	Yes	Yes	Yes	Yes	Yes	Yes	✓✓
Sheridan Rains et al. (2024)	No	Yes	Yes	Yes	Yes	Yes	Yes	Yes	✓✓
Stewart (2013)	No	Yes	Yes	Yes	Yes	Yes	Yes	Yes	✓✓
Vaingankar et al. (2020)	No	Yes	Yes	Yes	Yes	Yes	Yes	Yes	✓✓
van der Heijden‐Hobus et al. (2024)	No	Yes	Yes	Yes	Yes	Yes	Yes	Yes	✓✓
Yeisen et al. (2017)	Yes	Yes	Yes	Yes	Yes	Yes	Yes	Yes	✓✓

*Note:* JBI, Joanna Briggs Institute Quality Assessment Tool (Lockwood, Munn, and Porritt 2015).

## 3 Results

Among the 7306 studies identified, 76 were eligible for inclusion. The characteristics and principal findings of medication adherence and medication non‐adherence studies are summarised in Table 4. For psychosocial interventions aiming to enhance medication adherence in psychosis, findings are summarised in Table 5.

**TABLE 4:** Medication non‐adherence studies in first episode psychosis (*n* = 43).

Author (year)	Country and type of study	Sample size and setting	Mean age (SD) and clinical demographics	Questionnaire and diagnostic tools	Main findings and clinical implications
Archie et al. (2013)	Canada Qualitative; thematic analysis	45 FEP 33M/12F Early intervention Programme, outpatients	Age range 17–35 (median 23 years) No specified mean age or SD Time in early intervention programme < 1 year 22.2% 1 year–23 months 24.4% 2 years 11.11% 4 years 8.8% 5 years 4.4% 6–9 years 4.4%	Focus groups	Participants believed the use of street drugs was a way for them to deal with adversity and to medicate against stress. This approach was endorsed by many. *‘*I would do just about any drug I could get my hands on… I would just go overboard and just get… right messed out of my mind to deal with what was going on inside my head.’
Artaud et al. (2020)	Canada Qualitative; thematic analysis	18 FEP participants 13M/5F 8 SZ, 5 SAD, 5 Bipolar disorder 13 relatives 8 clinicians Early Intervention Clinic, Outpatients	26.8 (No SD specified) Age range 21–37 years Mean duration of psychiatric treatment (months): 36.1 Number of hospitalisations: 2.1	Clinician‐assessed adherence levels Semi‐structured interviews Focus group	Medication non‐adherence: Concerns about side effects Concerns about stigma: ‘Taking medication is more taboo.’ Receiving a diagnosis of psychosis led to an increased negative self‐image. Treatment is associated with loss of control. Mistrust with the clinical team. Viewing psychoeducation as irrelevant to their personal lives. Medication adherence: FEP clients reported that AP medication helped them complete their day‐to‐day tasks which helped preserve their identity. AP medication was viewed as a way to regain control over the psychosis: ‘I feel more in control… fewer things bother me… I'm more on top of things.’ AP adherence was mainly motivated by the intention to please relatives rather than due to an intrinsic motivation. Trust in the mental health team; receiving a non‐judgmental attitude; being viewed as a person helped facilitate adherence. Clinical implications: Themes regarding trust, personal identity and self‐esteem contributed to non‐adherence and need to be explored with clients.
Baker et al. (2015)	Canada Qualitative; thematic analysis	50 EP 3 early psychosis Intervention clinics	Age range 16–30 years Demographic information not obtained	Interviews Focus groups	Several participants discussed cannabis use as a way to self‐medicate, it helped them to sleep, cleared their mind of unwanted and/or confusing thoughts, and generally was used to cope with psychotic symptoms. Other individuals spoke of the negative side effects of psychosis medication and how cannabis temporarily eased some of these effects. Clinical implications: Curbing use early on in the illness trajectory should be an important goal for early intervention services.
Baloush‐Kleinman et al. (2011)	Israel Quantitative; longitudinal, 6 months	112 participants with early episode SZ 70M/42F 84 SZ, 26 SAD Inpatients	30.79 (9.79) Mean number of hospitalisations 3 (SD = 3.82)	Visual Analog Scale for Assessing Treatment Adherence Cognitive Appraisal of Health Scale (CAHS) Scale to Assess Unawareness of Mental Disorder (SUMD) MacArthur Competence Assessment Tool (MacCAT) Drug Attitude Inventory (DAI) Trust in Physician Scale (TPS) Visual Analog Scale for perceptions of family involvement with treatment	Medication adherence: Adherent participants showed significantly higher levels of insight into illness, awareness of the need for treatment, more positive perceptions of doctor patient trust, greater perceived family involvement in pharmacological treatment, and more positive attitudes towards medication in the family. Adherent participants also showed significantly more positive attitudes towards medication. Attitudes towards medication mediated awareness of the consequences of the illness and awareness for the need for medication on adherence. Clinical implications: Attitudes towards medication may provide a key direction for intervention.
Bhikha et al. (2015)	UK Qualitative; content analysis	45 EP 27M/18F 17 participants from EIS, 28 participants from Community Mental Health Teams (CMHT)	Age range 16–65 Mean DUP 77.7 weeks	Short Explanatory Model Interview (SEMI) Psychosis vignette	Medication adherence: The majority of patients held dual explanatory models of psychosis (77.7%), combining prescribed medication and seeing a traditional healer as a treatment method. Clinical implications: The results highlight the importance of possibly working together with traditional healers in the UK to provide a positive support system.
Bjornestad et al. (2017)	Norway Qualitative; thematic analysis	20 FEP 10M/10F Outpatients	25.8 (age range 17–58) Mean DUP 26.5 weeks (range 0–156)	Semi‐structured interviews	Medication non‐adherence: Longer term AP use and ward admission triggered expectation of stigma from others and self‐alienation. Using medication beyond the acute phase interfered with perception of individual effort as a part of recovery. ‘It's not easy to separate out what is your own contribution when using pills.’ Discontinuation after the acute phase due to considerable physical and cognitive adverse effects. Sense of perseverance, determination and their experience of having withstood their acute symptoms. *‘*If I can manage this without pills, I'd rather do that.’ Effects of medications compromised daily life functioning. *‘*This can't go on.’ Disproportionate focus on AP described as being in conflict with their idea of recovery, resulting in resistance and mistrust. Medication adherence: Medication necessary during the acute phase to stabilise mental chaos, reduce distress, and positive symptoms. Detailed information before commencement and establishment of trust between client and professionals, enhancing participants' perspectives of control. Non‐stigmatising, inclusive atmosphere reduced stigma and self‐alienation in longer periods of hospitalisation. Social network described as non‐stigmatising, *‘*it was okay to have a mental illness.*’* Clinical implications: This study highlights the acute shift in perceived usefulness between the acute phase and later stages of illness. Findings support the development of a system‐wide implementation of safeguards to frequently monitor clients' experiences and wishes in relation to AP use.
Brown et al. (2013)	UK Qualitative (thematic analysis) and quantitative (mirror‐image design) methods	35 EP (25M/10M) 20 Mental Health Workers Early Intervention Services, outpatients	25 (5)	Red, amber, green relapse zoning system Service use data Interviews	Medication non‐adherence: Two of five participants said that if workers had adopted a paternalistic approach, they would have stopped taking their medication. A number of patients felt that the part medication plays in aiding recovery is overplayed. Medication adherence: Most patients interviewed talked about the value of being invited to be open with mental health workers about their experiences of taking psychiatric medication. Patients talked about how medication had stabilised their ‘mood’, and this had a positive impact on many areas of their lives. To enable evidence‐based interventions to be translated into routine practice, clinicians will need to acquire the necessary competencies to do this work. Training is perhaps the only realistic way of achieving this. The evidence presented informs a view that AT training for early intervention in psychosis teams should be routinely available.
Choi and Kweon (2023)	South Korea Quantitative; cross‐sectional	166 EP 79M/87F Outpatients	27.76 (7.66) Frequency of hospitalisation after onset None 41%, Once 44.5%, ≥ twice 14.%	Drug Attitude Inventory (DAI) Drug‐related self‐efficacy scale Medication Adherence Rating Scale (MARS), Korean version	Medication adherence: Medication adherence was positively correlated with drug attitude (*r* = 0.393) and medication adherence self‐efficacy (*r* = 0.697). Drug attitude was positively correlated with medication adherence self‐efficacy (*r* = 0.334). Drug attitude and medication adherence are partially mediated by medication adherence self‐efficacy. Clinical implications: Healthcare professionals should adopt a personalised medication education approach that considers individual drug attitudes. Medication adherence in patients with early psychosis should be encouraged by increasing medication adherence self‐efficacy. Interventions aimed at enhancing medication adherence self‐efficacy may indirectly augment the impact of drug attitudes on medication adherence. Medication adherence self‐efficacy will have a positive effect in patients with early psychosis who are in a critical period of clinical treatment.
Chua et al. (2024)	Singapore Qualitative; thematic analysis	12 FEP 8M/4F 12 Caregivers 6M/6F Outpatients	FEP, 29.4 (6.5) Caregivers, 54.8 (10) Number of inpatient admissions None 33.3%, One 25% Two 25% Three 16.7% DUP (months) Mean 16.6 SD 33.7	Semi‐structured interviews	Medication non‐adherence: Service users displayed an unwillingness to adhere to prescribed treatments, expressing preferences such as deciding when to take medication. Service users were confident in their ability to self‐treat through willpower and self‐research, without external intervention. Improved confidence, academic success, and a perception of stability were cited as reasons for discontinuing treatment. Adverse events negatively impacted service users resulting in them opting to avoid future unfavourable experiences. Examples included medication side effects, traumatic inpatient admissions, dissatisfaction with the hospital environment, and discomfort with changes in the case manager. Lack of quality or sufficiency of information were common concerns – ‘I feel that the doctors don't… can't provide a good enough reason, in my opinion, to stay on the medication.’ Perceived lack of effectiveness in the prescribed medication in symptom resolution. Not believing in the medical model—Attributing problems to Islamic mythological creatures like jinns and bad spirits. Scepticism about therapy and drugs, advocating for alternative methods. Clinical implications: It is important to keep an open mind to understand what personal recovery means to the individual service user, so that treatment goals can be better harmonised.
Cowan et al. (2020)	Canada Qualitative; thematic analysis	24 FEP 16M/6F/2T Early Intervention Service	22.67 (No SD specified) Age range 17–34	Semi‐structured interviews	Medication non‐adherence: Trepidation about using medication: ‘At first, because I didn't believe in taking medication it was kind of hard. So, at first, I never used to take it.’ Participants had negative initial impressions or experiences and requiring further research or medication changes until they felt comfortable enough to take medication. Taking the medication required remembering the pill daily, or attending appointments for an injection, and balancing side effects with concerns of relapse. If the perceived benefits did not outweigh the perceived costs, the medication was sometimes deemed unnecessary. Saw the medication not only as unnecessary, but also as symbolic of the psychiatrist's disregard for his concerns: *‘*I told [the psychiatrist], face to face, I feel better, I don't need the medication, and he still gave me a prescription. So, I threw it in the garbage.’ A prescriptive stance from providers can feel disempowering. Participants described holding preconceptions about psychiatric medication that were deeply influenced by mental illness stigma. Medication adherence: The authority of the treating team could be experienced as supportive and collaborative. Stigmatised views of medication were countered by information and experience. Clinical implications: Relationships with service providers constitute a critical structure. The quality of these relationships depends on an individual's needs for structure, authority, autonomy, and contact, all of which may evolve during their journey through treatment. Fostering individuals' autonomy is essential as it can support shared decision‐making in services.
Dijkstra et al. (2024)	Netherlands Qualitative; interpretative phenomenological analysis	14 FEP 10M/4F early intervention services	Age range 22–56 (No mean or SD specified)	Interviews	Medication non‐adherence: Loss of energy, feeling drowsy, and sedated in day‐to‐day life. In a *‘*persistent vegetative*’* state. Self‐stigma because of taking antipsychotics, *‘*only suckers take those pills.*’* Impact on identity—*‘*I became a completely different person… Despite all the negative experiences that happened before, I decided to stop the medication*’* Medication adherence: Feelings went from *‘*very chaotic to just calm*’* and AP helped them to *‘*experience things a little less intense.*’* Medication helped them not to *‘*disappear in their thoughts as deep as before.*’* Participants regained their personal capacity for thinking well again. They were able to organise their thinking. Clinical implications: Clinicians should discuss less obvious aspects of experience concerning recovery with antipsychotic medication.
Eisner et al. (2014)	UK Qualitative; thematic analysis	23 EP participants 11M/12F 17 SZ, 6 SAD Inpatients and outpatients	38.4 (14) Number of psychotic episodes Two 17.4% Three 52.2% ≥ Four 30.4%	Interviews	Medication non‐adherence: Feeling better after stopping the medication Forgetfulness–‘I notice even if I miss a couple of my tablets… I'm really forgetful sometimes I forget to take them.*’* Medication adherence: Reducing medication after feeling well, participants expressed regret about having done so. *‘*That was the worst thing I could have done.*’* Those who sought help early on were most likely to talk to family or friends. ‘[partner's name] will say like um ‘have you taken your meds’ and stuff like that…’ Clinical implications: Early signs interventions need to be individually tailored. Use early signs of relapse interventions to increase service users' self‐efficacy and sense of control.
Elowe et al. (2022)	Switzerland Quantitative; 3‐year longitudinal	331 FEP 67.4% M/36.6% F Early Psychosis Programme, outpatients	24.5 (4.53) Mean duration of illness (years) 1.04 (SD = 2.4)	Assessor ratings of insight Patient and family‐reported medication adherence	Medication adherence: Transition between the beginning and the end of the program confirms the stability of patients with high insight and adherence throughout the 3 years (91.2%, *n* = 103). Also, 56.8% of patients who start with low insight and adherence are likely to improve, although a gain in adherence is more likely than a gain in insight Clinical implications: Adherence is the first variable to improve but a gain in insight in the longer term possibly plays a role in the reinforcement of adherence. Patients with a high level of adherence and to a lesser extent insight are those with the greatest chance of functional recovery.
Endale Gurmu et al. (2014)	Ethiopia Quantitative; cross‐sectional	209 participants 105M/104F Outpatients	No mean age or SD specified 18–24 12.4% 25–30 45% 31–40 20.1% 41–50 11.5% > 50 11%	Medication adherence rating scale (MARS) Self‐reported reasons for non‐adherence	Medication non‐adherence: In the whole sample, 94 participants discontinued medication without consulting their prescribers for various reasons: Recovery from their illness (26%) Unavailability of drugs (18.1%) Adverse drug reactions (12.7%) Forgetfulness (10.6%) Being busy (8.6%) Seeking alternative therapy (5.3%)
Gray and Deane (2016)	UK Qualitative; thematic analysis	20 FEP Early intervention services	26 (3.4) Mean contact with FEP team (months) 20 (SD 10)	Interviews	Medication non‐adherence: As symptoms ameliorate, people forget the helpful effects of medication. Frustration about side effects and the crushing effect medication had on motivation and communication. The colour of the pill had no meaning to them. The name of the company making the drug made them question taking the medication. Dissatisfaction with the number and taste of the tablets, *‘*they should make it in one tablet.*’* Patients confused the number of tablets with the amount (dose) of medication they were taking. Medication adherence: Medication had completely got rid of their psychotic symptoms stating that ‘the voices have gone.’ Medication had tempered ‘the distress of psychosis.’ Prepared to tolerate extremely distressing side effects because of fear of relapse and admission to hospital, ‘I was so determined not to go back to hospital.’ Clinical implications: Clinicians should pay more attention to practical problems with medication in their day‐to‐day work with patients.
Hickling et al. (2018)	UK Quantitative; cross‐sectional	50 FEP 32M/18F early intervention service, outpatients	28.43 (5.9) Average contact with mental health services (months) Mean 38.8 months, SD 55.9	Selwood Adherence Scale Beliefs About Medicines Questionnaire (BMQ) Satisfaction with Information about Medicines Scale (SIMS)	Medication non‐adherence: A significant effect of BMQ Concerns (personal worries about medication) and BMQ Harm (negative beliefs that medicines are harmful) was found on adherence. In regards to the ‘risk of getting side‐effects’, non‐adherent patients were significantly less satisfied with the information received than adherent patients. Medication adherence: Adherent patients were more satisfied with all aspects of the information provided about medications than non‐adherent patients (65.7% and 34.3% respectively). Clinical implications: Patients are more likely to adhere to medication if they believe the benefits will outweigh the costs. Further research could help determine whether increased information giving would increase satisfaction and adherence. Patients should be offered education early in treatment about side effects, and perhaps signposting to services that could support weight loss efforts.
Hon (2012)	UK Qualitative: Grounded Theory	12 FEP participants 7M/5F 6 SZ, 3 SAD, 3 Bipolar Disorder Early Intervention Service (EIS)	25.17 (3.9) Age range 18–35 years Treatment time 2 months—3 years	Semi‐structured interviews	Medication non‐adherence: Participants reported being adversely affected by the side effects of Aripiprazole. Participants struggled to make sense of the changes caused by the illness. Until participants were able to understand the reason for them to take the medication, adherence tended to be unpredictable. Medication adherence: Quality of life—Medication helped them to regain their independence. Getting back on track with their lives or reintegration to normal living was important. Their desire to live a normal life would influence their approach to medication taking. Health status—Positive treatment effects gave them the incentive to continue their medication. Fear of relapse is a constant reminder. Understanding the changes helped them make decisions in medication taking. Being given information of the nature and effects of the medication helped them to persevere. Participants put up with significant side effects for the benefits. Clinical implications: EIS must develop strategies incorporating more practical, educational, and psychological support. This involves providing sufficient information and support to help patients accept their situation, uncovering patients' beliefs about medication and using their internal and external resources to achieve recovery and normality of function.
Hui et al. (2006)	Hong Kong Quantitative; cross‐sectional	484 participants 229 Early SZ 114M/115F 255 Chronic SZ 132M/123F Outpatients	26.21 (8.42)	Medication Adherence Rating Scale (MARS) Clinician reported adherence	Medication non‐adherence: Feeling embarrassed about medication was significantly related to both forgetting to take medication and deciding to stop medication. Taking medication only when feeling unwell was related to deciding to stop medication. Feeling worse without medication was significantly related to forgetting to take medication. Patients who felt embarrassed about medication were more likely to stop medication. Clinicians significantly under‐recognised embarrassment. Clinical implications: Clinicians should aim to understand patients' concerns about taking medication in front of others and the practical difficulties of taking medication in private. These findings suggest that in addition to medication attitude information, clinicians' evaluations can help to inform non‐adherence behaviour. Clinicians' evaluations are important in the detection of non‐adherence behaviour. With effective detection, intervention to address nonadherence could be implemented.
Hui et al. (2018)	Hong Kong Qualitative; thematic analysis	16 participants (from a previous FEP intervention study) 6M/10F Outpatients	40.5 (9.1)	Semi‐structured interview	Medication non‐adherence: Patients suggested they can stop taking antipsychotics because they felt better. Some commented that antipsychotics had no effect after a long period of time. Side effects adversely affect social functioning. Living alone. ‘Sometimes it will be better if there is someone close by to remind you.’ Medication adherence: Taking medication was key in relapse prevention. Antipsychotics give a sense of security which can keep individuals from relapsing. Family support
Intharit et al. (2021)	Thailand Qualitative; thematic analysis	20 participants 10 FES 7M/3F 10 Caregivers 5M/5F Outpatients	FES, 31.5 (12.9) Caregivers 53.4 (8.5) Average length of mental illness (months) = 9.5, SD = 3.9	Semi‐structured interviews Focus group	Medication non‐adherence: Patients took AP drugs until they felt better. Patients reduced the dose of their medication when side effects occurred. Patients perceived symptoms as neither abnormal nor mentally ill, so no medication was required. Patients and caregivers had negative feelings, such as embarrassment. Patients would not cooperate with treatment or caregiver would not take patient for treatment. Medication adherence: Patients realised importance of treatment. If caregivers were understanding of SZ, and were able to provide good care, they could help support the patient. Caregivers and relatives would help bring patients to treatment while providing care, support, and encouragement to each other.
Islam et al. (2015)	UK Qualitative; thematic analysis	22 EI service users 11M/11F 11 Carers 3M/8F Early Intervention services	22 (No SD specified)	Focus groups	Medication non‐adherence: Cultural beliefs competed with medical explanations. ‘My mother believes that the cure will come from God… and I believe that as well.’ Service users whose religious practice and beliefs forbade the consumption of pork or pork products refused to take medication, which contained such ingredients. Medication adherence: Some service users from Pakistani Muslim backgrounds also continued to visit faith healers after coming to EIS. All service users and their carers held multiple explanatory models of illness which were often interchangeable. Clinical implications: Issues raised in relation to respecting individual's health and spiritual beliefs have relevance for wider community groups. Findings highlight issues and implications for the accessibility, acceptability, and appropriateness of U.K. EI services for all BME groups, which need to be addressed.
Kolliakou et al. (2015)	UK Quantitative; 12 month Observational study	69 FEP 75% M/25% F Inpatients and Outpatients	Median age 25	Self‐reported cannabis use Reasons For Use Scale (RFUS) Urine Drug Test	Medication non‐adherence: Relief of positive symptoms and side effects was an endorsed motive for cannabis use. Clinical implications: The idea that patients take cannabis for relief of positive symptoms or medication side effects cannot be dismissed. Awareness of patients' reasons for cannabis use would allow clinicians to plan psychotherapeutic and pharmacological interventions to target widely endorsed incentives such as relief from psychotic symptoms and medication side effects, which for some patients might still be strong motivators in sustaining their cannabis use.
Lobbana et al. (2010)	UK Qualitative; thematic analysis	19 FEP 15M/4F Early Intervention Service	Median age 23 (No mean or SD specified)	Semi‐structured Interview	Medication non‐adherence: Drugs were a coping mechanism for existing mental health problems. Drug use helped them cope with psychotic symptoms because they felt that drug taking was a less anxiety‐provoking explanation for hearing voices. Clinical implications: These findings support the use of psychological approaches that help people to identify and acknowledge the positive reasons for their drug use, to explore the potential negative effects, including impact on mental health problems, and to find alternative strategies to achieve desired goals. The reliance on knowledge derived from personal experience in forming beliefs about the relationship between drug use and mental health needs to be recognised
Mané et al. (2015)	Spain Quantitative; cross‐sectional	48 FEP cannabis users 79.8% M/29.2% F 48 Controls 62.5% M/37.5% F Early Intervention Services	FEP cannabis users 23.94 (4.12) Controls (cannabis users with no psychosis) 24.9 (3.69) Mean DUP FEP cannabis users (days) 65.05 (SD = 132.29) Controls 147.28 (235.15)	Self‐reported reasons for cannabis use	Medication non‐adherence: A significantly greater proportion of psychotic patients reported ‘arranging my thoughts’ as a reason to use cannabis. Moreover, significantly more patients than controls listed ‘to decrease my hallucinations and suspiciousness.’
Miller and McCormack (2006)	USA Quantitative; 12 month longitudinal study	77 FES 53M/24F Patient setting not specified	23 (No SD specified)	Interviews	Medication non‐adherence: Patient or family did not believe in medication because religion forbids it. Once the patient recovered, the father would not permit him to continue medication. A common theme in treatment was that medication is prohibited by religion. Clinical implications: Faith, as part of patients' lives, cannot be ignored any more than can symptoms, family problems, misuse of substances, ethnicity, race or gender. If clinicians are not open to discussions regarding faith, one may further invalidate the beliefs of patients at a time when their sense of self is destabilised by illness.
Mutsatsa et al. (2003)	UK Quantitative; cross‐sectional	101 FES 79M/22F 89 inpatients, 12 outpatients	Good adherence group, 25.23 (6.32) Poor adherence group, 27.77 (8.06)	Adherence Rating Scale (ARS) Rating of Medication Influences (ROMI) Schedule For the Assessment of Insight (SAI)	Medication non‐adherence: On the Rating of Medication Influences negative attitudes subscale, the poor adherence group had a significantly higher mean score than the good adherence group, indicating that the poor adherence group expressed more negative attitudes towards medication. Negative attitudes towards medication correlated significantly with adherence (*r* = 0.28). Medication adherence: On the SAI, the good adherence group had significantly higher mean scores. Total insight (*r* = 0.55), awareness of illness (*r* = 0.31), correct illness attribution (*r* = 0.31), and need for treatment (*r* = 0.82) significantly correlated with adherence. Clinical implications: If a patient perceives that the benefit of taking medication exceeds that of the cost, then the person is likely to adhere to treatment and vice versa. At the initiation of drug treatment, attitudes towards medication and insight appear more relevant to medication adherence than side effects. Adherence appears to reflect a complex interaction of influences, which may change over time.
Niemeyer et al. (2018).	Germany Qualitative (content analysis) and quantitative (cross‐sectional) methods	64 adolescents with psychiatric diagnoses 29M/35F Inpatients and Outpatients	15.1 Age range 12.01–17.11 years	Semi‐structured interviews Medication Adherence Rating Scale (MARS) Clinician report of medication adherence Questionnaire on Attitudes Towards Treatment (QATT)	Medication non‐adherence: Not completely adherent patients more likely reported: Feeling worse after taking medication: ‘I feel a bit sluggish, like I don't care anymore. Maybe it's got something to do with the medication.’ Lower sense of self‐efficacy concerning the improvement of their symptoms. Less trustful physician‐patient relationship. Worsened attitude towards medication after experiencing adverse events/side effects: ‘It was definitely not the right drug for me, my opinion is completely negative because of the weight gain.’ Less support from their relatives. Fewer individuals in their family who were fully informed about their condition. Clinical implications: The results indicate that certain factors contribute to an increased or decreased medication adherence. These are beliefs about the effectiveness of medication, thoughts about adverse events/side effects, self‐efficacy, trustful physician patient relationship, and family support. Consequently, it may prove useful in clinical practice to address these factors when initiating and monitoring treatment with psychopharmacological medications.
Ocansey et al. (2025)	Ghana Quantitative; cross‐sectional	117 SZ 53.4% M/46.6% F Outpatients (inferred)	38.09 (13.39) Years lived with condition (number of patients) < 1 year (31) 1–3 years (33) 4–6 years (20) > 6 years (32)	Medication Adherence Report Scale‐5 (MARS‐5) Beliefs About Medicines Questionnaire (BMQ‐Specific) Doctor‐Patient Communication (DPC‐13)	Medication adherence: Necessity beliefs about medication and doctor–patient communication were significantly positively associated with medication adherence (Adj. *r* ^*2*^ = 0.44). Concerns about medication were not significantly associated with medication adherence. Necessity beliefs about medication significantly interacted with doctor–patient communication in predicting medication adherence. Concerns about medication did not interact with doctor–patient communication in predicting adherence. The association between necessity beliefs and medication adherence is dependent on the level of doctor–patient communication. Clinical implications: This finding provides opportunities for healthcare practitioners to take patients' beliefs about their treatment into serious consideration during medical consultations practitioners should integrate patients' beliefs about treatment into their diagnosis to achieve optimal treatment and medication adherence. Mental health policymakers can also develop communication guidelines and patient psychoeducational interventions to encourage patients to seek clarifications during medical consultations to ensure they have a shared understanding of the treatment plan.
Penny et al. (2009)	UK Qualitative; Interpretative Phenomenological Analysis	6 EP 11 family members Early Intervention Services	EP 18.5 (No SD) Time with service 1–4 years	Semi‐structured interviews	Medication non‐adherence: The service's preferred stress‐vulnerability model was either not Heard or not understood by service users' families. When medication failed to bring about recovery, or resulted in unpleasant side effects, some families stopped the medication. ‘I thought the pills will help him to become the way he was before.*’* Many family members described religion as a way of coping and making sense of what happened and giving them hope. *‘*I am not really happy with the medicine. The only thing we can believe in is Allah.’ Medication adherence: Parents saw the service (especially the doctor) as the decision maker and expert. *‘*the doctors know better than us.’ Families felt responsible for chasing the EIS, even though it was thought of as reliable. *‘*the key worker never forgets. Anyway, I don't let her forget, I ring. When medicine is finished.’ The belief that there may be a spiritual cause for illness is intrinsically tied up with treatment. Clinical implications: Working with families is especially important for an EIS, which views them as allies in the fight against a potentially severe but treatable mental illness.
Perkins et al. (2006)	USA Double blind RCT, 2 years	254 FEP participants 208M/46F 149 SZ, 79 Schizophreniform disorder, 26 SAD Inpatients and Outpatients	23.85 (4.79) Age range 16.1–39.6 years Mean duration of illness (weeks) 62.29 (SD 60.29)	Rating of medication Influences Scale (ROMI) Insight and Treatment Attitudes Questionnaire (ITAQ) Pill counts Body Mass Index (BMI)	Medication non‐adherence: The likelihood of becoming medication non‐adherent for 1 week or longer was greater in subjects whose belief in need for treatment was less (HR = 1.75) or who believed medications were of low benefit (HR = 2.88). Clinical implications: Illness and treatment beliefs may be targets for interventions to improve adherence. It may be that motivational interviewing and cognitive behavioural therapeutic techniques that are useful in chronic schizophrenia may similarly improve adherence in individuals recovering from a first episode of schizophrenia.
Perry et al. (2007)	UK Qualitative; Interpretative Phenomenological Analysis	5 FEP 5M Outpatients	21.8 (No SD specified) Mean duration of time since FEP (months) 7.2	Semi‐structured interviews	Medication non‐adherence: During their time in hospital participants had no control over what was happening to them and experienced feelings of powerlessness. This was particularly true with regard to the medication they were given. Trying to take control and refuse medication resulted in being forcibly injected, increasing the sense of powerlessness. Participants did not want to see themselves, or be seen by others as being different. Medication adherence: Importance of having social relationships. ‘When you are alone you feel more hopeless’ Importance of getting back to normal. ‘I wanted to prove to the world that I was back to normal.’
Polillo et al. (2022)	Canada Quantitative, 9‐month observational study	167 EP 76M/78F, 79 Family members 18M/61F EIS Service Outpatients	EP, 22.8 (3.46) Family members, 47.8 (12.57)	Service Engagement Scale (SES) Working Alliance Inventory (WAI) Scale To Assess the Therapeutic Relationship in Community Mental Health Care: Patient Version (STAR‐P) Electronic Health Records (EHR)	Medication non‐adherence: Medication side effects was the top patient and family‐reported barrier to engagement, endorsed by 28.7% and 39.2% respectively. Forgetting or losing track of appointments (25.7%) Stigma related to coming to a hospital (24.0%) Past experiences with services (21.0%) Feeling uncomfortable leaving the house or going to an unfamiliar place (18.6%) Other top family reported barriers included patients wanting to address problems without professional help (16.5%) and disliking or not trusting the clinician (17.7%). Medication adherence: Patient‐reported facilitators of engagement highlight: The importance of feeling understood by their clinician (36.5%) and agreeing on a treatment (34.1), as well as having discussions about personal goals and thoughts about treatment (43.7). Patient self‐reported motivation and commitment to treatment (41.9%). Family‐reported facilitators: Patients having a positive impression of the clinician (43%) Patients' level of motivation and commitment to treatment (36.7%) Believing treatments are helpful (36.7%). Clinical implications: These findings lend support for shared decision making, leveraging standardised assessments of medication and side effects, and psychoeducation on the risks of medication nonadherence. Individual resiliency training provides an opportunity to build trust with patients. It may also be helpful to implement a more targeted approach to identify, stabilise, and actively engage patients early in treatment who may be vulnerable to disengaging, using strategies to develop the therapeutic relationship, building motivation to use individual resiliency training through motivational interviewing, and providing education about and close monitoring of medication side effects. Focusing efforts on methods for managing medication side effects and encouraging use of IRT early in care to build the therapeutic relationship may help facilitate early engagement.
Quach et al. (2009)	Denmark Quantitative; 2 year RCT	547 FEP 323M/224F Inpatients and Outpatients	26.6 (6.4) Mean DUP (weeks) 103 (SD = 175)	Scale to assess Unawareness of Mental Disorder (SUMD) Rating of Medication Influences (ROMI) Assessor‐rated medication adherence	Medication non‐adherence: At 2‐year follow up, patients in the poor adherence group were less aware of mental disorder, of the effect of medication and of the consequences of the mental disorder 1 year prior. Medication adherence: The group with good adherence at 1‐year follow up had better insight into illness at entry in all three dimensions in the univariate analyses. They were more aware of mental disorder, of the effect of medication and of the consequences of mental disorder. They also had a significantly more positive attitude towards medication than the poor adherence group. Clinical implications: To enhance medication adherence, more specific interventions, with cognitive and behavioural techniques to develop a positive therapeutic alliance and a positive attitude towards medication, should be given to patients.
Rathod et al. (2023)	UK Qualitative; thematic analysis	21 FEP 10M/11F Early Intervention Services	25.4 (5.5)	Semi‐structured interviews	Medication non‐adherence: Previous experiences with services affect the level of trust they have. Some cultures and religions prevented individuals talking about their difficulties. Medication adherence: Religious community beliefs differed from patients' understanding and that of mental health professionals. ‘the church I was going to were convinced that your healing came from Jesus and that medication, you shouldn't need it… I disagree completely.’
Sapra et al. (2014)	USA Quantitative, cross‐sectional	49 participants 33 FEP 73% M/27% F, 16 Multi‐episode 68% M/32% F Inpatients and Outpatients	FEP 25.8 (6.7) Multi‐episode 33.7 (9.3) Duration of illness (years) 0.8	Rating of Medication Influences (ROMI) scale	Medication non‐adherence: The top subjective reasons for nonadherence reported were: Distress from side effects (32.4%) No benefits from medication (26.5%) Denial of illness (26.5%) Stigma (26.5%) Interferes with life goals (20.6%) Change in appearance attributed to medication was more frequently endorsed by the multi‐episode sample as compared to the first‐episode sample (25% vs. 0%). Multi‐episode patients were more likely to report that medications were unnecessary (31.3% of multi‐episode vs. 8.8% of first‐episode patients). Medication adherence: The top adherence influences reported were: Doctor patient relationship (76.5%) Relapse prevention (73.5%) Day‐to‐day (immediate) benefit from medication (44.1%) Help with life goals (32.4%) Family support (32.4%) Doctor‐patient relationship was much more frequently endorsed by the FEP sample compared with multi‐episode patients (76.5% vs. 12.5%). There was a significant difference in clinician alliance (non‐prescribing), with 18% of FEP patients considering it as a strong influence and none of the chronically ill patients considering it of any influence. There was also a significant difference in the endorsement of ROMI item ‘help with life goals’ (32% vs. 0%). Clinical implications: Strategies to help strengthen the therapeutic relationship with treating clinicians might be particularly influential for individuals early in the illness course. Establishing an alliance with younger FEP patients at their initial contact with the mental health system has the potential to impact adherence behaviour in the long term. Improved adherence may come through helping FEP patients identify the role of medications and treatment in fulfilment of their life goals. Assessing educational, social relationship and occupational goals from the beginning and connecting them with treatment and relapse prevention in discussions and psychoeducation sessions with the patient will help initiate and maintain adherence.
Sheridan Rains et al. (2024)	UK Qualitative; thematic analysis	20 EP 18M/2F Early Intervention Services	26.1 (No SD specified)		Non‐adherence Using cannabis to suppress the short‐term symptoms.
So et al. (2024)	China Quantitative, cross‐sectional	199 EP participants 38.7% M/61.3% F SZ 119, Schizophreniform disorder 30, SAD 4, Brief Psychotic disorder 17, Delusional disorder 17 Outpatients	41.7 (7.9) Mean (SD) age at onset of psychosis Adherent 36.5 (7.8), non‐adherent 35.5 (8.6) Mean DUP (days) Adherent 153, non‐adherent 245	Scale to Assess Unawareness of Mental Disorder (SUMD) Self‐Stigma Scale‐Short Form (SSS‐S) Patient Satisfaction Questionnaire (PSQ) Medication Compliance Questionnaire (MCQ)	Medication non‐adherence: Greater self‐stigma and negative medication attitudes were significantly associated with medication non‐adherence in early psychosis patients, with odds ratios of 0.921 and 2.657 respectively. Clinical implications: Development and provision of interventions specifically targeting at potentially malleable risk factors including insight to illness, self‐stigma and therapeutic alliance may significantly reduce medication non‐adherence and rectify negative attitudes in early psychosis.
Stewart (2013)	Australia Qualitative; Grounded Theory	30 FEP 15M/15M Early Intervention Program, Outpatients	Age range between 18 and 20 years (Mean and SD not specified)	Interviews	Medication non‐adherence: Participants developed negative views about treatment as a result of the poor relationships with hospital care providers. Experiences during hospitalisation exacerbated the symptoms of psychosis, decreased their ability to cope, and left them humiliated at the time of discharge. Medication adherence: Successful engagement was attributable to relationships in which clinicians taught them about the illness, guided them through treatment, identified and supported their personal strengths, and instilled an optimistic view of the future. They described these clinicians as genuine, unconditionally accepting, and comfortable with personal closeness. Belonging to a peer group gave them a sense of friendship, an opportunity to talk in a positive and nonjudgmental atmosphere, and the chance to experience a collective determination towards recovery.
Stürup et al. (2023)	Denmark Quantitative, Prospective cohort study with post hoc study design	215 FES 105M/110F Early Intervention Services, Outpatients	25 (4) Mean DUP (weeks) Discontinuation group 176 (SD 200), Continuation group 132 (174)	Reasons for Antipsychotic Discontinuation/Continuation from Patient's Perspective (RAD‐I)	Medication non‐adherence: The three most frequent primary reasons for discontinuation were: Side effects (38.2%) Patient believed he/she no longer needed the medication because he/she was now better (18.4%) Other reasons (15.8%) Medication adherence: The most frequent primary reasons for continuation were: Benefits for positive symptoms (64.7%) Another person told this patient to continue taking their medication (7.9%) Other reasons (6.5%) Clinical implications: Discontinuation of antipsychotics is common, which stresses the importance of knowledge of reasons, identifying individuals with increased odds of discontinuing, discussing the (dis)advantages of discontinuation in order to make an informed shared decision, and helping with a safe discontinuation. Individuals with first episode schizophrenia, their relatives, and clinicians may use this knowledge in shared decision making, improvement of adherence, and prevention of high‐risk discontinuation.
Tan et al. (2019)	Singapore Quantitative; longitudinal study	280 FEP 142M/138F Early Intervention Program	Age range (years) 15–17 3.6% 18–24 46.1% 25–30 25.7% 31–40 24.6% Mean DUP (months) 13.6 (SD = 21.7)	Participants' expectancies of alcohol (self‐report)	Medication non‐adherence: Binge drinkers were significantly more likely to endorse statements relating to coping with distress. The prevalence of binge drinking among the FEP sample was relatively high. Clinical implications: Findings reinforce a motivation which interventions could target to reduce reliance on drinking.
Vaingankar et al. (2020)	Singapore Qualitative; thematic analysis	40 FEP 13M/27F Early Psychosis Intervention Program	27 (No SD specified) Age range 18–39 Duration of illness 5 months‐6 years (62.5% diagnosed < 2 year before focus groups)	Focus group discussions (FGD)	Medication non‐adherence: Personal challenges, stigma and medication side effects formed a considerable part of their recovery journey. ‘Family, friends, sometimes your employers also, your colleagues. They tell you to get off medication, to them it's an expectation of recovery but it's not realistic. And it affects you because it affects your sense of self and what you are able to do.’ Not being on medications was mentioned during the FGDs and perceived as a benchmark for recovery by some participants. ‘I have a problem with medication to be honest. Because I think when you are made to take medication like, there's this constant reminder like for me I'm still on medication and I'm technically supposed to be taking it every night but I kind of do it alternate night because I don't feel like I actually need it.’ Medication adherence: Being disciplined in taking one's medications on time and having awareness of the effects of medications on the self were also highlighted. Participants strongly advocated the need to have a positive and optimistic perspective on life and its challenges, as well as being resilient and independent. Engaging with others (even if initially distressing) and communicating personal problems with people that they trusted were also deemed as taking personal agency over their recovery. Participants talked about the trust they had in their psychiatrists as an expert who knew best about the efficacy of medications.
van der Heijden‐Hobus et al. (2024)	Netherlands Qualitative; interpretative phenomenological approach	20 FEP 9M/11F Outpatients	Age range 20–60 (No mean or SD specified)	Semi‐structured interviews	Medication non‐adherence: More information might have helped participants to be more motivated to take the medication. ‘I was very suspicious of the medication, so I did not want to take it.’ Participants felt that they would have needed additional information about medication. Medication adherence: Participants mentioned that feeling safe was one of the most important needs during the psychotic episode. Medication helped in this regard. Feeling understood by health care providers helped them to understand themselves ‘I realised that I need medication and using cannabis is not good for me.’ Participants appreciated that they received practical guidance from relatives. Participants also indicated that medication could help with regaining control of their lives. They advised against cannabis. Others described the support of significant others as very helpful, also for making important choices in treatment. ‘A friend persuaded me to take my medication, not my physician’ Most participants recollected information was given about duration of treatment, mechanism of action, and side effects. Clinical implications: Providing a tranquil environment, a dedicated spokesperson, and improved psychoeducation on several topics at the right time might address the unmet needs revealed in this study. Also, the integration of important others (family and close friends) in the care process during hospitalisation could be improved.
Yeisen et al. (2017)	Norway Qualitative (thematic analysis)	20 FEP 7M/13F SZ 6, SAD 3, affective psychosis 2 Delusional disorder 2, Psychotic disorder 4, Drug induced psychosis 3 Early Intervention Services, Outpatients	24.6 (No SD specified) Age range, 16–40 years Mean number of hospital admissions 2.5	Semi‐structured interviews	Medication non‐adherence: First meeting with staff had a significant impact in giving a sense of powerlessness. Compulsory admission was seen as adding to the mental exhaustion caused by participant's psychotic condition. Lack of insight caused a discrepancy between perceived symptom levels and symptom level assessment made by psychiatrists, causing reduced willingness to adhere. Isolation, being prohibited from exiting the ward, delay of medical assessment, and lack of time with consultations during the first 2–3 days reduce adherence. Insufficient information about side effects evoked negative emotions, resulting in unfavourable alliances with staff. The expected duration of AP use was unclear. Lack of involvement reduced trust in staff as well as adherence. Experiencing only problematic side effects in the absence of any positive effects. Side effects were the main barrier. Medication adherence: Patients saw admission as necessary to counteract a severe psychotic condition. They felt safe and warmly welcomed, perceiving staff members as supportive and open‐minded. Thorough information from professionals about AP effects. Other patients' positive experiences with AP use could increase adherence. Information about expected duration perceived as having a positive influence. Experiencing the desired effect of the AP Fear of potential psychotic symptom relapse which might result from medication reduction or discontinuation. Experiencing deterioration after discontinuation. Clinical implications: Initial contact with the patient is the key to the creation of an empowering alliance. Information about medication should be repeated once the person is out of the acutely psychotic stage of illness, as an interactive process throughout the course of treatment to strengthen the shared decision making element of the treatment process.

Abbreviations: AP, antipsychotics; DUP, duration of untreated psychosis; EIS, early intervention service; EP, early psychosis; F, female; FEP, first episode psychosis; FES, first episode schizophrenia; M, male; RCT, randomised controlled trial; SAD, schizoaffective disorder; SD, standard deviation; SZ, schizophrenia; T, trans.

**TABLE 5:** Psychosocial interventions to promote medication adherence in psychosis (*n* = 34).

Author (year)	Country and type of study	Intervention, sample size, setting and follow‐ups	Mean age (SD) and clinical demographics	Questionnaire and diagnostic tools	Main findings and clinical implications
Alinaitwe et al. (2024)	Uganda Controlled pilot study	Family Psychosocial Involvement Intervention (FAPII) (*n* = 30) versus Standard Care (*n* = 30) FAPII consists of psychoeducation, family therapy, and open discussion of agreed topics (e.g., medication) One monthly group session (1–2 h) over 6 months 60 participants with severe mental illness (SMI), including 11 with SZ diagnosis, 49 family members Outpatients 6‐ and 12‐month follow‐ups	Intervention group 36.7 (13.5), control group 38.2 (11.6)	Medication Adherence Rating Scale (MARS) Manchester Short Assessment of Quality of Life (MANSA)	There was statistically significant improvement in medication adherence in the intervention group at 6 months and this was sustained at 12 months. High MARS scores were a significant factor affecting quality of life of the respondents at 6 months and at 12 months. FAPII should be instituted in the psychotherapy of SMI patients. A larger randomised clinical trial would be needed to prove the actual effectiveness of FAPII.
Anderson et al. (2010)	USA Single‐blind RCT	Adherence therapy (AT) (*n* = 12) versus TAU (*n* = 14) AT consists of MI and CBT techniques, emphasising a collaborative approach and providing information to service users on their illness and treatment Weekly, individual sessions (20–60 min) over 8 weeks 26 SZ 79% M/21% F Outpatients, community mental health centre 8‐week follow‐up	29 (13), age range 21–57 years Baseline number of hospitalisations zero = 4, 1–10 = 12, 11–20 = 2, 21–30 = 4, ≥ 31 = 1	Adherence Therapy Patient Satisfaction Questionnaire (ATSAT), personal Evaluation of Transitions in Treatment (PETiT) Positive And Negative Syndrome Scale (PANSS)	Patients receiving AT did not significantly improve in overall psychiatric symptomatology or with medication adherence compared with the TAU group at follow up. Although, AT did not result in a statistically significant improvement in symptoms or medication adherence, evidence of active clinical engagement in treatment occurred. The documentation of positive patient feedback with AT might be the ultimate contribution of this randomised controlled trial, suggesting that an important intervention effect is enhanced patient experience. Strategies that dovetail decrease in symptoms, increases in medication adherence, engagement adherence in the therapeutic process, and patient satisfaction could provide a clearer picture of outcomes that influence the successful management of schizophrenia.
Barkhof et al. (2013)	Netherlands Single‐blind RCT	Motivational interviewing (MI) (*n* = 55) versus Health Education (HE) (*n* = 59) MI intervention provided psychoeducation; explored attitudes and beliefs towards treatment; patient's goals; and their readiness for change 5–8 sessions 114 participants (87 SZ, 27 SAD) 91M/23F Inpatients and outpatients 6‐ and 12‐month follow‐up	35.9 (10.3) Mean duration of illness (years) = 7.8 (SD = 6.4) Baseline mean number of psychiatric admissions = 3.8 (SD = 4.2)	Medication Adherence Questionnaire (MAQ) Life Chart Schedule (LCS) Drug Attitude Inventory (DAI) Positive And Negative Syndrome Scale (PANSS)	At both follow‐up assessments, there were no significant differences between MI and HE on the two adherence measures. Likewise, there were no differences in attitudes towards medication. However, MI resulted in reduced hospitalisation rates for female patients, non‐cannabis users, younger patients, and patients with shorter illness duration. This study shows that an adapted form of MI does not produce a significant effect on medication adherence or hospitalisation, compared with HE in a group of nonadherent patients with multi‐episode schizophrenia. However, the results provide indications that MI may yet be suitable for improving adherence in female patients, non‐cannabis users, younger patients, and those with shorter illness duration. Therefore, targeted use of MI may be of benefit for improving medication adherence in certain groups of patients, although this needs further examination. Furthermore, these findings underscore the need to focus on specific targets that lead to nonadherence and to apply an individualised approach for each patient of this challenging group.
Bhawana et al. (2022)	India Quasi‐experimental study	Family psychoeducation, Psychoeducation consisted of improving illness awareness; treatment adherence; problem‐solving skills; communication skills; and caring for the caregivers 5 group sessions (60 min) over 2 weeks 60 patients (44 diagnosed with psychotic disorders, 16 with mood disorders) 35 M/25F; 60 caregivers 32 M/28F Inpatients 4‐week follow‐up	Patients = 33.97 (13.35) Caregivers = 43.02 (11.87) Total duration of illness (years) < 2 = 20 2–5 = 19 > 5 = 21	Medication Adherence Rating Scale (MARS) Rosenberg Self‐esteem Scale Zarit Burden Interview‐22	A significant increase in drug compliance (35%) and self‐esteem (31%) and a decrease in caregivers' burden (13%) were observed after the administration of family psychoeducation. The study results proved that a brief, structured, family psychoeducation effectively reduces noncompliance and caregivers' burden and increases self‐esteem among psychiatric inpatients. There is a great need to provide family psychoeducation on a routine basis in general hospitals. Patients with higher self‐esteem will ultimately show improved social and occupational functionality resulting in satisfactory treatment compliance and decreased caregivers' burden.
Brown et al. (2013)	UK Qualitative (thematic analysis) and quantitative (mirror‐image design) methods	Adherence therapy (AT) AT sessions consist of exploring patient beliefs about treatment, problem‐solving, reviewing past treatment/illness experiences, exploring ambivalence about taking medication; testing medication beliefs; considering life goals and the role of medication in achieving these Six sessions (1 day) over 6 months 35 EP (25M/10M) 20 Mental Health Workers Early Intervention Services, outpatients Baseline test and 1 year post‐intervention follow‐up	25 (5)	Red, amber, green relapse zoning system Service use data Interviews	In year 0, there were 20 relapses that reduced to nine in year 1, a reduction that was statistically significant, equating to a medium effect size (0.33). The relative risk (RR) was 1.73, suggesting that the risk of relapse was 73% higher in year 0. To enable evidence‐based interventions to be translated into routine practice, clinicians will need to acquire the necessary competencies to do this work. Training is perhaps the only realistic way of achieving this. The evidence presented informs a view that AT training for early intervention in psychosis teams should be routinely available.
Budiono et al. (2021)	Indonesia Open label RCT	Family Psychoeducation (*n* = 32) versus Controls (*n* = 32) Six educational videos provided every 2 weeks over 12 weeks in total Videos topics related to stigma in SZ; current therapies; expressed emotion; family/caregiver challenges; and patients' mood states 64 caregiver (M38/F26) and SZ patient (M43/F21) pairs Inpatients and outpatients Baseline test and 12 weeks post‐test evaluation	SZ patients 34.03 (7.05) Mean length of patient contact with mental health services 6.19 years (SD = 0.96)	Indonesian Medication Adherence Scale (IMAS) Illness Perception Questionnaire for Schizophrenia Relatives (IPQS‐R) Five minute Speech Samples (FMSS)	Medication adherence in both the control and intervention groups was low prior to intervention. In contrast to each other, the control group did not show any difference, while the intervention group showed a significant increase in medication adherence (*d* = 1.21). Psychoeducation had a positive impact on patients' adherence, with a significant increase in medication adherence post‐intervention. Caregivers' expressed emotion has a higher negative effect on medication adherence than caregivers' illness perception, although both were found to be significant contributors to medication adherence improvement of both the illness perception and expressed emotion of caregivers is crucial to improving patients' medication adherence. This study demonstrates the effectiveness of psychoeducation and highlights the need to custom‐tailor this to families and patients with schizophrenia in Indonesian communities.
Byerly et al. (2005)	USA Open‐label trial	Compliance therapy (CT) 4–6 individual sessions, 30–60 min over 3 weeks CT combines aspects of MI, psychoeducation, and CBT approaches, focusing on patients' illness/treatment history; beliefs and understanding of the illness; ambivalence towards treatment; and stigma 30 (21 SZ, 9 SAD) 20M/10F Outpatients Baseline test (month −1), intervention (month 0), and monthly follow up (until +6 months)	39.2 (8.3)	Medication Event Monitoring System (MEMS) Clinician‐rated adherence Medication Adherence Rating Scale (MARS) Drug Attitude Inventory (DAI)	The primary study outcome of electronically measured adherence declined 4% from baseline (month −1) to month +1 and then increased by 0.19% per month thereafter. Neither the initial decline nor the subsequent increase were significant. Clinician ratings of adherence did not change significantly during the study. The clinician‐rated adherence decreased by 1.6% from baseline to end of month +1, then decreased by 0.3% per month during the rest of the study. Patient ratings of adherence did change significantly between the baseline and end of month +1 evaluation, but not during the follow‐up period. Patient ratings increased by 8.9% from baseline to end of month +1, then decreased by 1.4% per month during the rest of the study. Attitudes towards medication taking as assessed by the DAI were unchanged. The findings in this uncontrolled trial suggest that this intervention may not benefit patients with psychotic disorders.
Can and Budak (2025)	Turkey Quasi‐experimental design	Cognitive behavioural therapy‐based psychoeducation (*n* = 33) versus controls (*n* = 40) 8 group sessions, 90 min, twice weekly over 4 weeks 73 SZ 49M/24F Outpatients Baseline test and 6‐month post‐test	No mean age, SD specified Age ranges 18–28 (*n* = 12) 29–39 (*n* = 15) 40–50 (*n* = 8) 51–65 (*n* = 5) Duration of illness (years) 0–5 (*n* = 5) 6–11 (*n* = 9) ≥ 12 (*n* = 26)	Morisky Medication Adherence Scale (MMAS)	The medication adherence level of the experimental group before the training was 2.75 (±1.25), and after the CBT‐based psychoeducation, it was 3.57 (±0.67). The medication adherence level of controls before the training was 2.05 ± 1.33, and 2.17 (±1.36) at post‐test. CBT‐based psychoeducation caused a significant difference in medication adherence. It is recommended to include CBT‐based psychoeducation to increase medication adherence. Considering the scarcity of similar studies, it may be recommended to conduct experimental studies evaluating the effectiveness of interventions applied to improve medication adherence, to conduct more experimental studies investigating the effect of CBT on medication adherence and to conduct randomised controlled trials to assess effectiveness.
Chien et al. (2024)	Hong Kong Assessor‐blind, three‐arm RCT	Acceptance‐based, insight inducing medication adherence therapy (AIM‐AT) (*n* = 42) versus conventional psychoeducation group (CPG) (*n* = 42) versus TAU (*n* = 42) AIM‐AT comprised of 10 group sessions, 2 h, weekly/biweekly AIM‐AT sessions focus on understanding illness and treatment; exploring illness/treatment beliefs; history of treatment/illness; reviewing goals and adherence behaviours; evaluating future plans 126 EP participants 87M/39F SZ and schizophreniform disorder (*n* = 45), Delusional disorder (*n* = 15), SAD (*n* = 22), Brief psychotic disorder (*n* = 23), Other (*n* = 21) Outpatients Pre‐test (Time 0), immediate post‐test (Time 1), 6‐(Time 2) and 12‐month (Time 3)post‐test	AIM‐AT 26.9 (5.2) CPG 26.4 (6.7), TAU 27.9 (7) Duration of illness (months) AIM‐AT 16.84, CPG 18.67, TAU 19.28	Adherence Rating Scale (ARS) Insight and Treatment Attitude Questionnaire (ITAQ) Drug Attitude Inventory (DAI) Rehospitalisation rates Eight‐item Client Satisfaction Questionnaire (CSQ‐8)	Compared with the TAU and/or CPG, the AIM‐AT group reported significantly greater improvements in mean scores of medication adherence (ARS) at Times 1–3 (mean difference = 0.22, 0.40, and 0.41). The AIM‐AT group also had significantly greater improvements in psychotic symptoms, psychosocial functioning, service satisfaction, length of rehospitalization, and total number of patients hospitalised over the follow‐up period. These positive findings suggest that the AIM‐AT should be integrated into routine community mental health services for adults with early‐stage psychosis who have mild to moderate symptoms and use antipsychotic medication. Further research is recommended to test the AIM‐AT program for psychotic patients with diverse socio‐demographic and clinical characteristics across different cultures/countries with a longer‐term (e.g., 18–24 months) follow‐up.
Chien et al. (2015)	Hong Kong Single‐blind RCT	Adherence Therapy (AT) (*n* = 57) versus TAU (*n* = 57) 8 sessions, 2 h every 2 weeks over 4 months AT consists of MI and CBT techniques, addressing medication concerns; psychoeducation regarding illness/treatment; stigma; family and social support 114 participants 59M/55F Early SZ (*n* = 71), Other psychotic disorders (*n* = 43) Outpatients Baseline measurement (Time 0), immediate post‐tests (Time 1), and 6‐months (Time 2) after intervention	AT, 29.21 (9.64) TAU, 28.13 (8.96) Duration of illness (months) AT 19.91 (SD 11.8) TAU 20.42 (SD 10.38)	Adherence Rating Scale (ARS) Insight and Treatment Attitude Questionnaire (ITAQ) Positive And Negative Syndrome Scale (PANSS) Rehospitalisation rate	The majority (87.9% in AT and 85.9% in TAU) were deemed totally non‐adherent or poorly to inadequately adherent to medication in both the AT and TAU groups (mean score of 1.48 and 1.39; SD = 0.98 and 1.01, respectively) at baseline. The AT group had significant greater improvements over time than the TAU group on the participants' ITAQ, PANSS, SLOF, ARS, and average number of re‐hospitalizations. In addition, the medication adherence of the AT group showed a significantly greater improvement over time, with a large effect size of 0.72, when compared with the TAU group. Medication adherence rate significantly increased at both T1 and T2 (mean difference = 0.55 and 1.60). Motivational interviewing‐based AT for people with schizophrenia can be effective to reduce symptom severity and re‐hospitalizations, and improve medication adherence, functioning, and insight into illness and/or treatment over a medium term (6 months) period of follow‐up.
Chien et al. (2016)	Hong Kong Single‐blind RCT	Adherence Therapy (AT) (*n* = 67) versus TAU (*n* = 67) 6 sessions, 2 h every 2 weeks over 12 weeks AT consists of MI and CBT techniques, addressing medication concerns; psychoeducation regarding illness/treatment; stigma; family and social support 134 participants Early SZ (*n* = 80) Other psychotic disorders (*n* = 54) 71M/63F Outpatients Pre‐test (T0) 2 weeks (T1), 6 months (T2), and 18 months (T3) post‐tests	AT, 29.13 (9.87) TAU, 28.23 (9.23) Mean duration of illness, months (SD) AT 27.71 (10.1) TAU 28.12 (10.08)	Adherence Rating Scale (ARS) Insight and Treatment Attitude Questionnaire (ITAQ) Hospitalisation rate	When compared with TAU, AT participants had significantly greater improvements at the post‐tests on medication adherence rate (ARS score), which significantly increased at T2 and T3 (mean differences = 0.5 and 1.0; SE = 0.1 and 0.2, respectively). This effective intervention is particularly important to patients who would have a high relapse or recurrence rate (70%–90%) over the first few years after discharge from hospital or acute stage of illness. AT can be a systematic, multifaceted and client‐centred therapy model with evidenced benefits and applicability to outpatients with schizophrenia spectrum disorders in a Chinese population. This therapy can also be provided by other mental health professionals if appropriately trained and become an integral part of the community based rehabilitation programme provided by outpatient care service, together with pharmacological and other psychiatric treatments.
Dahan et al. (2016)	Israel RCT	Tailormade Integrative One‐on‐One Intervention (*n* = 30) versus Routine Care (*n* = 30) Average of 6 sessions, 20–40 min, once or twice per week Intervention consists of psychoeducation regarding illness and treatment; MI exploring patient's perspective of illness; Cognitive behavioural strategies 60 SZ 48M/12F Inpatients Pre‐intervention and post intervention tests (no time‐frame specified)	Intervention 36.13 (8.9), Control 39.67 (10.6) Disease duration Intervention 12.23 (7.8), Control 16.75 (11.4)	Visual Analog Scale for Assessing Treatment Adherence Drug Attitude Inventory (DAI)	Significant differences were found in attitude and reported adherence for the experimental group, before and after. Significant differences were found between the experimental and control group in the degree of change in attitude and reported adherence. With respect to the relationship between attitude and reported adherence, the experimental group presents a positive correlation between attitudes and adherence before intervention (*r* = 0.51) and a positive correlation between attitudes and adherence after the intervention (*r* = 0.59). The control group also represents a correlation between attitude and adherence before the intervention (*r* = 0.52). The entire sample presents a positive correlation between change in attitude and change in reported adherence (*r* = 0.42). Measuring adherence, identifying the cause of non‐adherence in each particular patient and integrative one‐on‐one tailor‐made intervention to promote adherence is essential in making effective treatment decisions in daily clinical practice. Integrative interventions such as the one utilised in this study thus have an important place in the routine care of patients diagnosed with schizophrenia. They may contribute to shorter hospital stay and lower rates of hospitalisation by strengthening adherence to the drug regimen. These may, on a broader scale, result in a smoother functioning and more cost‐effective mental health system.
Dikec and Kutlu (2016)	Turkey Quasi‐experimental design	Adherence Therapy (AT) (*n* = 15) versus Controls (*n* = 15) AT comprised of 8 sessions, 30–50 min AT consists of CBT and MI techniques 30 SZ 25M/5F Outpatients Baseline and post‐test assessments, 3 and 6‐month follow‐ups	AT 39.93 (8.11), Controls 41.73 (6.41) Duration of disease (years) AT 16.13 (SD 9.09), Controls 18.27 (SD 9.21)	Medication Adherence Rating Scale (MARS)	Scores of the patients in the experimental and control groups between the baseline, post‐test, and 3 and 6‐month follow‐up revealed a significant difference only in the MARS post‐test scores. When the MARS scores of the patients in the experimental group were compared at each time point, significant differences were found between the following: The baseline and post‐test MARS scores The baseline and 3‐month follow‐up MARS scores The baseline and 6‐month follow‐up MARS scores The post‐test and 6‐month follow‐up MARS scores Results demonstrate that treatment adherence of patients with schizophrenia who have received AT will be higher than that of patients with schizophrenia who have not received AT. Periodical repetition of AT will help patients develop behavioural changes.
Gleeson et al. (2013)	Australia Single (assessor) blind RCT	Relapse prevention therapy (RPT) with individual and family interventions (FI) (*n* = 41) versus specialist FEP care alone (TAU) (*n* = 40) Average of 8.51 individual sessions in RPT, completed over 7 months, delivered fortnightly Individual sessions used CBT, MI, and psychoeducation methods to reduce risk of relapse FI for FEP was included, also using cognitive behavioural approaches and psychoeducation 81 FEP participants 51M/30F Outpatients Baseline assessment, 7‐ (end of therapy), 12‐, 18‐, 24‐, and 30‐ month follow‐up	20.1 (3.1) Mean (SD) DUP 384.8 (567.9)	Medication Adherence Rating Scale (MARS)	At 12‐month follow‐up, the relapse rate was significantly lower in the therapy condition compared with specialised treatment alone and time to relapse was significantly delayed for those in the relapse therapy condition. However, such differences were not maintained. There was a significant group by time interaction for the MARS. Analysis indicated that the RPT and not the TAU group had significant change over time. For the RPT group, there was significant improvement from baseline to 24 months and baseline to 30 months in terms of medication adherence. Endpoint analysis was not significant. Unexpectedly, psychosocial functioning deteriorated over time in the experimental but not in the control group Further research is required to ascertain if the initial treatment effect of the RPT can be sustained. Further research is needed to investigate if medication adherence contributes to negative outcomes in functioning in FEP patients who have reached remission, or, alternatively, if a component of RPT is detrimental.
Gray et al. (2006)	Europe (Netherlands, Germany, England, Italy) Single blind, multi‐centre RCT	Adherence Therapy (AT) (*n* = 204) versus Health Education (HE) (control) (*n* = 205) Eight weekly sessions, 30–50 min AT provided a brief individual cognitive behavioural approach, consisting of medication problem‐solving; history of medication; discussing beliefs relating to medication; exploring ambivalence; and future use 409 SZ participants 245M/164F Inpatients and outpatients Baseline assessment and 52 week follow‐up	41.5 (11.5) Mean (SD) psychiatric inpatient days in past year 27.9 (60.4)	Medication Adherence Questionnaire (MAQ)	There was no significant difference between AT and HE at follow‐up. An exploratory post hoc analysis examined the effect of AT in a subgroup of less adherent participants. Just under a third of the sample (*n* = 120) met this criterion. There was no significant difference in medication adherence between the groups at follow‐up. This study showed that AT had no clear benefit in terms of treatment adherence when compared with HE for people with generally chronic schizophrenia in general adult mental health services, who showed recent clinical instability.
Harmanci and Budak (2022)	Turkey RCT	Psychoeducation based on MI techniques (*n* = 75) versus controls (*n* = 75) 2 days per week for 6 weeks, 60 min, group sessions of 10–11 participants The intervention included discussion of stigma; side effects; future goals; expectations/benefits of treatment; side effects; exploration of ambivalence towards treatment; and thoughts about being healthy 150 SZ participants 81M/69F Outpatients Pre‐ and post‐test assessments (timeframe not specified)	Mean age not specified Age range 18–28 (*n* = 13) 29–39 (*n* = 57) 40–50 (*n* = 57) ≥ 51 (*n* = 23) Duration of disease (years) 0–5 (*n* = 15) 6–11 (*n* = 89) 12–17 (*n* = 46)	Morisky Medication Adherence Scale (MMAS)	There was no significant difference between the mean pre‐test medication adherence scores of the patients in the control and experimental groups. Scores of the experimental groups were significantly higher than the control group. Mean post‐test medication adherence scores in the experimental group were significantly higher in comparison to pre‐test mean scores. There was no difference between scores of the patients in the control group. Effective results will be obtained if routine psychosocial services, especially psychoeducation programs, are provided in community mental health centres based on MI techniques. MI seems to have great importance in controlling the important factors in the deterioration of psychological wellbeing, such as medication compliance.
Kızılırmak Tatu and Demir (2020)	Turkey Quasi‐experimental design	Group Psychoeducation (*n* = 21) versus Controls (*n* = 21) Eight sessions, 1 day per week, 60 min Sessions covered recognising SZ; treatment for SZ; coping with stress; communication skills; problem‐solving skills; relationships and social activities 42 SZ participants 26M/16F Outpatients Pre‐test, post‐test, and 3‐month follow‐up	Psychoeducation group 41.24 (9.78) Control group 40.52 (11.54) Treatment duration (years) 0–2 (*n* = 6) 2–5 (*n* = 6) 5–10 (*n* = 17) ≥ 10 (*n* = 13)	Medication Adherence Rating Scale (MARS)	There was a significant difference between the intervention group and the control group in terms of MARS pre‐test and post‐test follow‐up scores. The MARS final follow‐up test scores of the intervention group increased compared to the pre‐test scores, and the control group. Thus, there was a significant time by group interaction on MARS scores. Group psychoeducation that focuses on social skills development should be integrated into routine functioning in order to improve the adherence to treatment, quality of life and well‐being of schizophrenic patients followedby CMHC.
Kopelowicz et al. (2012)	USA Multi‐site RCT	Multifamily Group (MFG‐A) Therapy for medication adherence (*n* = 64) versus MFG standard (MFG‐S) (*n* = 53) versus TAU (*n* = 57) 3 initial joining sessions, a 1‐day workshop, and 24 bimonthly sessions over 12 months MFG‐S included psychoeducation covering SZ and treatment; problem‐solving skills; open discussion of personal experiences MFG‐A is culturally adapted, including similar psychoeducation topics. It includes analysing specific obstacles to medication adherence, embedded within their sociocultural context. Other patients with similar experiences were invited to facilitate changes in medication beliefs among participants 174 Mexican American SZ participants MFG‐A 67% M/33% F MFG‐S 68% M/32% F TAU 61% M/39% F Inpatients and outpatients Baseline assessment and follow up data at 4, 8, 12, 18, and 24‐months	MFG‐A 32.6 (11.3), MFG‐S 29.6 (10.8), TAU 32.8 (12.6) Mean (SD) lifetime hospitalisations MFG‐A 5.5 (5.2) MFG‐S 5.6 (6.3) TAU 7.1 (6.3)	Treatment compliance interview Psychiatrist rating on the likelihood of medication compliance Los Angeles County mental health department management information system (MIS)	More participants in MFG‐A were fully adherent than those in TAU at all assessments after 4 months, and MFG‐A was significantly better than MFG‐S at 8 and 18 months, but not at 24 months. At the end of the 1‐year treatment, MFG‐A was associated with higher medication adherence than MFG‐S or TAU. There was no significant difference at any point between MFG‐S and TAU groups. The MFG‐A participants had a longer time to first hospitalisation and were less likely to be hospitalised than those in MFG‐S and TAU. Increased adherence accounted for one‐third of the overall effect of treatment on the reduced risk for psychiatric hospitalisation. This study points out the value of addressing adherence within the context of family treatment but also suggests that other salutary aspects of MFG (e.g., active engagement of patients and families in treatment, communication skills training, problem‐solving approach, and social network development) may have contributed to decreased attrition and reduction in hospitalizations. The relative loss of efficacy over time indicates the possible need for booster sessions to sustain the benefits of the intervention. Clinicians working with this population might incorporate a culturally adapted, adherence‐focused MFG treatment to improve the course and outcome of schizophrenia as a component of clinical care. Moreover, given the individualised process of the trial's cultural adaptation, it may prove useful in psychosocial interventions for schizophrenia spectrum disorders applied to communities from a wide range of sociocultural backgrounds.
Lambert, Bock, et al. (2010); Lambert, Conus, et al. (2010)	Germany Catchment area comparison design (non‐randomised)	Assertive Community Treatment (ACT) (*n* = 64) versus Standard Care (SC) (*n* = 56) over 12 months ACT provided intensive, need‐adapted psychotherapy (therapists trained to provide CBT, dynamic, and family psychotherapy) 120 participants 68M/52F 49 FEP 71 Multiple‐episode patients Inpatients and outpatients Baseline assessment with 4, 12, 26, 38, and 52 weeks follow‐ups	ACT 31.4 (9.9) SC 37.6 (11.7) Median DUP (weeks) ACT 21.9 SC 27.6	Satisfaction with Antipsychotic Medication Scale (SWAM)	SC patients were about 5 times more likely to become nonadherent with medication throughout the study period the ACT group were more likely to be adherent with medication (OR, 3.5). Compared to SC, intensive therapeutic ACT as part of integrated care could improve 1‐year outcome. Future studies need to address in which settings these improvements can be sustained.
Maneesakorn et al. (2007)	Thailand Single‐blind RCT	Adherence Therapy (AT) (*n* = 16) versus TAU (*n* = 16) 8 one‐to‐one sessions, 15–60 min over 8 weeks AT is a brief CBT approach, incorporating MI techniques into the intervention Patient beliefs surrounding medication, side effects experienced, problems with medication, and ambivalence are explored. 32 SZ participants 23M/9F Inpatients Baseline assessment and 9 weeks follow‐up	AT 38.7 (12.784) TAU 43 (6.516) Mean (SD) duration of illness (years) AT 9.64 (6.89) TAU 9.25 (6.21)	Drug Attitude Inventory (DAI) Satisfaction With Antipsychotic Medication scale (SWAM)	At nine‐week follow up, statistically significant improvement was found in the AT group compared with the TAU group in attitude towards medication and satisfaction with medication. AT has a positive impact on patients' psychiatric symptoms, attitude towards and satisfaction with medication.
Marchira et al. (2019)	Indonesia RCT	Brief Interactive Psychoeducation for Caregivers (*n* = 50) versus Controls (*n* = 50) 4 weekly individual sessions lasting 1–2 h The intervention was culturally adapted, including information on psychotic disorders, signs, symptoms, treatment, support systems, available services, and signs of exacerbation or relapse 100 EP patients and their caregivers 61M/39F (patients) 35M/65F (caregivers) Outpatients (inferred) Pre‐intervention assessment, 1 and 6 month follow‐ups	Patients 22.4 (4.5) Caregivers 47.2 (12.17)	Compliance and Relapse Assessment (CRA) Drug schedule cards (completed by caregiver)	Analyses showed that the intervention group had significantly better adherence to pharmacotherapy regimens. There was no statistically significant difference in relapse/rehospitalization rate at 6 months between control and intervention groups, although 18% of patients in the control group were re‐hospitalised compared with 6% of patients in the intervention group. A brief investigation into non‐adherence among participants in the study revealed that the reasons were economic reasons, feeling that the patient had recovered or getting better, fear of side effects of the medicine of dependency and poor insight of the patient. Brief psychoeducation of caregivers of the early phase psychotic patients is feasible in a low resource setting, using culturally adapted modules. Incorporating the module in routine practice, particularly to increase adherence to medication is particularly important because poor adherence to medication, particularly due to lack of knowledge regarding the nature of psychosis, will increase the risk of relapse.
Ngoc et al. (2016)	Vietnam Single (assessor) blind RCT	Family Schizophrenia Psychoeducation Program (FSPP) (*n* = 30) versus TAU (*n* 29) 3 sessions, 1.5 h FSPP is culturally modified for family behaviours in Vietnam Sessions covered SZ; its causes; treatments; potential prognoses; stigma; family support; problem‐solving; helping patients reintegrate into the community 59 recent onset schizophrenia patients 48.6% M/51.4% F (FSPP) 51.4% M/48.6% F (controls) Inpatients Baseline assessment and 6 month follow‐up	FSPP 24.87 (5.11) Controls 23.69 (4.37) Mean (SD) duration of SZ FSPP 1.58 (1.13) Controls 1.99 (1.06)	Medication Compliance Inventory (adapted for Vietnam) Quality of Life Enjoyment and Satisfaction Questionnaire Stigma towards Schizophrenia Scale (developed for Vietnamese patients) Consumer Satisfaction Scale	There were significant treatment effects on: quality of life, stigma, medication compliance (*R* ^2^ = 0.14), and consumer satisfaction, with all effects favouring the treatment group. The FSPP adapted for Vietnam appears to reduce stigma, and improve quality of life and medication compliance of Vietnamese patients with schizophrenia. It involves relatively few resources and it may be useful for it or equivalent programs to be implemented in other hospitals and other low‐income Asian countries.
O'Donnell et al. (2003)	Ireland Single (assessor) blind RCT	Compliance Therapy (*n* = 28) versus Non‐specific Counselling (Controls) (*n* = 28) 5 sessions, 30–60 min CT is a CBT intervention using MI techniques and psychoeducation Sessions cover patient history; understanding of illness; ambivalence to treatment; maintenance medication; and stigma 56 SZ participants (including 12 FES patients) 41M/15F Inpatients Baseline assessment and 1 year follow‐up	32 (9) Mean (SD) duration of illness (years) Compliance therapy 6 (7) Controls 4 (5)	Clinician, health professional, and family members' assessment of compliance Drug Attitude Inventory (DAI)	Compliance therapy did not confer a major advantage over non‐specific therapy in improving compliance at 1 year or in any of the secondary outcome measures. Baseline compliance (OR = 29.59) and baseline attitudes to treatment (OR = 1.36) were predictors of compliance at 1 year follow up. Undergoing compliance therapy was not a predictor of compliance at 1 year. Carer attendance at an education programme was a predictor of compliance at 1 year. Compliance therapy may not be of benefit to patients with schizophrenia. Attitudes to treatment at baseline predicted adherence 1 year later and may be a clinically useful tool.
Oneib et al. (2025)	Morocco Prospective, single‐arm trial	Psychoeducation Program culturally adapted 10 weekly sessions, 1–2 h, delivered individually or in groups The intervention aims to improve illness understanding, strengthen the therapeutic alliance, and promote medication adherence through education about treatment and living with SZ 100 SZ patients 86M/14F Outpatients Pre‐intervention assessment and 6 month follow‐up	37.73 (10.518) Mean (SD) duration of illness (years) 14.22 (9.73)	Medication Adherence Report Scale, short form (MARS‐5)	Before psychoeducation, 91% of patients had poor adherence, with a mean score (MARS‐5) of 10.81 ± 5.66. After psychoeducation, 63.7% of patients had good adherence, with a mean score of 20.49 ± 4.81, showing a significant improvement. Psychoeducation programs should be systematically offered to patients with schizophrenia and their families, particularly in the early stages of the illness, within a recovery‐focused framework. This approach aims to empower individuals with schizophrenia and their families, significantly reduce relapse rates by improving treatment adherence, enhance knowledge acquisition, and help rebuild the identity disrupted by the illness, thereby improving psychosocial autonomy and the overall quality of life for these patients.
Pitschel‐Walz et al. (2006)	Germany Multicentre, single blind RCT	Psychoeducation Program (*n* = 125) versus Routine Care (controls) (*n* = 111) 4 weekly sessions and 4 monthly sessions, 1 h The intervention provided information on symptoms, treatment, relapse prevention, coping strategies, and open discussion for problems pertaining to the illness 236 SZ and SAD patients Inpatients Baseline analysis, follow‐up at 12 and 24 months (compliance documented at monthly appointments)	33 (SD not specified) Mean duration of illness (years) 7	Clinician‐assessed compliance Plasma drug level concentrations	Patients who attended psychoeducational groups showed significantly better compliance than patients under routine care without psychoeducation: Very good/good compliance after 12 months: psychoeducation (80%), controls (58%) Very good/good compliance after 24 months: psychoeducation (80%), controls (55%) It was possible to significantly reduce the rehospitalization rate after 12 and 24 months in patients who attended psychoeducational groups compared with those receiving routine care. A relatively brief intervention of 8 psychoeducational sessions with systematic family involvement in simultaneous groups can considerably improve the treatment of schizophrenia. Psychoeducation should be routinely offered to all patients with schizophrenia and their families.
Randall et al. (2017)	Canada Retrospective cohort design	Assertive Community Treatment (ACT) (*n* = 244) versus Controls (*n* = 449) Treatment period of 24 months (Mean duration of treatment = 510.4 days) The intervention program emphasises psychotherapy and medication adherence, including group therapy with families. ACT addresses stigma and promotes community psychoeducation. 693 FEP patients 493M/200F Outpatients Analyses performed for the treatment period and during post‐treatment (two‐year period following discharge)	EPPIS 18.8 (2.48) Controls 19.9 (3.66)	Drug Programs Information Network (DPIN) database	During treatment, those in the program were significantly more likely to have adhered to medication (OR = 4.71). These effects diminished, but were still significant during the post‐treatment period. This study provides support for assertive treatment services targeting FEP patients. Being able to demonstrate that patients in the program are using medications at an increased rate is an indication that the program is effective. The finding that ACT increased the use of antipsychotics after program discharge is significant and indicates that these programs can alter long term adherence to medications.
Robinson et al. (2018)	USA Multi‐site RCT	NAVIGATE program (*n* = 223) versus Community Care (*n* = 181) 2 year treatment duration NAVIGATE provides psychoeducation, resilience‐focused individual therapy, and supported employment/education, with modules including information on medication and health‐promoting behaviour 404 FEP patients 73% M/27% F Outpatients Assessments at baseline, 3, 6, 12, 18, and 24 months	23 (No SD specified) Mean (SD) DUP (weeks) NAVIGATE 178.91 (248.73) Community care 211.43 (277.49)	Adherence Estimator scale	Scores on the Adherence Estimator scale did not differ between groups at baseline, and they decreased (indicating fewer beliefs associated with nonadherence) significantly among NAVIGATE but not community care participants The Adherence Estimator scale data documented an advantage with NAVIGATE but not community care treatment for medication beliefs related to adherence. An important future research question is whether these belief changes translate into improved adherence.
Schulz et al. (2013)	Germany and Switzerland Multicentre Single blind RCT	Adherence Therapy (AT) (*n* = 80) versus TAU (*n* = 57) 8 one‐to‐one sessions AT uses CBT and MI techniques to explore patients' beliefs and attitudes towards treatment; assess issues/side effects relating to medication; review history of medication/illness experiences; explore ambivalence towards medication and the role of medication in future goals 137 SZ patients 80M/57F Inpatients Baseline assessment and 12 weeks post‐discharge follow‐up	AT 35 (10) TAU 36 (9)	Drug Attitude Inventory (DAI) Medication Adherence Rating Scale (MARS) Blood serum concentration	Compared to TAU, AT significantly improved symptoms. There were no significant differences in adherence, beliefs about treatment or global functioning between AT or TAU. The findings from this trial would lend weight to the argument that intervening as soon as possible after an acute episode of illness in patients with schizophrenia affords the best chance to affect change. One of the most important challenges in adherence research is the recruitment of patients who are treatment non‐compliant. Different methodological approaches, other than standard RCT, will need to be considered in future trials of AT.
Skarsholm et al. (2014)	Denmark Pragmatic controlled trial	System‐Oriented Therapy (SOT) (*n* = 30) versus Individual Compliance Therapy (CT) (*n* = 40) SOT based on five standards of information on AP treatment; solving compliance problems; reminder systems; medication reconciliation; guidelines for AP treatment (structure of intervention delivery not specified) CT based on CBT approaches, principles of MI and concordance skills. 6 sessions and 3 booster sessions, 30–45 min 70 SZ/SAD patients 31M/39F Inpatients Baseline assessment and 6 months follow‐up	SOT 40.1 (46.7) CT 43.4 (10.6) Mean duration of illness (years) SOT 11 CT 19	Patient‐assessed compliance Drug Attitude Inventory (DAI) Appointment monitoring PANSS‐item G12 on judgement about illness and need for treatment	There was a significantly greater proportion of the individual CT group compared to the system‐oriented group that did not complete follow up (15/40 versus 4/30). In the CT group, each participant received on average 3 sessions (SD 2.1) and 0 booster sessions (SD 0.7). Researchers found a difference in both the compliance scale and PANSS favouring the SOT, although it did not reach statistical significance. This study suggests that compliance problems are best solved by a multifactorial intervention on the system level. Yet the point that the system‐oriented intervention was superior to the individual intervention can be questioned. The system‐oriented intervention is likely suitable to be applied to compliance problems in other groups of vulnerable patients with chronic diseases. This study may be considered a first step in investigating the potential effect of system‐based interventions on improving patient compliance. Although not all the estimated effects reached significance, they all favoured the systemic intervention and they should therefore serve as the basis of larger and more definitive studies of this kind.
Staring et al. (2010)	Netherlands Single (assessor) blind RCT	Treatment Adherence Therapy (TAT) (*n* = 54) versus TAU (*n* = 55) TAT consists of selected modules responding to individual determinants of nonadherence through MI, medication optimisation, and behavioural training Duration and number of sessions varied based on individual needs, with a maximum time of 6 months 109 participants 76 SZ, 33 SAD 77M/32F Outpatients Baseline (T0) and end of treatment assessment (T1), with 6 month follow‐up (T2)	39 (11.6)	Compiled measure of adherence using the Service Engagement Scale (SES) Semi‐structured interviews	There were significant differences in service engagement (*d* = 0.48) and medication adherence (*d* = 0.41) between the two treatment conditions at the end of the TAT intervention (T1). Six months later (T2), only medication adherence was still statistically significant (*d* = 0.3). Near‐significant effects were found regarding involuntary readmissions. People who refused to participate in the study were engaging less with services than those included. Interventions such as TAT may not be acceptable for people with very low treatment adherence. These may benefit more from assertive treatments and direct incentives to motivate them. An important contributor to the effects may be that, after a person's individual situation has been assessed, TAT provides various intervention modules.
Tessier et al. (2023)	France Single‐blind RCT	Family Psychoeducation (FP) (*n* = 12) versus TAU (*n* = 13) 6 group sessions over 1.5 months, 1.5 h. Booster session offered 6 months afterwards FP focuses on understanding SZ, drug treatments, and modes of hospitalisation. It also provides information on managing patients' crisis states, and caregiver experiences. 25 SZ/SAD patient and caregiver dyads 19M/6F (patients) 4M/21F (caregivers) Outpatients Baseline assessment, 3, 6, and 12 month follow‐ups	Patients 33.3 (9.7) Caregivers 50.6 (14) Mean (SD) duration of illness (years) 7.5 (7.1)	Medication Adherence Rating Scale (MARS) Computerised medical records Compliance Rating Scale (CRS)	A lower rate of relapse was observed at 3, 6 and 12 months for patients whose caregivers participated in the intervention group. The difference was significant at 12 months. Medication adherence assessed by CRS estimated by caregivers was not modified by the intervention. Medication adherence, which is recognised as complex and multi‐determined phenomenon, cannot be resolved by a single, non‐specific intervention.
Uzenoff et al. (2008)	USA Single (assessor) blind RCT	Adherence Coping Education (ACE) therapy versus Supportive Therapy (ST) 14 sessions (6 weekly, 8 biweekly), 30–45 min over 6 months ACE includes CBT strategies and MI, which aims to establish a therapeutic alliance, promote treatment adherence, develop a plan for maintenance treatment, and rehabilitation. It also explores patient attitudes towards medication and the illness. 24 FEP 60% M/40% F Inpatients (enrolled and assessed after discharge) and outpatients Baseline, mid‐treatment (3 months), and post‐treatment (6 months) assessment	Age not specified (minimum 16 years‐old)	Patient‐reported medication adherence Rating Of Medication Influences (ROMI) Insight and Treatment Attitudes Questionnaire (ITAQ)	On the measure of medication adherence, there was a ceiling effect wherein 100% of participants endorsed the highest level of adherence at both mid‐ and posttreatment. Significant within‐group improvements from baseline to posttreatment were found for ACE participants on Benefit of Medication (*d* = 0.59). No significant within‐group changes were found for the ST group. Significantly greater improvement in treatment attitudes was observed for participants in ACE compared with ST at mid‐treatment. No other statistically significant interactions were obtained. The findings suggest that ACE therapy is feasible and that it may improve treatment attitudes in individuals recovering from an initial psychotic episode. The long‐term stability of these findings needs to be evaluated to better characterise these attitude changes. The findings show promise for the development of psychotherapeutic approaches to addressing treatment nonadherence, and support the role of cognitive behavioural interventions as effective adjunctive treatment for early psychosis.
Valencia et al. (2012)	Mexico Single (assessor) blind RCT	Integrated Treatment Approach (ITA) (*n* = 39) versus Medication Alone (Control) (*n* = 34) ITA is comprised of family therapy, psychoeducation, CBT (not explicitly stated) informed goals relating to medication compliance, knowledge about illness, learning warning signs of relapse, problem‐solving, and social skills Sessions and structure of intervention not specified 73 FEP 55M/18F Outpatients Baseline and end of treatment (12 months) assessment	ITA 24.5 (3) Control 24.1 (3.2)	Clinician rated adherence during monthly consultations (percentage of medication taken) Positive And Negative Syndrome Scale (PANSS) Relapse and rehospitalisation rates	At the end of treatment, lower relapse (10.3%) and rehospitalization rates (5.1%) were found in the experimental group compared to controls (35.7% and 10.7%, respectively). Compliance with antipsychotic medication was significantly higher in the experimental group compared to controls (85% vs. 67.6%). These patients learned the necessary skills about symptom and medication management that helped prevent relapse. These results indicate that outcome can be improved through early intervention after the onset of psychosis.
Zhu et al. (2021)	China Single‐blind RCT	Compensatory Cognitive Training (CCT) (*n* = 24) versus CCT plus Medication Self‐management Skills Training (MSST) (*n* = 26) versus TAU (*n* = 22) CCT—8 group sessions over 4 weeks, 2 h. Focuses on improving attention, memory, problem‐solving, and planning skills MSST—4 group sessions over 4 weeks, 2 h. Focuses on psychoeducation concerning medication, its use, effects, and communicating issues with physicians. 72 SZ patients 37M/35F Inpatients Baseline assessment and 3 months follow‐up	CCT 30 (25–39) CCT + MSST 32 (26–41) TAU 33 (26–41) Course of disease (years) CCT 10 (3–18) CCT + MSST 11 (4–15) TAU 7 (3–14)	Medication Adherence Questionnaire (MAQ)	Compared with the TAU group, the CCT group had significant improvements in verbal fluency, total cognitive function and medication adherence, and the CCT + MSST group had significant improvements in verbal fluency, total cognitive function, positive symptoms, and medication adherence. Compared with the CCT group, the CCT + MSST group had significant improvements in total cognitive function. Follow‐up only for 3 months may not fully reveal the effects of CCT + MSST. Combined intervention may play an important role in preventing patients from stopping or reducing antipsychotics treatments owing to lack of knowledge of antipsychotic drugs, which is not available in CCT.

Abbreviations: CBT, cognitive behavioural therapy; DUP, duration of untreated psychosis; EP, early psychosis; F, female; F, positive and negative syndrome scale; FEP, first episode psychosis; FES, first episode schizophrenia; M, male; RCT, randomised controlled trial; SAD, schizoaffective disorder; SD, standard deviation; SZ, schizophrenia; TAU, treatment as usual.

### 3.1 Risk of Bias and Certainty Assessment

Quantitative studies were assessed using the EPHPP tool, which evaluates six domains: selection bias, study design, confounders, blinding, data collection methods and withdrawals/dropouts. Each domain was rated as strong, moderate, or weak, and these ratings were combined according to the EPHPP guidelines to provide an overall global rating: strong (no weak domains), moderate (one weak domain), or weak (two or more weak domains). Based on the EPHPP tool (see Table 1), quantitative medication non‐adherence studies were rated as: ‘strong’ (*n* = 4), ‘moderate’ (*n* = 10) and ‘weak’ (*n* = 4). Using the JBI tool (see Table 3), qualitative medication non‐adherence studies were rated as: ‘strong’ (*n* = 24) and ‘moderate’ (*n* = 1), suggesting a predominance of low‐bias research. Moreover, according to the EPHPP tool, the global quality ratings for the studies examining psychosocial interventions were rated as: ‘strong’ (*n* = 19), ‘moderate’ (*n* = 11) and ‘weak’ (*n* = 4) (see Table 2). This indicates that most studies included in the present review were at low risk of bias.

### 3.2 Reporting Bias Mitigation

To reduce the risk of reporting bias, we applied several strategies. First, our search strategy included multiple databases and comprehensive keywords to capture all relevant studies, regardless of publication date or design. Second, we limited inclusion to peer‐reviewed studies to ensure methodological rigour and consistent reporting standards. Third, we assessed study‐level risk of bias using validated tools (EPHPP for quantitative studies and JBI for qualitative studies), and these ratings were incorporated into the synthesis to highlight findings from higher‐quality studies. Together, these steps help mitigate selective outcome reporting and enhance confidence in the review findings.

### 3.3 Synthesis Method

We synthesised the data using a thematic narrative approach, integrating findings across quantitative and qualitative studies. Quantitative findings on medication non‐adherence and psychosocial interventions were summarised descriptively, with consideration of study quality. Qualitative data were analysed thematically according to the main reasons, attitudes, and beliefs regarding medication non‐adherence, and findings were mapped onto the core themes. Intervention studies were summarised with attention to strategies and outcomes, and the synthesis of qualitative and quantitative evidence was presented in tables and narrative form to ensure clarity and facilitate translation into clinical practice.

## 4 Medication Non‐Adherence

### 4.1 Poor Insight

Four studies reported significant associations between poor insight and medication non‐adherence in FEP (Mutsatsa et al. 2003; Perkins et al. 2006; Quach et al. 2009; Sapra et al. 2014), whereas one study found no significant association (So et al. 2024). The lack of significance in the latter may reflect the use of an abbreviated version of the Scale to Assess Unawareness of Mental Disorder (SUMD), which may not capture the full scope of insight (So et al. 2024). Additionally, four qualitative studies identified treatment refusal among clients who denied experiencing psychosis (Chua et al. 2024; Hon 2012; Intharit et al. 2021; Yeisen et al. 2017). Collectively, these quantitative and qualitative findings indicate that poor insight can hinder treatment adherence in FEP.

### 4.2 Low Perceived Need for Medication

Quantitative evidence indicates that 8.8% of FEP clients consider medication unnecessary, compared to 31.3% of multi‐episode clients, suggesting that low perceived need is less common but present early in the illness (Sapra et al. 2014). Poor adherence is associated with more negative attitudes towards medication (Mutsatsa et al. 2003) and often occurs when symptoms are perceived as mild or non‐disruptive (Bjornestad et al. 2017; Brown et al. 2013; Chua et al. 2024; Cowan et al. 2020; Intharit et al. 2021; Vaingankar et al. 2020). Clients may also weigh the perceived benefits against costs, such as side effects, which can reduce the perceived necessity of medication (Cowan et al. 2020). These findings suggest that low perceived need stems from clients' beliefs that their symptoms are mild and that the burden of medication exceeds its perceived value.

### 4.3 Preference for Self‐Management

Four qualitative studies found that some FEP clients preferred to manage their psychosis symptoms through willpower rather than medication (Bjornestad et al. 2017; Chua et al. 2024; Eisner et al. 2014; Hui et al. 2018). Non‐adherence beyond the acute phase occurred when clients were uncertain whether recovery was due to treatment or their own efforts (Bjornestad et al. 2017). These findings suggest that medication non‐adherence may, in part, reflect a desire to maintain personal control over recovery.

### 4.4 Resistance to Medication Control

Qualitative studies indicate that some individuals resist medication due to fears of being controlled by treatment (Artaud et al. 2020; Chua et al. 2024). Clients also expressed concerns that medication could compromise their sense of identity (Artaud et al. 2020; Chua et al. 2024). Non‐adherence often emerged after discharge, reflecting discomfort with the central role of medication in their treatment (Gray and Deane 2016; Perry et al. 2007; Yeisen et al. 2017). These findings suggest that concerns about autonomy, including fears of being controlled by treatment or compromising one's sense of identity, can contribute to medication non‐adherence in FEP.

### 4.5 Mistrust of Medication and Mental Health Services

Five qualitative studies identified mistrust regarding the safety, efficacy, or intentions of treatment and healthcare providers as a factor influencing non‐adherence (Artaud et al. 2020; Chua et al. 2024; Gray and Deane 2016; Islam et al. 2015; Niemeyer et al. 2018). One study identified mistrust in the clinician as a primary barrier to treatment engagement (Polillo et al. 2022), while another found that covert non‐adherence was not disclosed to the treatment team, partly due to fear of hospitalisation (Artaud et al. 2020). Mistrust was further influenced by clients' symptoms, particularly suspiciousness, or by perceptions that medication information was insufficiently provided (Chua et al. 2024; van der Heijden‐Hobus et al. 2024). These findings suggest that mistrust can contribute to medication non‐adherence in FEP, arising both from psychosis‐related suspiciousness and from challenges in the client–clinician relationship.

### 4.6 Religious, Spiritual and Cultural Beliefs

Qualitative studies identified holding spiritual or religious conceptualisations of one's psychosis, as well as favouring alternative healing practices aligned with these beliefs, as a factor influencing medication non‐adherence in FEP (Artaud et al. 2020; Chua et al. 2024; Penny et al. 2009; Islam et al. 2015; Vaingankar et al. 2020). In some cases, medication was perceived as prohibited by religious beliefs (Islam et al. 2015; Miller and McCormack 2006). Cultural norms could also be experienced as restrictive, limiting open discussion of mental health difficulties (Rathod et al. 2023). These findings suggest that clients' religious, spiritual, and cultural beliefs can strongly influence medication adherence in FEP.

### 4.7 Self‐Medication With Substances

Five qualitative studies identified substance misuse in FEP as a coping strategy for managing psychosis symptoms and related distress (Archie et al. 2013; Baker et al. 2015; Intharit et al. 2021; Lobbana et al. 2010; Sheridan Rains et al. 2024; Tan et al. 2019). Two studies reported that approximately 25% of FEP clients used cannabis to alleviate positive symptoms or the side effects of antipsychotic medications (Kolliakou et al. 2015; Mané et al. 2015). FEP patients also reported using street drugs and alcohol to manage stress, distract from negative thoughts and reduce distress and anxiety (Archie et al. 2013; Tan et al. 2019). These findings suggest that substance use as a form of self‐medication may reduce perceived need for prescribed medication, potentially contributing to medication non‐adherence in FEP.

### 4.8 Previous Negative Experiences With Medication and Healthcare

Seven qualitative studies reported that past adverse experiences with medication or healthcare providers undermined adherence and trust in FEP (Brown et al. 2013; Chua et al. 2024; Cowan et al. 2020; Perry et al. 2007; Rathod et al. 2023; Stewart 2013; Yeisen et al. 2017). Negative experiences included feelings of powerlessness during admission, inhumane treatment, and coercive practices such as forcible injections, which reinforced perceptions of medication as a symbol of control (Polillo et al. 2022; Perry et al. 2007; Stewart 2013). Other factors affecting adherence included insufficient information about treatment duration and side effects, limited collaboration in decision‐making, and side effects that disrupted daily functioning (Chua et al. 2024; Hickling et al. 2018; Cowan et al. 2020; van der Heijden‐Hobus et al. 2024; Yeisen et al. 2017). These findings highlight how previous negative experiences with medication and healthcare can influence medication non‐adherence in FEP, including factors such as perceived coercion, limited information, and disrupted daily functioning due to medication side effects.

### 4.9 Fear or Intolerance of Medication Side Effects

Five qualitative studies found that non‐adherence often arose from concerns about, or intolerance to, side effects, frequently leading to medication discontinuation (Artaud et al. 2020; Bjornestad et al. 2017; Chua et al. 2024; Dijkstra et al. 2024; Hon 2012). Four additional studies identified side effects as a primary reason for non‐adherence in FEP (Endale Gurmu et al. 2014; Polillo et al. 2022; Sapra et al. 2014; Stürup et al. 2023). Common concerns included weight gain, sedation, cognitive fatigue and reduced mental capacity (Artaud et al. 2020; Bjornestad et al. 2017; Brown et al. 2013; Dijkstra et al. 2024). After the acute phase, some FEP clients reported that residual symptoms and relapse risk were more tolerable than the side effects themselves (Bjornestad et al. 2017; Dijkstra et al. 2024). These findings highlight that medication side effects are a key barrier to adherence in FEP, sometimes outweighing concerns about residual symptoms or relapse.

### 4.10 Perceived Ineffectiveness of Medication

Four qualitative studies reported that non‐adherence and reduced motivation to continue treatment were common among FEP clients who perceived antipsychotic medications as ineffective in alleviating symptoms (Chua et al. 2024; Hui et al. 2018; Intharit et al. 2021; Yeisen et al. 2017). Some clients questioned the long‐term efficacy of their medications (Hui et al. 2018), while others reported that experiencing side effects without symptom relief further discouraged adherence (Yeisen et al. 2017). These findings suggest that perceptions of limited or absent symptom improvement can contribute to medication non‐adherence in FEP.

### 4.11 Observed Negative Treatment Experiences

Two qualitative studies found that witnessing other patients experience side effects or distress during admission created frightening and distressing impressions regarding antipsychotic medication for FEP patients (Chua et al. 2024; Yeisen et al. 2017). Additionally, exposure to unpredictable or aggressive behaviour from chronically unwell inpatients further heightened clients' anxiety and reinforced a desire to view themselves as distinct from these patients (Perry et al. 2007; Stewart 2013). These findings suggest that witnessing negative experiences in others can contribute to medication non‐adherence in FEP.

### 4.12 Complexity of Medication Regimen

Two qualitative studies found that medication non‐adherence was more likely among FEP clients who found the daily dose or scheduling of their medication confusing (Cowan et al. 2020; Gray and Deane 2016). These findings suggest that complex medication regimens can contribute to medication non‐adherence in FEP.

### 4.13 Forgetfulness and Disorganisation

Five studies identified forgetfulness and disorganisation as barriers to consistent medication use and appointment attendance in FEP (Eisner et al. 2014; Endale Gurmu et al. 2014; Hui et al. 2006; Intharit et al. 2021; Polillo et al. 2022). These findings indicate that forgetfulness and disorganisation are common contributors to medication non‐adherence in FEP.

### 4.14 Negative Family Attitudes Towards Mental Illness and Treatment

Five qualitative studies reported that adherence can be undermined by family members holding stigmatising views or negative beliefs about mental illness and psychiatric treatment (Chua et al. 2024; Intharit et al. 2021; Niemeyer et al. 2018; Penny et al. 2009; Rathod et al. 2023). Non‐adherence was observed when families encouraged discontinuation after clients felt better, perceived medication as ineffective, or found side effects unacceptable (Chua et al. 2024; Miller and McCormack 2006; Penny et al. 2009). Other contributing factors included family embarrassment, denial of the client's condition, and limited support, with some relatives withholding information or failing to encourage recovery (Intharit et al. 2021; Niemeyer et al. 2018). These findings suggest that negative or stigmatising family attitudes, including limited support or misunderstanding of mental illness, can contribute to medication non‐adherence in FEP.

### 4.15 Stigma

Internalised stigma has been shown to contribute to medication non‐adherence in FEP (Artaud et al. 2020; Chua et al. 2024; Cowan et al. 2020; Dijkstra et al. 2024; Hui et al. 2006; Vaingankar et al. 2020), with higher stigma levels associated with poorer adherence (Sapra et al. 2014; So et al. 2024). Antipsychotics were perceived as taboo and as reminders of illness, leading to lower self‐esteem, pessimism, and a desire to appear ‘normal’ (Artaud et al. 2020; Chua et al. 2024; Vaingankar et al. 2020; Perry et al. 2007). Anticipated stigma in social and occupational contexts further reinforced reluctance to continue with medication (Bjornestad et al. 2017; Chua et al. 2024). These findings suggest that internalised and anticipated stigma, including concerns about discrimination and threats to self‐image, can contribute to medication non‐adherence in FEP.

### 4.16 Core Beliefs

Seven qualitative studies found that negative self‐beliefs contributed to medication non‐adherence in FEP (Artaud et al. 2020; Bjornestad et al. 2017; Chua et al. 2024; Cowan et al. 2020; Dijkstra et al. 2024; Perry et al. 2007; Vaingankar et al. 2020). Patients described viewing medication use as a sign of weakness, failure, and/or inferiority, or feared that others would judge them negatively for needing treatment. These beliefs were reinforced by concerns about being permanently labelled a psychiatric patient and by experiences that evoked feelings of vulnerability or low self‐worth, particularly during inpatient care. Overall, these findings suggest that negative core beliefs pertaining to weakness, failure, and/or inferiority can contribute to medication non‐adherence in FEP.

### 4.17 Interpersonal Barriers to Medication Adherence

Three qualitative studies found that living alone or lacking supportive individuals can undermine medication adherence and increase relapse risk in FEP (Artaud et al. 2020; Intharit et al. 2021; Hui et al. 2018). Conversely, medication non‐adherence may also result from friends, family, or colleagues who encourage discontinuation, often believing the client has recovered or underestimating the importance of ongoing treatment, which can negatively impact self‐esteem (Vaingankar et al. 2020). These findings suggest that interpersonal influences can affect adherence in FEP, with lack of support or misinformed encouragement to stop medication contributing to medication non‐adherence.

### 4.18 Medication Paradox

Six studies reported that medication non‐adherence can occur when FEP patients experience symptom improvement after starting treatment, leading them to believe medication is no longer necessary (Chua et al. 2024; Cowan et al. 2020; Endale Gurmu et al. 2014; Hui et al. 2018; Intharit et al. 2021; Stürup et al. 2023). Following an acute episode, patients often wished to move on with their lives and distance themselves from both their treatment and illness (Chua et al. 2024; Perry et al. 2007). Medication non‐adherence was also reported when patients forgot that their improved symptoms were a direct result of medication (Gray and Deane 2016). Early medication discontinuation therefore appears partly driven by patients underestimating the role of medication in maintaining symptom improvement.

### 4.19 Enjoyment of Psychosis Symptoms/Does Not Want to Get Rid of Symptoms

Two qualitative studies indicated that some FEP patients may resist medication because they value certain aspects of their psychosis experience and do not want to get rid of their symptoms (Dijkstra et al. 2024; Hon 2012). Patients described grief or loss when medication reduced experiences they found pleasurable or meaningful during their psychosis, such as euphoria, heightened confidence, or a sense of special significance (Dijkstra et al. 2024; Hon 2012). Grandiose beliefs, such as feeling uniquely important, possessing special abilities, or understanding others' thoughts, were described as empowering (Hon 2012). For some, the attenuation of these experiences through antipsychotic medication was identified as contributing to a perceived loss of identity or sense of reality (Dijkstra et al. 2024). These findings suggest that medication adherence may be undermined when psychosis symptoms are perceived as beneficial or desirable.

### 4.20 Summary of Medication Non‐Adherence Reasons in FEP

**FIGURE 2:**
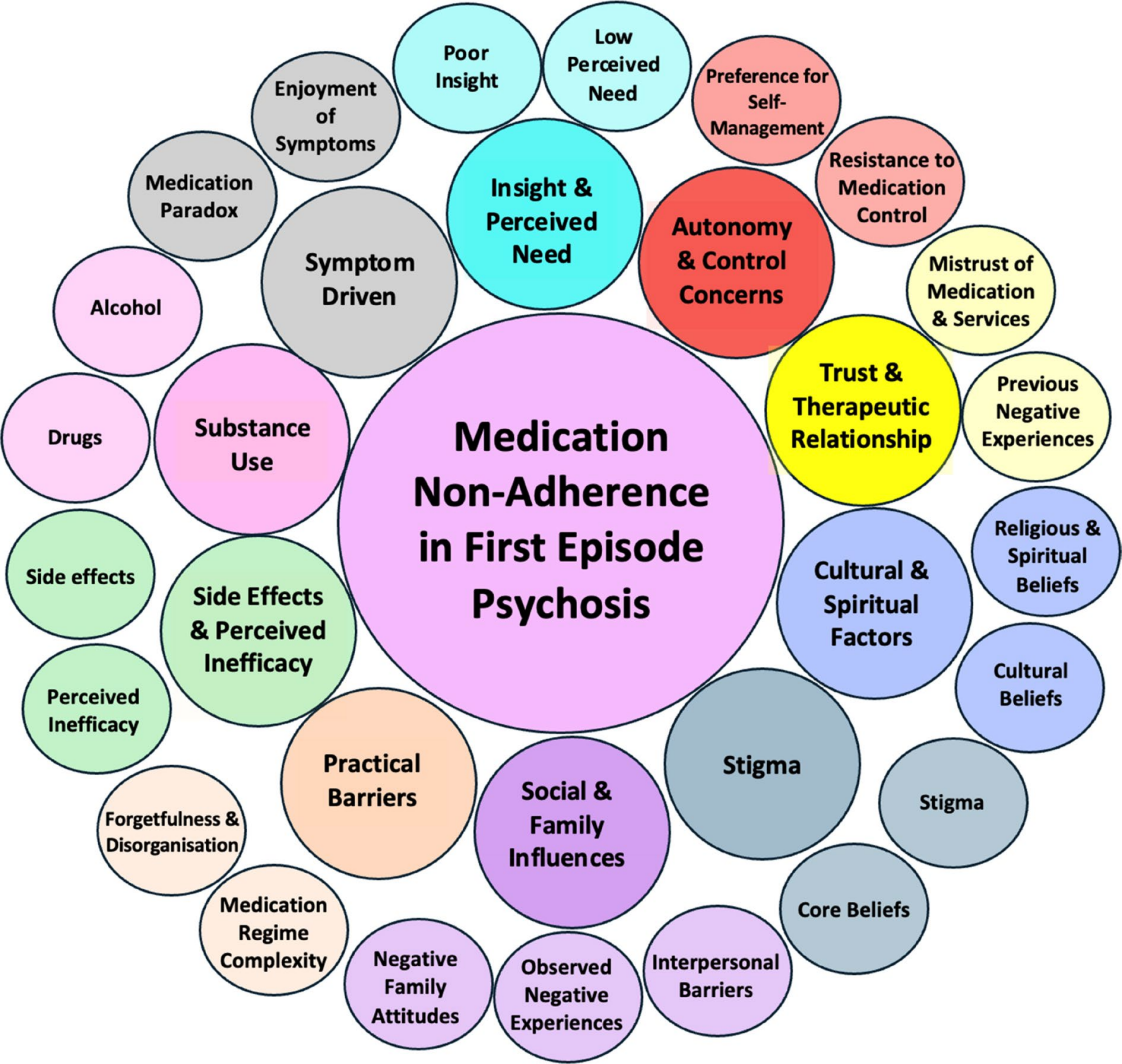
Visual representation of medication non‐adherence themes and reasons in FEP.

Medication non‐adherence in FEP appears to be influenced by a range of interrelated factors, including poor insight, low perceived need, preference for self‐management, mistrust of treatment, cultural or religious beliefs, substance use, prior negative experiences, side effects, forgetfulness and disorganisation, and social influences such as stigma and family attitudes. Negative core beliefs such as weakness/vulnerability, failure, and inferiority may further undermine adherence. Some clients may additionally resist treatment due to the perceived positive aspects of their psychosis experience, such as supportive voices or grandiose beliefs. Adherence is further complicated by the medication paradox, whereby early symptom improvement reduces clients' perceived need for ongoing treatment resulting in medication discontinuation. These findings illustrate how personal, relational, cognitive, cultural, and systemic factors converge to influence adherence behaviours. A visual representation of medication non‐adherence themes and reasons is presented in Figure 2, while Table 6 provides definitions for the reasons associated with each theme.

**TABLE 6:** Reasons for medication non‐adherence in FEP.

Theme	Medication non‐adherence reason	Definition
Insight and perceived need	Poor Insight	An individual's inability to recognise that they have developed psychosis and require treatment.
	Low Perceived Need for Medication	Individuals who perceive their symptoms as mild or non‐disruptive may view antipsychotic medication as unnecessary.
Autonomy and control concerns	Preference for Self‐management	Individuals prefer to cope with their symptoms independently, choosing to manage their condition without relying on medication for example, through exercise, diet, meditation and/or prayer.
	Resistance to Medication Control	Individuals resist taking medication due to fears of losing personal autonomy or feeling controlled by treatment.
Trust and therapeutic relationship	Mistrust of Medication and Mental Health Services	Scepticism or doubt regarding the safety, efficacy, or intentions behind treatment and healthcare providers.
	Previous Negative Experiences with Medication and Healthcare	Past adverse experiences with medications or negative encounters with healthcare providers.
Cultural and spiritual factors	Religious, Spiritual, and Cultural Beliefs	Religious/Spiritual and cultural factors shape individuals' beliefs and attitudes towards mental illness and its treatment, sometimes leading to rejection of medication in favour of alternative healing practices or faith‐based approaches.
Side effects and perceived inefficacy	Fear or Intolerance of Medication Side Effects	Concerns about or actual intolerance to the side effects of antipsychotic medications.
	Perceived Ineffectiveness of Medication	Feeling that antipsychotic medications do not improve symptoms or overall functioning.
Social and Family Influences	Observed Negative Treatment Experiences	Observing others experiencing adverse effects or poor outcomes from medication or mental health treatment.
	Negative Family Attitudes Towards Mental Illness and Treatment	Negative beliefs and stigma held by family members regarding mental illness and psychiatric treatment.
	Interpersonal Barriers to Medication Adherence	Difficulties within interpersonal relationships, such as conflicts or lack of support.
Stigma	Stigma	Negative attitudes, beliefs, and discrimination directed towards individuals with mental illness.
	Core Beliefs	Negative beliefs about the self that is failure, inferior, weak/vulnerable, defective, unlikeable, unlovable, worthless
Practical barriers	Complexity of Medication Regimen	Complex medication regimens, including multiple daily doses or use of several medications (polypharmacy).
	Forgetfulness and Disorganisation	Forgetfulness and disorganisation can impair an individual's ability to consistently follow medication schedules.
Substance use	Self‐Medication with Alcohol and Illicit Drugs	Use of alcohol or illegal drugs to manage or alleviate psychosis symptoms.
Symptom‐driven	Medication Paradox	The phenomenon whereby early symptom improvement reduces an individual's perceived need for ongoing medication, resulting in medication discontinuation.
	Enjoyment of Psychosis Symptoms/ Does not want to get rid of symptoms	An acknowledgment of the perceived positive aspects of one's psychosis experience. For example, hearing voices may prevent loneliness, provide company, or offer verbal encouragement, while some delusions may confer a sense of importance or comfort. As a result, the client may be reluctant to take medication that would diminish or eliminate these experiences.

## 5 Medication Adherence

### 5.1 Trust

Qualitative studies show that trust in staff is a key facilitator of medication adherence. FEP patients who felt safe and supported from admission, and who valued staff expertise, were more likely to adhere to their medication (Bjornestad et al. 2017; Hui et al. 2018; Polillo et al. 2022; Yeisen et al. 2017; Cowan et al. 2020; Penny et al. 2009; Vaingankar et al. 2020). Even those with negative inpatient experiences may maintain adherence if they trust Early Intervention Services (EIS) in outpatient care (Stewart 2013). Overall, these findings indicate that trust in staff and strong therapeutic relationships are associated with medication adherence in both inpatient and outpatient settings.

### 5.2 Therapeutic Alliance

FEP patients consistently identified a strong therapeutic alliance, characterised by open communication and shared decision‐making, as supporting medication adherence (Brown et al. 2013; Polillo et al. 2022; Stewart 2013). Adherent clients reported that staff provided clear information about medication side effects and treatment duration (Hickling et al. 2018; Hon 2012; Yeisen et al. 2017). A strong therapeutic alliance also mitigated negative medication attitudes and counteracted negative hospital experiences (Ocansey et al. 2025; Stewart 2013). These findings indicate that collaborative and communicative clinician–client relationships support medication adherence.

### 5.3 Acceptance of Bio‐Psychosocial Model

Qualitative studies indicate that adherent FEP patients often understood their illness through a bio‐psychosocial lens (Artaud et al. 2020; Chua et al. 2024; Perry et al. 2007; Vaingankar et al. 2020). For example, FEP patients who recognised the negative impact of cannabis on their symptoms tended to advise others to avoid it (van der Heijden‐Hobus et al. 2024). Additionally, FEP patients who accepted the need for medication alongside religious or spiritual explanatory models continued treatment while also seeking support from traditional faith healers (Bhikha et al. 2015; Islam et al. 2015; Penny et al. 2009; Rathod et al. 2023; Vaingankar et al. 2020). These findings suggest that adopting a bio‐psychosocial understanding can enhance willingness to engage with medication and can coexist with other cultural or spiritual beliefs.

### 5.4 Psychoeducation and Illness Insight

Qualitative findings report that improving FEP patients' understanding of psychosis and the importance of treatment facilitates medication adherence (Vaingankar et al. 2020; van der Heijden‐Hobus et al. 2024). Adherent patients consistently demonstrated greater insight, recognising both the effects of the illness and the risks of medication non‐adherence, including relapse (Baloush‐Kleinman et al. 2011; Elowe et al. 2022; Mutsatsa et al. 2003; Quach et al. 2009; Hui et al. 2018; Sapra et al. 2014). FEP patients who were fully informed at treatment initiation understood the rationale for medication and were able to recognise symptoms of psychosis (Bjornestad et al. 2017; Hon 2012). These findings suggest that FEP patients with greater understanding of psychosis and treatment rationale tend to show higher levels of medication adherence.

### 5.5 Motivation to Recover

Studies found that the desire to return to a normal life reinforced medication adherence in FEP (Bjornestad et al. 2017; Choi and Kweon 2023; Chua et al. 2024; Cowan et al. 2020; Hon 2012; Perry et al. 2007; Polillo et al. 2022; Vaingankar et al. 2020; van der Heijden‐Hobus et al. 2024). This motivation enhanced commitment to managing symptoms and engaging in recovery‐oriented behaviours. Fear of relapse further increased FEP patients' willingness to tolerate medication side effects (Artaud et al. 2020; Eisner et al. 2014; Gray and Deane 2016; Hon 2012; Sapra et al. 2014; Yeisen et al. 2017). Overall, these findings suggest that both motivation to recover and fear of relapse support medication adherence in FEP.

### 5.6 Symptom Relief and Functional Improvement

FEP studies found that adherence is reinforced by positive treatment outcomes, including symptom alleviation and improvements in affect and cognitive functioning (Brown et al. 2013; Chua et al. 2024; Dijkstra et al. 2024; Gray and Deane 2016; Hon 2012; Sapra et al. 2014; Stürup et al. 2023; van der Heijden‐Hobus et al. 2024; Yeisen et al. 2017). Observing tangible improvements can make medication feel less burdensome, as perceived benefits outweigh side effects (Hon 2012). These findings suggest that functional gains support medication adherence in FEP by demonstrating the value of ongoing treatment.

### 5.7 Use of Reminder Tools and Technology

Studies indicate that digital tools and apps can support medication adherence by helping FEP patients overcome forgetfulness (Domínguez et al. 2024; Steare et al. 2021; Terp et al. 2018). Pill trackers reinforce daily medication routines, while self‐assessment features demonstrate how medication non‐adherence affects symptom severity (Steare et al. 2021; Terp et al. 2018). These findings suggest that digital tools or reminder apps contribute to FEP patients' sense of control over treatment and engagement in recovery.

### 5.8 Social Support and Family Involvement

Studies indicate that encouragement and practical assistance from family, friends, and caregivers promote medication adherence in FEP (Bjornestad et al. 2017; Eisner et al. 2014; Hui et al. 2018; Intharit et al. 2021; Perry et al. 2007; Sapra et al. 2014; Stürup et al. 2023; Vaingankar et al. 2020; van der Heijden‐Hobus et al. 2024). Regular family involvement helps to maintain adherence and reduces relapse risk, particularly compared to FEP patients living alone (Hui et al. 2018). Relatives may take active responsibility for ensuring engagement with treatment (Penny et al. 2009). Peer support, including sharing experiences and listening to others, fosters motivation and a sense of normalcy (Stewart 2013; Vaingankar et al. 2020; van der Heijden‐Hobus et al. 2024; Yeisen et al. 2017). These findings suggest that social support from family and peers is associated with medication adherence in FEP.

### 5.9 Summary of Medication Adherence Themes in FEP

**FIGURE 3:**
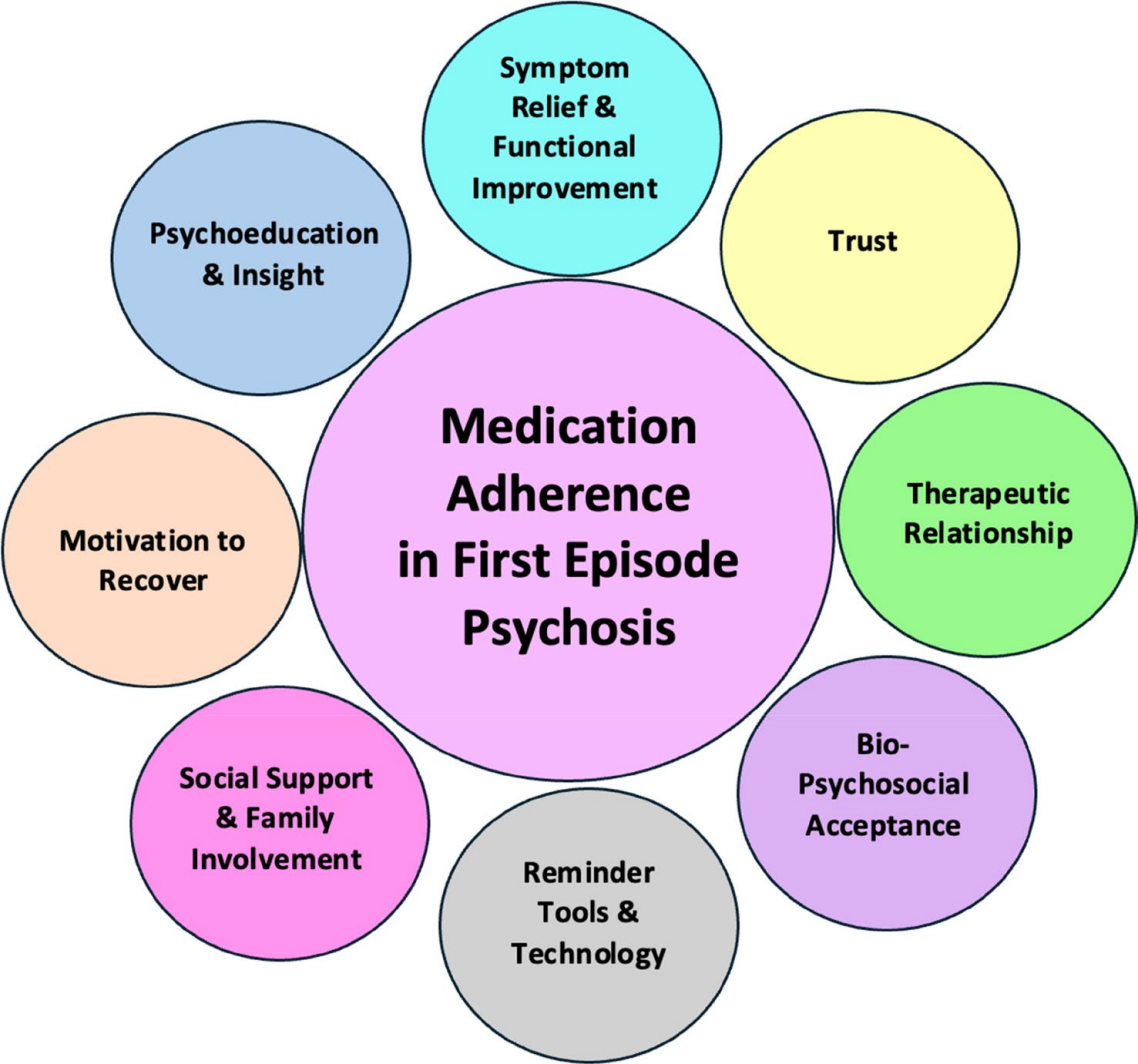
Visual representation of medication adherence themes in FEP.

Medication adherence in FEP is influenced by a combination of personal, relational, cognitive and practical factors. Personal factors include motivation to recover, fear of relapse, and the perceived benefits of symptom relief and functional improvement, which reinforce consistent medication use. Relational factors encompass trust in clinicians, a strong therapeutic alliance, and social support from family and peers, providing encouragement, accountability and engagement with treatment. Cognitive factors, such as psychoeducation and greater illness insight, enhance adherence by improving understanding of psychosis and the rationale for medication. Practical factors include the use of reminder tools and technology, which help clients manage forgetfulness and maintain regular medication routines. A visual representation of medication adherence themes is presented in Figure 3, while Table 7 provides definitions for each medication adherence theme.

**TABLE 7:** Medication adherence themes in FEP.

Medication adherence theme	Definition
Trust	Confidence in clinicians' competence, feeling safe on the ward, and valuing staff guidance. Trust in healthcare providers and prescribed treatment fosters positive attitudes towards medication and increases adherence.
Therapeutic alliance	Collaborative, open communication and shared decision‐making between clinician and patient. A strong alliance promotes engagement and mitigates negative attitudes towards treatment.
Bio‐psychosocial acceptance	Understanding psychosis through biological, psychological, and social lenses, while integrating cultural or spiritual beliefs. This holistic perspective encourages engagement with treatment, including adherence to medication.
Psychoeducation and insight	Knowledge about psychosis and treatment, awareness of relapse risk, and being fully informed at treatment initiation. Psychoeducation improves understanding of the illness and the need for treatment, supporting active medication adherence.
Motivation to recover	Desire to return to normal life, commitment to recovery‐oriented behaviours, and willingness to tolerate side effects. Strong personal motivation drives consistent adherence to medication and treatment plans.
Symptom relief and functional improvement	Experiencing reductions in symptoms and improvements in cognition, affect, and daily functioning. Observing tangible benefits reinforces the perceived value of medication and encourages continued adherence.
Reminder tools and technology	Use of digital reminders, pill organisers, apps, or self‐assessment tools. These aids support regular medication schedules and help overcome forgetfulness and disorganisation.
Social support and family involvement	Encouragement and practical assistance from family, friends, and peers, including shared experiences and monitoring. Supportive relationships promote adherence and provide accountability.

## 6 Psychosocial Interventions to Promote Medication Adherence

### 6.1 Cognitive Behaviour Therapy

CBT for medication adherence targets maladaptive beliefs about the illness and treatment via Socratic questioning and cognitive restructuring. These techniques aim to improve insight, attitudes towards medication and adherence‐related behaviours. Seventeen studies examined CBT‐based interventions to promote medication adherence in psychosis (see Table 5). Eleven reported significant improvements in medication adherence or attitudes towards medication, with one also showing reduced relapse rates (Brown et al. 2013). Three studies demonstrated improved insight in clients attending the active intervention compared with their Treatment as Usual (TAU) counterparts (Chien et al. 2024, 2015, 2016). Follow‐ups indicated sustained effects on medication adherence for up to 18 months, with one study showing improvement from baseline to 30 months (Chien et al. 2024, 2016; Gleeson et al. 2013; Lambert, Bock, et al. 2010). Six studies reported no significant improvements of CBT interventions on medication adherence, particularly among participants with chronic psychosis or high baseline adherence, suggesting ceiling effects. Non‐significant results were also associated with small sample sizes, low session attendance, or suboptimal intervention delivery (Anderson et al. 2010; Byerly et al. 2005; Gray et al. 2006; O'Donnell et al. 2003; Skarsholm et al. 2014; Uzenoff et al. 2008). In contrast, interventions targeting participants with poor baseline adherence and high session engagement showed significant improvements in adherence (Chien et al. 2015, 2016; Dikec and Kutlu 2016).

Overall, CBT‐based interventions appear effective for improving medication adherence in psychosis, particularly when attendance is high and baseline adherence is low. However, the independent effect of CBT is difficult to determine due to concurrent methods such as motivational interviewing and/or psychoeducation.

### 6.2 Psychoeducation

Psychoeducation involves providing clients with information about psychosis, treatment options, and the risks of medication non‐adherence (such as increased risk of relapse and ongoing symptomatology), aiming to improve understanding, insight, and active engagement with prescribed medication. Eighteen studies examined psychoeducation interventions to promote medication adherence in FEP (see Table 5). Sixteen reported significant improvements in medication adherence, one found no effect (Tessier et al. 2023), and one showed improved beliefs about medication adherence (Robinson et al. 2018). All six studies in first‐episode or early psychosis samples reported benefits for medication adherence or related attitudes. Twelve studies found sustained effects, lasting up to 30 months (Gleeson et al. 2013). Several studies also reported reduced relapse and hospitalisation, including one showing significantly lower hospitalisation risk in the intervention group (Kopelowicz et al. 2012). Family involvement was common (12 studies) and appeared beneficial. Five culturally adapted interventions were effective, highlighting the importance of cultural tailoring (Kopelowicz et al. 2012; Marchira et al. 2019; Ngoc et al. 2016; Oneib et al. 2025; Zhu et al. 2021). Three studies found reduced family stigma and more positive attitudes towards psychosis, suggesting psychoeducation can improve both adherence and family perceptions.

### 6.3 Motivational Interviewing

Motivational Interviewing (MI) for medication adherence uses a client‐centred approach to enhance motivation by resolving ambivalence and by exploring the advantages and disadvantages of adhering to, and not adhering to, antipsychotic medications. Fifteen interventions incorporated MI, with eight reporting significant improvements in medication adherence and relapse rates in psychosis samples, five of which included FEP samples (Brown et al. 2013; Chien et al. 2024, 2015, 2016; Dahan et al. 2016; Gleeson et al. 2013; Harmanci and Budak 2022; Staring et al. 2010). One study improved medication attitudes without directly measuring adherence, limiting conclusions about its effect on actual medication‐taking behaviour (Maneesakorn et al. 2007). Non‐significant findings primarily involved chronic psychosis samples, highlighting MI's particular suitability for FEP populations (Anderson et al. 2010; Barkhof et al. 2013; Byerly et al. 2005; O'Donnell et al. 2003; Schulz et al. 2013; Skarsholm et al. 2014).

Heterogeneity in adherence measures limited comparability across studies, but improvements in medication adherence generally coincided with positive changes in treatment attitudes. Few studies examined MI in isolation, with most combining multiple psychosocial approaches. Overall, MI appears to be effective for improving medication adherence and attitudes towards treatment, especially in early psychosis, highlighting the importance of targeting treatment attitudes to support adherence.

### 6.4 Adherence Therapy

Adherence Therapy (AT) is a structured, brief psychosocial intervention that combines elements of cognitive‐behavioural therapy and motivational interviewing to enhance medication adherence by addressing ambivalence, correcting misconceptions about medication, and promoting collaborative problem‐solving between clinician and client. Fifteen studies examined AT interventions (see Table 5). Six studies reported significant improvements in medication adherence (Chien et al. 2024, 2015, 2016; Dikec and Kutlu 2016; Kopelowicz et al. 2012; Staring et al. 2010), with one also showing reduced relapse rates (Brown et al. 2013). Five studies reported improved medication attitudes and satisfaction (Chien et al. 2024, 2015, 2016; Dikec and Kutlu 2016; Maneesakorn et al. 2007). In contrast, five studies found no significant changes in adherence (Anderson et al. 2010; Byerly et al. 2005; Gray et al. 2006; O'Donnell et al. 2003; Skarsholm et al. 2014), all involving chronic psychosis samples, whereas studies with significant effects comprised FEP samples.

Effective interventions tended to provide longer or more frequent sessions (6–24 sessions over 8 weeks–12 months), with flexibility to tailor to individual needs. Cultural adaptations were also associated with better outcomes (Chien et al. 2015, 2016; Kopelowicz et al. 2012). Interventions with shorter sessions (< 60 min) or fewer than six sessions were less effective. Overall, these findings suggest that AT can improve medication adherence, particularly in early psychosis, when interventions are culturally sensitive and sufficiently intensive to address individual client needs.

### 6.5 Assertive Community Treatment

Assertive Community Treatment (ACT) is a team‐based, intensive, community‐oriented intervention that provides comprehensive, individualised support, including medication management, psychoeducation, and psychosocial rehabilitation to enhance adherence, reduce relapse, and improve functional outcomes in individuals with psychosis. Compared to controls, ACT FEP participants were significantly more likely to adhere to medication (OR = 3.5 and 4.71; Lambert, Bock, et al. 2010; Randall et al. 2017) and demonstrated greater long‐term adherence (OR = 2.54; Randall et al. 2017). The ACT programs delivered psychosocial interventions across individual, group and family settings. Overall, these findings indicate that ACT is associated with improved medication adherence in FEP, with benefits maintained over the long term.

### 6.6 Adherence‐Focused Psychosocial Skills Training

Adherence‐Focused Psychosocial Skills Training (AF‐PST) is a structured intervention that teaches practical skills to support consistent medication use, including strategies for routine management, coping with side effects, problem‐solving, and enhancing motivation, with the goal of improving adherence and overall treatment engagement. All four studies targeting skills to support medication adherence reported significant improvements in medication adherence (Dahan et al. 2016; Staring et al. 2010; Valencia et al. 2012; Zhu et al. 2021), with two showing sustained effects at three and 6 months (Staring et al. 2010; Zhu et al. 2021). Follow‐up data were not reported in the other two studies, and only one included FEP participants (Valencia et al. 2012), highlighting the need for further research in early psychosis. All interventions combined multiple approaches, such as psychoeducation and motivational interviewing, so the specific effect of skills training alone on medication adherence remains unclear. Overall, these findings suggest that adherence‐focused psychosocial skills training can improve medication adherence, but further studies are needed to isolate its independent effects, particularly in FEP populations.

### 6.7 Summary of Psychosocial Interventions for Medication Adherence in Psychosis

A range of psychosocial interventions have demonstrated effectiveness in promoting medication adherence in psychosis, particularly in FEP populations. Cognitive Behavioural Therapy (CBT) and psychoeducation improved insight, attitudes towards medication, and adherence‐related behaviours, with sustained effects reported up to 30 months. Motivational Interviewing (MI) enhanced adherence by resolving ambivalence and targeting treatment attitudes, especially in early psychosis. Adherence Therapy (AT), combining CBT and MI elements, was effective when culturally adapted and delivered with sufficient intensity. Assertive Community Treatment (ACT) improved adherence and long‐term engagement through team‐based, community‐oriented support, while Adherence‐Focused Psychosocial Skills Training (AF‐PST) promoted practical skills for consistent medication use. Overall, interventions were most effective when baseline adherence was low, attendance was high, and interventions incorporated culturally sensitive, multi‐component approaches.

## 7 Discussion

Based on the findings of the present review, we have developed a fictional clinician–client to demonstrate how Socratic questioning can explore beliefs about medication adherence in FEP (see Table 8). For each medication non‐adherence reason identified in this review, we have also developed a brief and comprehensive list of Socratic questions and CBT strategies for addressing each of these reasons (see Tables 9 and 10, respectively).

**TABLE 8:** Clinician‐client transcript assessing medication adherence in FEP.

Clinician: So, how have you been doing recently? Client: Well, I'm experiencing some voices telling me I'm ‘good for nothing and worthless’, which is very distressing.
Clinician: I'm sorry to hear that, that sounds intense. Does anything help with the voices? [Explore voice coping] Client: Listening to music helps to drown out the voices.
Clinician: Ok so music helps, that's good. And are you taking any medication at the moment to help with the voices? Client: Yeah, I've been prescribed Aripiprazole 10 mg, but I stopped taking it 2 weeks ago.
Clinician: How come? Client: Well, I felt I was getting better and didn't need it anymore, so stopped taking it [Medication Paradox].
Clinician: Is it possible that the medication was working and led you to feel better? Client: Oh, I didn't think of that. I just thought I was getting better so thought, what's the point of taking the medication, as I don't need it. But if it was helping, then I feel silly for stopping it.
Clinician: No need to feel silly, that's an understandable response to starting to feel better [Normalisation]. Ok, so were there any other potential reasons for stopping the medication? Client: Well, I didn't like the side effects [Problems with side effects].
Clinician: What side effects were you experiencing? Client: I noticed that I was very drowsy, and numb to my emotions, and my foot wouldn't stop twitching. I also felt really restless and couldn't stop pacing, which was really annoying.
Clinician: OK, so maybe it wasn't the best medication match for you. Would you be willing to try another medication? Sometimes it takes a bit of trial and error to find the right medication and dose to suit each person. What are your thoughts on that? We could arrange a medication review for you so you can discuss this with your psychiatrist? Client: Can't I get better without the medication? [Not wanting to rely on medications‐ Preference for Self‐Management]
Clinician: Well, the clinical recommendation based on the evidence is that someone who has begun hearing voices/experiencing suspiciousness [symptom of psychosis] needs to take antipsychotic medication for 1–2 years after the onset of those experiences [Psychoeducation‐referring to NICE guidance]. Client: Can't I take the medication as and when I feel like I need it? Like when the voices are really intense?
Clinician: Well, that's not how this medication works. The medication takes a bit of time to start working in the body and we encourage clients to take it as prescribed by their psychiatrist, to take it consistently and not to interrupt the dosage. That's how the medication can work because sometimes chemicals in the brain in response to too much stress can become imbalanced and we just need some medication to stabilise those chemicals in the brain [Psychoeducation]. Client: I'm scared of being reliant on this medication. Do I have to take this medication forever?
Clinician: The idea is that people who come to our service will take antipsychotic medication for 1–2 years since the start of their unusual experiences. The medication can then be tapered off and discontinued in a gradual and safe way with regular monitoring by the team. Some people might not need medication for the rest of their lives. However, some people might still have some on‐going symptoms and might need some medication to help with any distressing experiences/symptoms. Client: I don't want to take medication forever. I don't want to have psychosis.
Clinician: I can understand that [Validation]. While we can't say for certain whether you'll need a low dose of medication after your 3 years with our early intervention service, the goal is to gradually reduce and eventually discontinue it. Your symptoms will be closely monitored throughout. If you're feeling well and symptom‐free, there may be no need to continue with the medication. However, if some symptoms persist, a low dose might help maintain your stability. It's important to remember that many of us need medication at some point in our lives—it's nothing to be ashamed of [Normalisation]. Client: But I don't want this label of psychosis
Clinician: Do you agree or disagree with the idea of having had psychosis [Checking for insight level‐ Full insight/partial insight/lacking insight] Client: I agree I had an episode of psychosis in response to stress with exams [Full Insight]
Clinician: And can I ask, what does it mean for you that you have had an episode of psychosis? [Eliciting beliefs about the illness] Client: It means that I can't cope with stress
Clinician: And if you can't cope with stress, what's the worst thing about that for you? What would it say about you as a person? (e.g., that you are weak/vulnerable, inferior, worthless, unlovable/unlikeable or a failure?) [Downward arrow technique to elicit core beliefs about the self] Client: It would mean I am weak and a failure
Clinician: Which of these two beliefs about yourself upsets you the most? Weakness or failure? [Identifying core belief that elicits the most distress] Client: Failure, that upsets me the most
Clinician: We can all become unwell at times. You mentioned last time that your uncle has schizophrenia so you might be at an increased risk of experiencing symptoms of psychosis [positive family history of psychosis]. If you could find ways to manage stress, avoid substances, get regular sleep, and take your medication as prescribed, how do you think those things might influence your ability to stay well? [Cognitive challenging via Socratic questioning] Client: I think all those things would really help. I actually could do a lot of things to keep well. I guess that makes sense. I am quite forgetful though, so if I just missed it here and there, would that be OK? [Forgetfulness]
Clinician: We can all be forgetful, that's totally understandable [Normalisation], but it's really no different from taking medication to manage something like diabetes. You have to take it consistently to manage the blood glucose levels, so the medication needs to be taken consistently for it to work effectively [Psychoeducation]. One thing that might help is pairing your medication with a routine activity—like taking it right after brushing your teeth at night or with your morning coffee. Linking it with something you already do regularly can make it easier to remember. That kind of habit‐stacking is a really helpful strategy for many people. Using a dosette box could also help to organise your tablets neatly in one place [Problem Solving]. Client: I like that. I can try and give that a go. I think having a routine would really help.
Clinician: Absolutely. And are there any other ideas you have to help you to remember? Client: Well, I guess I could set reminders on my phone or ask my mum to remind me [Social Support & Digital Tools]
Clinician: That's a good plan‐ having a family member remind you can be really helpful. I also really like the idea of setting a reminder on your phone, would you be willing to try that? I'm also wondering if there is anything you've experienced that's made you a bit hesitant or reluctant to take medication? [Eliciting negative medication beliefs]. Client: I think a big part of my reluctance is because of my uncle—he has schizophrenia, and I've seen him in and out of hospital all my life. I have memories of him being really sedated in hospital, like a ‘zombie’, and dribbling after taking medication. That's something I've always been scared of happening to me [Observed Negative Treatment Experiences]
Clinician: I can totally understand that, but a lot has changed regarding medications since your uncle became unwell, and there are fewer side effects with these newer medications. And as I said, it's about trying to find the right medication match for you. I would really be encouraging you to speak with your psychiatrist in our early intervention service so they can maybe adjust the dose or change the medication to find the right one for you. How does that sound? Client: I guess that makes sense, and if it's a match just for me then that might help with the voices. I was wondering though, could I not just use alcohol or cannabis? as they don't make me feel numb or give me side effects like the medication does. I find them relaxing and I can sleep better at night when I use these substances [Substance use]
Clinician: Well, we actually don't want you to rely on or use those substances as a crutch because that won't help you in the long term. If anything, substances actually interrupt the effectiveness of the medications that we prescribe as a team. And there is evidence that cannabis actually increases risk of having symptoms of psychosis like suspiciousness and voices. So, in a way you are reducing the effectiveness of the medication whilst also increasing the likelihood of experiencing symptoms of psychosis. So ethically we just have to encourage you to avoid alcohol and cannabis to support your recovery from psychosis [Psychoeducation] Client: That does make sense and if it's actually going to make the voices worse, then maybe I shouldn't use those substances.
Clinician: Mmm hmm, absolutely. Is there anything else that might get in the way of you taking the medication as prescribed? Client: Well, my family keeps telling me not to take medication and to just ‘toughen up and get better without relying on medication’ [Negative Family Attitudes].
Clinician: Is it ok to get better with the help of medication? Client: I guess, and I really do want the voices to stop so I can get back to college. But my family also encourage me to just pray because they believe that will help me more [Spiritual beliefs].
Clinician: Can you do both? We don't want to interfere with your spiritual or religious practices but does it have to be either or, could you do both? Client: Yeh I could do both. Maybe God wants me to accept the help your team offers.
Clinician: That's possible. I also wonder if it might be helpful to have a couple of meetings with you and your family so we can have a conversation about symptoms of psychosis and available evidence‐based treatments, such as medication and psychology so everyone is on the same page. How does that sound? Client: Yeah. I mean, sometimes I feel like they don't really understand what I'm going through, so it might be beneficial to talk to them as well.
Clinician: OK, great. So let's organise that. Let's have a family meeting to clarify and demystify anything about psychosis and the service and explain the purpose of the medication, alongside psychology and care coordination. Just so you have a package of support because it's not just one thing over the other. I can also provide some written information about medication, psychosis, and the service if that might be helpful for you and your family [Psychoeducation] I'm just aware of time, thank you for being your openness to discuss what's been going on for you. We did end up talking a lot about medication today, but I think it was helpful. How did you find our meeting today? Client: I found it very helpful, it was good to discuss my concerns about medication and possible ways forward, as I really do want to get better [Motivation to recover]
Clinician: Absolutely and you can get better, there is hope. That's what the early intervention team is here for [Installation of hope] Thank you again and I'll look forward to seeing you next week.

**TABLE 9:** CBT Socratic questions for medication non‐adherence reasons in FEP.

Medication non‐adherence reason	Socratic question
Poor Insight	If medication can help reduce stress, improve sleep, or support coping, is this something you'd be willing to try?
Low perceived need for medication	Have you noticed any difference in how you feel when you take your medication versus when you don't?
Preference for self‐management	Can medication be used alongside your existing coping strategies rather than replacing them?
Resistance to medication control	What would help you feel in control of decisions about your treatment?
Mistrust of medication and services	What past experiences have affected your trust, and what would help you feel safe now?
Religious, spiritual, or cultural beliefs	Could medical treatment support your recovery alongside your spiritual or cultural practices?
Self‐medication with alcohol or drugs	How do your symptoms differ when you use substances versus when you don't use substances?
Previous negative experiences with medication/services	What would help you feel more confident about trying medication again?
Fear or intolerance of side effects	Which side effects are most concerning, and how could we manage them together?
Perceived ineffectiveness of medication	Have you noticed any small shifts or changes in your symptoms since starting medication?
Observed negative treatment experiences	How do others' experiences with medication influence your own views?
Medication non‐adherence reason: Complexity of medication regimen	Socratic question: What aspects of your medication routine feel most difficult to manage?
Forgetfulness or practical barriers	What reminders or routines could help you take your medication consistently?
Negative family attitudes	How can we problem‐solve your family's concerns while supporting your treatment?
Stigma	How would taking medication affect your view of yourself and how others see you?
Core Beliefs	Does taking medication mean weakness, or could it be a sign of strength by taking control of your health?
Interpersonal barriers	Who in your social network influences your medication decisions, and how can you navigate that?
Medication paradox (feeling better leads to medication discontinuation)	Could continuing medication help maintain the progress you've made even when you feel better?
Enjoyment of Psychosis Symptoms/ Does not want to get rid of symptoms	How would you feel if you no longer heard the voice? If medication could help lessen or remove the voice, could we explore other ways for you to feel less lonely without it?

**TABLE 10:** CBT strategies for overcoming barriers to medication non‐adherence in FEP.

Medication non‐adherence reasons	CBT strategies
Poor insight	Clinician prompts: What is your understanding of what led you to our service? [Regardless of insight level] What disruption has this caused for you? [Regardless of insight level] If medication can lessen stress, improve sleep/mood, or help you cope, is this something you'd be willing to try? Possible client responses: I experienced too much stress and developed psychosis [Full Insight] I'm not sure, I had a spiritual experience that may have contributed to my intense beliefs [Partial Insight] The government has bugged my house and wants to persecute me [Lacking Insight] Clinician responses: That sounds like a lot of stress and disruption for you, am sorry to hear that [Validation] As part of our service, we offer care coordination so you have a point of contact in the service, we have psychology so you can talk about your experiences, and we can also offer some medication to help with stress, sleep, and mood, how does that sound? If medication can lessen the stress of what you are currently going through or help you to get better sleep during this period of stress, is that something you are willing to try? [For clients lacking insight, discuss the stress around the experiences, not the psychosis itself] CBT Strategies: Focus on stress and coping rather than challenging beliefs directly; use normalisation, problem‐solving, and collaborative goal setting to gently support engagement with treatment.
Low perceived need for medication	Clinician prompts: How do you feel about taking medication at the moment? Do you think the medication is helping in any way? And if so, in what way? Have you noticed any difference in how you feel when you take the medication in terms of your level of distress about what you have been going through? [psychosis symptoms/voices/delusions] Or in terms of your level of stress, sleep, and mood? Have you noticed any difference when you stop taking the medication? What have you noticed? Do you feel the same, better, or worse? Possible client responses: I feel fine/I can manage without it/I'm not sure it's necessary [Low perceived need] It helps reduce voices, improves sleep, balances mood/I'm willing to take it because it's helpful [High perceived need] Clinician responses: Can you track how you feel with and without medication over the week? What advantages or disadvantages do you notice when taking or missing doses? CBT Strategies: Self‐monitoring of symptoms and mood, guided reflection on medication effects, motivational interviewing to explore perceived need, psychoeducation on benefits and consequences.
Preference for self‐management	Clinician prompts: How do you usually cope when things become stressful or overwhelming? What strategies have helped you manage your experiences so far? What does managing things on your own mean to you? Could medication fit with your own coping strategies? Would you be open to using medication alongside your own strategies for a short period to see how it works for you? Possible client responses: I prefer to handle things naturally and not rely on medication [High preference for self‐management] I have always dealt with problems on my own, I do not want to start taking pills [High preference for self‐management] I have my own routines that help me stay grounded [High preference for self‐management] I might consider medication temporarily but I want to stay in control [Moderate preference for self‐management] I don't mind using medication to help me to get better, am willing to give it a go [Low preference for self‐management] Clinician responses: It's important to stay in control—medication can sometimes help you regain control over your health [Cognitive rephraming] Can you combine your own coping strategies with medication to maximise recovery? Medication could act as a short‐term tool alongside your strategies, not replacing them. Would you be open to a trial period to see if medication complements your coping methods? Early treatment improves long‐term outcomes, and different medications can be trialled to find the right match [Psychoeducation] If medication can reduce voices or suspiciousness, is that something you would be willing to try? CBT Strategies: Motivational interviewing, guided reflection, psychoeducation, collaborative problem‐solving, self‐monitoring.
Resistance to medication control	Clinician prompts: Do you have any concerns about taking medication, including worries about control or losing autonomy? Do you ever feel pressured by others to take medication? What would help you feel in control of your treatment? How could we work together so you feel in charge of important health decisions? Possible client responses: I do not want anyone telling me what to put in my body Taking medication feels like giving up control I do not trust anyone who forces me to take medication Clinician responses: My role is to support your recovery, not to impose treatment. You are an active participant in your care, and we want you to be a part of decisions about your health. We can discuss treatment options together at a pace that works for you. What does staying in control look like for you? Is taking medication a sign of weakness, or could it be a way to take back control and get back to your life? [Cognitive restructuring] Let's also consider the advantages and disadvantages of medication together. CBT Strategies: Motivational interviewing, guided reflection, cognitive restructuring, psychoeducation, collaborative decision‐making.
Mistrust of Medication and Mental Health Services	Clinician prompts: What makes it difficult for you to trust medication or mental health services? Have you had any past experiences—such as involuntary hospitalisation or feeling pressured to take medication—that have made it harder for you to trust services or treatment? What would help to rebuild that trust? What would make you feel safe in our work together? Possible client responses: I do not trust what the tablets do to people Mental health services do not listen; they just medicate I am worried there are hidden motives behind treatment Clinician responses: It's understandable to feel cautious, especially after difficult experiences. I'm sorry you didn't feel listened to in the past. Our team wants to understand what led you to our service and what has caused you stress. We can support you psychologically and with medication, providing holistic care. We will explain what the medication does and does not do so as to avoid surprises. We aim to work transparently so you are fully informed about your treatment options. CBT Strategies: Validation, psychoeducation, guided reflection, motivational interviewing, collaborative and transparent decision‐making.
Religious, spiritual, and cultural beliefs	Clinician prompts: Do your cultural or religious/spiritual beliefs affect how you view medication? In what way do these beliefs influence your view of treatment? Do you use any religious or spiritual coping strategies such as prayer or reading sacred texts? Are there particular verses, passages, or teachings from your faith or spiritual practice that you find comforting? If so, which ones? How do these practices help you in your recovery? Possible client responses: Medication is incompatible with my religious faith Prayer alone will help me to recover My experiences are spiritual, not something medication can treat My family believes I need an exorcism instead of medication I need to suffer in order to reach spiritual enlightenment Clinician responses: Could medication be used to support your recovery in addition to your religious coping? Does it have to be either/or, can it be both? Is it possible that God is sending you to our service to make use of treatment options available to help you to get better? Regardless of whether this is spiritual or psychosis, it has caused a lot of distress and disruption—would you agree? If medication could help to improve your sleep, mood, and/or reduce the intensity of your experiences, would you consider trying it? Would you be open to allowing the clinical team to complete an assessment or trial medication before pursuing an exorcism? Would you like us to involve a religious or spiritual leader in a session to explore their perspective on your experiences? Regarding demonic possession/spiritual enlightenment? Or whether this may be more related to psychosis caused by exam stress? What is your family's understanding of what you are going through, do they think it is a spiritual experience or mental health related? Could cultural or spiritual approaches and medical support work together? Some people use medication short‐term to reduce distress so they can fully engage in their spiritual practices, is that something you would consider? CBT Strategies: Motivational interviewing, guided reflection, psychoeducation, collaborative problem‐solving, integrating cultural/spiritual beliefs with medical treatment.
Self‐Medication with Alcohol and Illicit Drugs	Clinician prompts: Do you use alcohol or substances to cope with your voices/suspiciousness? [psychosis symptoms] What are the short‐ and long‐term effects of using these substances on your symptoms? Do your experiences differ when you use substances versus when you don't? Possible client responses: Drugs help to dampen down the voices better than the tablets do I use alcohol to calm me down so don't need medication Cannabis helps me to relax in the moment but makes me suspicious the next day I don't see the benefit of medication when I am using alcohol/substances Clinician responses: Substances can reduce the effectiveness of medication—were you aware of this? [Psychoeducation] Cannabis can increase suspiciousness and relapse risk—is that a risk you are willing to take? [Psychoeducation] Ethically, we advise reducing and stopping substance use as it worsens symptoms in the long‐term [Psychoeducation] Medication can help manage psychosis—would you consider gradually reducing substances? Could we explore alternative coping strategies (e.g., exercise, mood/sleep management, social support) instead of substances? What triggers your urge to use substances (e.g., loneliness, boredom, voices)? Would you be willing to try reducing or stopping substance use for a while, just to see if it makes a difference in how the medication works for you? CBT Strategies: Motivational interviewing, guided reflection, psychoeducation on substance effects, harm reduction, alternative coping strategy planning, self‐monitoring.
Previous negative experiences with medication and healthcare	Clinician prompts: Have you had any negative experiences with medication or services? What would help you to feel more confident with trying medication again? What did you dislike most about past treatments? Possible client responses: I was given medication without an explanation of what it was and why I needed to take it I had forced injections, which felt intrusive and controlling Police took me to hospital and I was forced to stay there for a month I didn't feel listened to The medication made me numb and detached and I don't want to feel like that again I felt dismissed or judged, no one believed me [about delusion] Clinician responses: I am sorry that you've had those difficult experiences regarding medication and mental health services Let's explore any specific concerns you have now so we avoid repeating past negative experiences What is your main concern about taking medication at the moment? What would help you to have a better experience this time round? For example, clear information, listening to your experiences, understanding past hospital admissions, reviewing previous medication and side effects? We can provide additional psychological support if you want to discuss your hospital admission further. CBT Strategies: Validation, guided reflection, psychoeducation, collaborative problem‐solving, relapse prevention planning, motivational interviewing.
Fear or intolerance of medication side effects	Clinician prompts: What side effects are you most worried about? Have you experienced any medication side effects before? What happened? (e.g., numbness/detachment/restlessness/twitching/mood changes)? How much do these concerns impact your willingness to take medication? A little/a lot? Possible client responses: I am scared of gaining weight or feeling sedated I had awful side effects last time I am sensitive to medication even small doses affect me negatively Clinician responses: That makes a lot of sense why you would be concerned about taking medication Which medication did you try, and would you consider trying another? It's about finding the right medication and dose for your body. We will monitor for any side effects and can adjust medication as needed—please report any concerns promptly. You will be closely supported and can contact your care coordinator for review. Are there certain side affects you are more willing to tolerate than others? CBT Strategies: Psychoeducation, guided reflection, validation, collaborative problem‐solving, self‐monitoring of side effects.
Perceived ineffectiveness of medication	Clinician prompts: How helpful or unhelpful has the medication felt so far? What changes were you hoping for when you started it? Have there been any small shifts even if not the full changes you expected? Possible client responses: It is not doing anything I feel exactly the same It helped a little but not enough Clinician responses: It is frustrating when medication doesn't seem to help but we can explore alternatives Sometimes small adjustments in dose or type can make a big difference Have you been taking your medication daily as prescribed? If yes: let's track any symptom improvement together over the next 2 weeks. For example, could you write down how many times you hear the voice in a day and rate it's intensity from 0% to 100% (0‐ not at all intense to 100% most intense) If not taking medication as prescribed‐ what tends to get in the way of you taking your medication, as prescribed as this will affect how well it works. CBT Strategies: Self‐monitoring of symptoms, guided reflection, psychoeducation, collaborative problem‐solving, motivational interviewing.
Observed negative treatment experiences	Clinician prompts: Have you ever seen friends or family have difficult experiences with medication? How have their experiences of treatment shaped your views of medication? Have you also seen examples where medication has been helpful for someone's recovery? Possible client responses: I remember my uncle being sedated and dribbling, like a ‘zombie’ and I don't ever want to be like that Taking medication would mean that I will end up just like my uncle My friend was sedated and withdrawn on antipsychotics I've seen someone I know stop doing things they enjoyed because of how the medication made them feel Clinician responses: It makes sense that seeing others' negative experiences would influence your feelings and perceptions about medication There are newer medications available now with fewer side effects than what your uncle might have been given People respond differently to medication, and your treatment will be personalised to avoid those side effects How long are you willing to wait for things to improve? 5, 10 years? If medication can help you within weeks or months, is that something you would be willing to try? If a medication doesn't suit you, we can change it. If it helps, that's great. CBT Strategies: Psychoeducation, guided reflection, cognitive restructuring, motivational interviewing, collaborative problem‐solving.
Complexity of Medication Regimen	Clinician prompts: How manageable does your current medication routine feel? How easy or difficult do you find your medication regime? What parts feel complicated or overwhelming? That is dose, type, timing? Would a simpler plan or once‐daily option help? Possible client responses: There are too many tablets to take at different times—it's hard to keep track. I lose track of what I'm meant to be taking. It's confusing. Clinician responses: How can we streamline your medication regimen to make it as simple as possible? What ideas do you have to make this simpler and more straightforward for you? Could you write out your medications and dosages and put it on the fridge and in your phone? Would a dosette box with days of the week help make it easier for you? We can also discuss long‐acting injections if you prefer less frequent dosing or prefer not to worry about taking oral tablets CBT Strategies: Behavioural strategies such as scheduling, reminders, and habit formation; problem‐solving to simplify the regimen where possible.
Forgetfulness and disorganisation	Clinician prompts: Do you ever forget to take your medication? What tends to happen? What usually gets in the way of you remembering to take your medication? How many doses in a week might you forget to take? What tends to lead you to miss a dose? What reminders or routines have helped you to remember to take your medication? Possible client responses: I'm exhausted by 7 pm so tend to fall asleep before taking it I fall asleep watching a film My routine is chaotic, so I forget I don't think about medication until it's too late I misplace my tablets Clinician responses: If you are exhausted by 7 pm, we might need to adjust the timing or the dose of your medication. If your schedule is chaotic, could you set a daily reminder on your phone or place a note on your bathroom mirror? Could we pair your medication with an existing routine—like brushing your teeth—to make it easier to remember? Is there anyone who could remind you to take your medication? CBT Strategies: Practical problem‐solving, use of external memory aids, habit formation, and environmental cues to establish routines and reinforce adherence behaviours; self‐monitoring, and guided reflection.
Family negative attitudes towards mental illness and treatment	Clinician prompts: How do your family tend to view medication and mental health? Do your family's opinions about medication influence your views on medication? Is there anything that your family do that you find helpful and supportive? Alternatively, is there anything that your family do that you don't find too helpful regarding your medication and mental health? Possible client responses: My family believe that medication is dangerous and that I should not take it They crush the tablets to give me a dose they think will help or withhold doses from me Talking about mental illness is taboo at home They discourage me from taking medication and encourage me to get better on my own Clinician responses: It is understandable to be unsure or confused about taking medication if your family are reluctant for you to take it I wonder if we can have a meeting with you and your family to share some information about psychosis, the service, and treatment options including medication and psychology, how does that sound? Research evidence has found that medication can help people recover from the experiences that you are going through [hearing voices/feeling suspiciousness] and a delay in starting treatment can make these experiences worse in the long term [Psychoeducation] Can you have a think about what you and your family's specific worries are about taking medication so we can problem‐solve this together? What makes your family believe the medication is dangerous‐ all medications are approved and follow clinical guidelines What makes your family crush or withholds tablets from you?—Medication needs to be taken as prescribed for it to work effectively If mental health is a taboo topic at home, do you feel comfortable talking to us in the early intervention service? We can also invite your family to help them understand what you're going through, if you think that would be helpful. As you are an adult, decisions about your health belong to you, but we can think together about how you might communicate with your family about your treatment if that feels useful. CBT Strategies: Family‐focused CBT, psychoeducation, communication skills training, collaborative problem‐solving.
Stigma	Clinician prompts: Do you have any concerns about how others might view you if you were to take medication? What do you think would happen? [fear or rejection/discrimination] How would being on medication impact your view of yourself? [Explore core beliefs for example weak/vulnerable, failure, inferior, unlovable, unlikeable] Possible client responses: People will think I am crazy I do not want to be labelled as ‘mentally ill’ I do not want to be seen taking medication If I take medication, it will mean I am weak and inferior People will reject me if they know I take medication My community say that medication is a sign of weakness and you just need faith to recover Clinician responses: Who actually needs to know what you are going through and what you have been through, can you have those conversations with the people closest to you that you trust? [Explore self‐disclosure] Does everyone need to know about your treatment? Some people can be judgemental, but I wonder if that says more about them than it does about you? Can you gradually test out social situations despite fears of judgement and see whether your feared predictions come true? Don't we all become unwell sometimes and use medication? Is there anything wrong with that? Medication does not have to define who you are‐ it is simply a tool to support your recovery and help you move towards your goals. If anything, taking medication is a sign of strength because it shows you are determined to get better. Accepting help shows a willingness to recover and get back to the life you want to lead. Would you agree or disagree? CBT Strategies: Challenging core beliefs and cognitive restructuring to address internalised stigma, behavioural experiments/activation to reduce avoidance, social skills training to manage anticipated stigma.
Core beliefs	Clinician prompts: When you think about taking medication, what does it say about you as a person? For example, do you think that taking medication would mean that you are weak/vulnerable, defective, inferior, worthless or a failure? How do you think others would view you if you were to take medication? For example, as unlikeable, unlovable, weak/vulnerable, defective, inferior, worthless or a failure? Possible client responses: If I take medication, then it means that I am weak and a failure People will see me as broken [defective] or incapable [failure] It makes me feel inferior and worthless Clinician responses: You mentioned beliefs about weakness and failure—which of these feels most upsetting for you? Have you ever felt this way before in your life? [linking to earlier experiences] What evidence supports, and what evidence challenges, the idea that needing medication means weakness/failure? Could taking medication also be seen as an act of strength, in the sense that you are taking steps to support your recovery? If someone you cared about were in the same situation, how would you view them? What are the advantages and disadvantages of believing this belief? For example failure What are the advantages and disadvantages of dwelling on this belief? For example failure What alternative, compassionate, incompatible belief would you prefer to believe instead? For example I am resilient, strong, worthwhile, valuable What evidence supports this alternative compassionate belief? CBT Strategies: Cognitive restructuring of negative core beliefs, developing alternate compassionate self‐beliefs
Interpersonal barriers to medication adherence	Clinician prompts: Is there anyone in your social network who discourages you from taking your medication? Do you worry about how others might react to you taking medication? Does anyone pressure you—either to take medication or not to take it? Possible client responses: My parents disapprove of my medication Friends make negative comments about me because I take medication I do not feel I have social support Clinician responses: Let's think together about how interpersonal pressures may be influencing your decisions about medication. We can explore how you might talk with family or friends about this, and we can role‐play those conversations if that would be helpful. If you think it could support your recovery, we can also arrange a family meeting to share information about psychosis, the service, and treatment options so your perspective is better understood. How does that sound? CBT Strategies: Social skills training, assertiveness training, role‐playing, guided problem‐solving to address interpersonal barriers to adherence.
Medication Paradox	Clinician prompts: When you start to feel better, how do you feel about continuing your medication? Do you usually continue taking it or do you stop taking it? Do you associate feeling better with the medication, or with something else? What tends to happen after you stop taking it? Do you feel the same, better, or worse in terms of mood and symptoms? Possible client responses: What's the point of taking medication when I feel better? I feel normal again so the tablets feel unnecessary I stop when I feel okay because I think I am cured I will take the medication as and when I feel I need it, maybe a couple of times a week I only take medication when the voices are really loud or when I'm really suspicious Clinician responses: It is common to feel less need for medication when symptoms improve I wonder if feeling better actually means that the medication is working so continuing with it helps maintain that progress Instead of stopping your medication as soon as you feel better, could we think about a gradual, monitored reduction plan so you stay safe and supported? CBT Strategies: Psychoeducation on relapse risk, cognitive restructuring of ‘feeling cured’ beliefs, collaborative problem‐solving to plan safe and sustained zadherence or gradual monitored discontinuation.
Enjoyment of Psychosis Symptoms/Does not want to get rid of symptoms	Clinician prompts: How would you feel if you no longer heard the voice? How would you feel if you no longer thought you were the king of England or that you were being monitored by the police? Possible client responses: I would feel lonely; the voice is encouraging and supportive. I would not feel special anymore if I believed I wasn't royalty. I would feel unsafe and unimportant if the police stopped monitoring me. Clinician responses: Ok, are there any negatives to what you are currently experiencing? For example, problems with friends, family, colleagues, or your ability to do things you want to do, like go to the shops, socialise, or work? [client: Yes, I isolate myself, socially withdraw, and can't work] So there are some downsides to your experience? [client: Yes] What would you like your life to be like? [client: I would like to feel more stable in my mood and to go back to college] If medication could help you with that, would you be willing to give it a go? Even if you might miss the voice or miss feeling special or looked after [by the police]? [client: I'm not sure] What is more important to you? Hearing a supportive voice or finding someone in your life that could do the same thing? [client: I would prefer to get that support from someone in my life, like my mum] So that's something we can explore, how to get mum or someone else in your life to provide verbal support Could we find other ways for you to feel less lonely and more valued and cared for, without it causing so much disruption in your life? For example, like dropping out of college, focusing your whole day on what the voices say, or watching for police cars? What ideas do you have? CBT Strategies: Exploration of the pros and cons of psychosis symptoms, balancing valued experiences with functional goals, cognitive restructuring of beliefs about voices or delusions, problem‐solving and behavioural planning to find alternative ways to meet emotional needs (companionship, purpose, sense of safety) without excessive disruption, and supporting gradual engagement with medication as a tool to improve stability and functioning.

This systematic review investigated the reasons, attitudes, and beliefs underlying both medication non‐adherence and medication adherence in FEP, as well as psychosocial interventions designed to promote medication adherence in psychosis. Nineteen reasons for medication non‐adherence in FEP were identified, which were synthesised into 10 overarching themes including: Insight and Perceived Need, Autonomy and Control, Trust and the Therapeutic Relationship, Cultural and Spiritual Factors, Side Effects and Perceived Inefficacy, Social and Family Influences, Stigma, Practical Barriers, Substance Use and Symptom‐Driven Factors (see Figure 2). Conversely, facilitators of adherence included illness insight, motivation to recover and fear of relapse, trust in clinicians, acceptance of the bio‐psychosocial model, experiencing positive treatment outcomes, use of digital reminders and social support. These findings suggest that psychosocial interventions targeting both barriers to adherence and adherence‐promoting factors may offer a comprehensive approach to addressing medication non‐adherence in FEP. Psychosocial interventions found to be effective in promoting medication adherence in FEP included CBT, psychoeducation, motivational interviewing, and family‐focused approaches that also explored cultural and religious/spiritual perspectives regarding medication adherence. These approaches may reduce non‐adherence by simultaneously addressing cognitive, relational, and cultural determinants of medication non‐adherence in FEP.

The present findings regarding medication non‐adherence in FEP may be understood via the concept of Recovery Style, which is defined as a patient's style of recovery, coping, or adaptation in response to having had psychosis (McGlashan 1987). Patients typically fall into one of two distinct categories, namely ‘integration’ or ‘sealing over’ (Drayton et al. 1998; Levy et al. 1975; McGlashan et al. 1975). A client with a sealing‐over recovery style minimises their psychosis experience and views it as an isolated and encapsulated interruption in their life (McGlashan 1987). They view their psychosis experience as something that is entirely separate and distinct from themselves and disincline to explore or understand it further, independently or with others (McGlashan 1987). This closely aligns with several themes associated with medication non‐adherence in this review, including poor insight, autonomy concerns, stigma and resistance to medical control. By contrast, an integrative recovery style refers to a patient who accepts and incorporates their psychosis experience into their life, attributes its cause to their life context, and uses it as a source of new information about themselves, their conflicts, relationships and behaviour (McGlashan 1987). These patients are curious and think flexibly about their psychosis experience and seek to enlist the help of others in order to understand it further (McGlashan 1987). This parallels the themes associated with medication adherence, such as acceptance of the bio‐psychosocial model, trust in clinicians and recognition of relapse risk. Incorporating an understanding of recovery style into clinical practice may help identify individuals at higher risk of medication non‐adherence and guide tailored psychosocial interventions. Clinicians can infer a client's recovery style—integrative or sealing‐over—based on their curiosity and openness to discuss their psychosis experience versus a tendency to minimise or avoid it, which can inform expectations about adherence and the support needed to engage with treatment.

Integrative and sealing over recovery styles have been characterised by different clinical outcomes in psychosis. Compared to integrative patients, sealing over patients demonstrated a four‐fold risk of involuntary readmission (O'Donoghue et al. 2011), decreased chances of remission (Staring et al. 2011), and poorer outcomes, including global functioning (Modestin et al. 2009), long‐term functional outcome (McGlashan 1987), and quality of life (Stainsby et al. 2010). A sealing over recovery style has also been associated with poorer engagement with services in terms of attending appointments, collaboration with mental health professionals, help seeking and treatment adherence (Startup et al. 2006; Tait et al. 2003). In terms of Cognitive Behaviour Therapy for psychosis (CBTp), a sealing over recovery style has been associated with less agreement on therapy goals, lower engagement, and poorer bonds with the therapist (Startup et al. 2006). These converging findings emphasise the clinical relevance of recovery style for understanding medication non‐adherence in FEP.

In addition to Recovery Style, negative core beliefs about the self, such as perceiving medication‐taking as a sign of weakness, inferiority, and/or failure, are prominent in FEP and emerged as a modifiable cognitive contributor to medication non‐adherence (Jorovat et al. 2025). These beliefs often interact with stigma, identity development, and autonomy concerns, perpetuating experiential avoidance and selective engagement with treatment. Interventions that target negative self‐beliefs can reframe medication‐taking as a proactive, solution‐focused strategy for fostering resilience and supporting recovery.

In addition to negative core beliefs, emotional processes may influence attitudes towards medication and engagement with treatment in FEP. Difficulties in emotional awareness, heightened emotional reactivity, and maladaptive coping can amplify distress from symptoms or side effects, reinforcing avoidance behaviours and thereby undermining medication adherence (Gurnani and Georgiades 2025). Targeting emotional dysregulation alongside cognitive distortions may therefore enhance treatment engagement and support sustained recovery in FEP (Gurnani and Georgiades 2025).

The factors contributing to medication non‐adherence are dynamic and may exert differing levels of influence over time (Artaud et al. 2020). Following the acute phase, many participants discontinue medication, consistent with reports that 74% of individuals with chronic psychosis stop treatment within 18 months (Lieberman et al. 2005). This highlights the importance of ongoing monitoring and intervention, as adherence challenges may persist or evolve across the illness trajectory, reinforcing the need for flexible, stage‐sensitive psychosocial strategies.

Improvements in symptoms can alter attitudes towards medication, with some clients perceiving ongoing treatment as unnecessary. At the same time, the perceived burden of side effects may outweigh the benefits previously experienced. Without adequate clinical guidance, clients may prematurely discontinue medication and seek alternative approaches (Béchard et al. 2025). Providing continued support during periods of stability is therefore essential to maintain recovery motivation and reinforce the understanding that non‐adherence substantially increases the risk of relapse.

Negative experiences with medication, healthcare, and observing others' treatment trajectories were frequently cited as reasons for non‐adherence in outpatient settings. This aligns with evidence that poor relationships with clinicians and adverse inpatient experiences are significant predictors of negative treatment attitudes (Day et al. 2005). Coercive treatment further heightens vulnerability to medication non‐adherence (Jaeger et al. 2013), and such experiences were disproportionately reported among ethnic minority groups (Devonport et al. 2023). The intersection of coercion, perceived injustice, and exposure to distressing care environments appears to compound mistrust, particularly among minoritised populations. Rebuilding trust and positive treatment experiences in outpatient contexts is therefore critical, as these environments can either perpetuate prior negativity or foster renewed engagement.

Comorbid conditions may further exacerbate adherence difficulties. One study reported that 37.7% of FEP participants were neurodivergent, with these individuals experiencing longer hospitalisations and more frequent crisis service contact (Nikolić et al. 2025). Medication non‐adherence is particularly prevalent among FEP patients with comorbid autism spectrum disorder (Downs et al. 2017), who may experience heightened mistrust towards medication and healthcare services. This area remains under‐researched and warrants further investigation.

Stigma also exerts a strong influence on medication non‐adherence. Qualitative evidence suggests that individuals perceive antipsychotic medication as a visible reminder of being ‘abnormal’ (Béchard et al. 2025). The psychological flexibility model of medication adherence in psychosis provides a useful framework for understanding this phenomenon: experiential avoidance may drive disengagement from treatment (Moitra and Gaudiano 2016). Feelings of shame, embarrassment, and anticipated judgement can become fused with self‐identity, prompting avoidance of medication that symbolises psychiatric diagnosis. These processes of cognitive fusion and avoidance may serve as key psychological mechanisms underpinning medication non‐adherence in FEP.

Religiosity and spirituality have also been found to influence medication adherence behaviour (Westhead and Georgiades 2025). Non‐adherent individuals frequently conceptualised psychosis through religious or spiritual frameworks, seeking alternative healing practices. Conversely, adherent individuals were more likely to integrate religious group practice and endorse bio‐psychosocial explanatory models (Borras et al. 2007; Ghanem et al. 2023). While rigid spiritual conceptualisations may undermine adherence, integrative belief systems that harmonise spiritual and biomedical understandings may promote it. Further research exploring these complex interactions across cultural contexts is thus warranted.

Social connection emerged as another crucial factor influencing adherence. Families and peers can hinder adherence through negative attitudes towards illness or treatment (Velligan et al. 2017) and may actively pressure clients to discontinue medication (Béchard et al. 2025). Psychoeducation programmes have proven effective in improving adherence, likely because they enhance understanding among relatives and reduce stigma (Kuipers et al. 2010). Their success may depend on the emphasis placed on medication management. Culturally adapted interventions can enhance adherence by acknowledging clients' cultural and spiritual beliefs and framing treatment in a way that respects these beliefs while supporting medication use (Degnan et al. 2018).

Substance use was also reported as a factor influencing medication non‐adherence, consistent with the self‐medication hypothesis (Hall and Queener 2007). Some individuals used alcohol, cannabis, or other illicit substances to manage symptoms or affective distress. However, evidence suggests that self‐medication accounts for only a minority of cases, as many individuals use substances for social or hedonic reasons that often precede illness onset (Kolliakou et al. 2015). Nonetheless, a subset of clients may rely on substances to regulate emotional discomfort or manage side effects, warranting clinical attention.

Cognitive deficits, particularly in executive functioning, may also explain medication non‐adherence related to forgetfulness and disorganisation. Executive dysfunction is evident in individuals with FEP (Tschentscher et al. 2023; Catalan et al. 2024) and has been associated with poor medication adherence (Na et al. 2015; Robinson et al. 2002). These findings underscore the importance of technological reminders and cognitive support tools to sustain adherence among FEP clients.

The findings from psychosocial interventions highlight the value of addressing medication non‐adherence early in the illness trajectory. Studies involving FEP samples reported more favourable adherence outcomes, although few demonstrated sustained effects at 24 months. Booster sessions may therefore be required to maintain long‐term benefits. Many interventions combined Motivational Interviewing, Cognitive Behaviour Therapy, psychoeducation, skills training, or family work, reflecting the multifactorial nature of medication non‐adherence. While intervention duration and intensity varied considerably, shorter or low‐dose interventions may limit the development of therapeutic alliances, increasing the risk of poor engagement (Tschuschke et al. 2022). Notably, 10 of 11 interventions incorporating group components reported significant improvements in adherence, suggesting that peer disclosure and support can positively reshape attitudes towards medication (Caruso et al. 2013).

Together, these findings indicate that medication adherence in FEP is best supported through relationally grounded, culturally sensitive, and multifaceted interventions that engage both clients and their social networks early in treatment. Sustained collaboration, psychoeducation, and the incorporation of client preferences into treatment planning remain essential to promoting long‐term adherence and recovery.

### 7.1 Strengths and Limitations

Synthesising findings from both qualitative and quantitative studies was a major strength of this review. Qualitative data enabled an in‐depth exploration of clients' subjective experiences, providing richer insight than studies relying solely on quantitative measures of medication attitudes (Hogan et al. 1983; Horne et al. 1999; Thompson et al. 2000; Weiden et al. 1994). This approach added nuance to complex areas such as religious and spiritual beliefs, which can shape how clients perceive and engage with treatment. The reasons identified offer clear, modifiable targets for psychosocial interventions, such as CBT, where clinicians can address individual beliefs, attitudes, and experiences related to medication and treatment more broadly. By focusing exclusively on FEP clients, the findings are more representative of the specific challenges encountered early in the illness course. This is important given that individuals with more chronic psychosis often develop different treatment attitudes (Sapra et al. 2014; Hui et al. 2006). Moreover, most interventions included follow‐up assessments, allowing inferences about the longer‐term effectiveness of psychosocial approaches for improving medication adherence.

However, some limitations should be acknowledged. Ceiling effects were evident in some intervention studies, where high baseline adherence may have reduced the potential for detecting statistically significant improvements (Harmanci and Budak 2022). Much of the available evidence originates from Western settings, with the remainder primarily from Asian countries and few studies from Africa. Consequently, generalisability to non‐Western contexts is limited, as cultural differences in beliefs about medication are well‐documented (Ghanem et al. 2023). Additionally, mistrust towards services among non‐adherent individuals suggests that aspects of covert non‐adherence may remain underrepresented in the literature. Finally, despite comprehensive search strategies, some relevant studies may not have been captured, particularly those addressing medication non‐adherence in FEP indirectly or within broader psychosis populations.

### 7.2 Clinical Implications

The findings of this review highlight multiple opportunities for clinicians to address medication non‐adherence through targeted and individualised approaches. The reasons identified are modifiable and can be explored within the context of NICE‐recommended psychosocial interventions for FEP such as CBT (NICE 2014). To support clinicians, we developed practical tools: visual diagrams of medication non‐adherence and medication adherence themes, structured Socratic questions and CBT strategies for each medication non‐adherence reason, and a clinician–client transcript demonstrating their clinical application. These resources are designed to support clinicians in conducting comprehensive assessments, developing personalised formulations, and delivering targeted CBTp interventions to address medication non‐adherence in FEP.

Side effects appear particularly distressing for individuals experiencing their first episode of psychosis, who may be encountering sedation, weight gain, or sexual dysfunction for the first time. Clinicians would benefit from placing greater emphasis not only on psychoeducation about potential side effects, but also on collaborative discussions to determine which effects an individual is most willing to tolerate. Involving family members or caregivers, where appropriate, may further enhance adherence. Family‐inclusive psychoeducation addressing both psychosis and medication can counter negative attitudes, reduce stigma, and promote sustained medication adherence.

Embarrassment about taking medication may be an under‐recognised additional barrier in FEP, with clinicians potentially overlooking its impact on adherence. Addressing these concerns, particularly regarding public or repeated daily dosing, and normalising medication use within the stress‐vulnerability framework (Zubin and Spring 1977; Georgiades et al. 2023), could help reduce shame and stigma while promoting acceptance of the bio‐psychosocial model.

Substance use can interfere with the effectiveness of medication and increase relapse risk, making routine assessment and intervention a critical component of FEP care. Clinicians can explore clients' motivations for use while providing psychoeducation on the interactions between substances, such as cannabis and antipsychotic medication. Collaborative work on substance misuse may strengthen clients' understanding of risk and support more stable recovery trajectories.

Exploring clients' values and hopes for the future (i.e., wish to return to college/get back to work), whilst also highlighting the factors that increase risk of relapse (i.e., continued cannabis use/poor sleep/rumination) in the context of bio‐psychosocial model, may enhance their insight, motivation to recover and adherence to medication. If clients are hesitant to give up cannabis use—a known risk factor for psychosis—employing a motivational interviewing approach to explore the advantages and disadvantages of its use may be helpful. While cannabis may provide short‐term relaxation, it carries longer‐term consequences such as exacerbation of psychosis symptoms and reduced efficacy of antipsychotic medication, both of which contribute to an increased risk of relapse. It can be useful to ask clients whether this is a risk they are willing to take and to encourage reflection on previous hospital admissions, which most clients generally recognise as disruptive and undesirable. Emphasising that clients have an active choice and responsibility over their own recovery, medication use and relapse risk reinforces agency.

If uncertainty about giving up cannabis remains, clinicians may use cognitive irony by asking clients what they ‘enjoyed’ about their hospital experience—highlighting the contradiction between the perceived short‐term benefits of cannabis and the undesired consequences of relapse. This approach mirrors Dialectical Behavioural Therapy (DBT) strategies of extending logic to reveal contradictions in a Socratic, non‐shaming way, promoting insight and motivation for change. Moreover, having explicit discussions about employing active problem‐solving, rather than passive coping, may further enhance adherence and reduce relapse risk. For example, ‘what can you do to prevent relapse?’ (i.e., take medication as prescribed, manage sleep, interrupt ruminative thinking, etc.).

When addressing medication non‐adherence in FEP, clinicians should adopt a therapeutic style that is warm, supportive and collaborative, while also being direct and pragmatic. This involves clearly outlining the consequences of unhelpful behaviours, highlighting the benefits of adherence, and avoiding collusion or the downplaying of potential risks, in order to foster insight, personal responsibility, and sustained engagement with treatment.

Engaging with clients' religious, spiritual, and cultural explanatory models is also essential. Clinicians would benefit from cultivating an attitude of openness towards alternative belief systems, creating space for these perspectives to coexist alongside a bio‐psychosocial conceptualisation of psychosis. Integrating these religious/spiritual and bio‐psychosocial frameworks could enhance trust and promote adherence, while acknowledging clients' parallel help‐seeking behaviours, such as spiritual or religious healing practices.

Medication non‐adherence may also be associated with the perceived positive aspects of psychosis, such as supportive voices or grandiose beliefs. Clinicians should examine the disruptions these experiences cause (i.e., family conflict, police involvement, and/or hospitalisation) and support clients to identify real‐life alternatives to meet the underlying needs these experiences serve. For instance, social support from family and friends can address feelings of loneliness typically fulfilled by encouraging voices, while engagement in meaningful activities can provide a sense of self‐worth similarly experienced during grandiose beliefs. Medication can then be framed as a tool to manage psychosis while preserving agency and promoting real‐life coping strategies.

Moreover, attitudinal shifts following the acute phase of psychosis warrant particular clinical attention. As symptoms subside, clients may question the necessity of continued medication. Regular review of beliefs about treatment, coupled with tailored psychoeducation at this stage, can reinforce the rationale for ongoing adherence and reduce relapse risk.

### 7.3 Future Directions

Future research could further examine differences in the reasons for medication non‐adherence between individuals experiencing FEP and those with more chronic forms of the illness. Existing evidence suggests that adherence is dynamic and influenced by multiple interacting factors (Higashi et al. 2013; Velligan et al. 2017; Wade et al. 2017). Longitudinal studies could help clarify whether specific reasons for medication non‐adherence are more prominent at different stages of the illness trajectory. Additionally, cognitive difficulties that affect clients' ability to remember and organise their medication regimens highlight the importance of exploring adherence support strategies. Future studies could also investigate which aspects of reminder tools and adherence technologies are perceived as unhelpful or burdensome by clients to improve the usability and sustainability of such supports.

Future research could also examine the role of negative core beliefs and emotional processes in influencing attitudes towards medication and treatment engagement to inform the development of targeted psychosocial interventions for FEP.

Future research investigating psychosocial interventions to enhance medication adherence would benefit from addressing recruitment limitations to minimise the likelihood of ceiling effects due to high baseline adherence. When initial adherence is already high, intervention effects may be underestimated. Future studies could mitigate this by enriching samples to include individuals with lower baseline adherence, thereby enabling clearer assessment of intervention efficacy.

## 8 Conclusion

This systematic review identified the subjective reasons for medication non‐adherence in FEP, with the aim of informing targeted CBT strategies. Medication non‐adherence was associated with poor insight, a low perceived need for medication, and concerns regarding personal autonomy, including fears of coercion and a preference for self‐management. Core beliefs about the self, such as perceptions of weakness, inferiority, and/or failure, also influenced adherence and represent important clinical intervention targets. Religious and spiritual explanatory models further shaped treatment engagement and are important to recognise and integrate into culturally sensitive clinical practice. Substance use was often employed as a form of self‐medication for psychosis symptoms, medication side effects, and affective distress, potentially reducing the perceived need for prescribed medication. Family dynamics and interpersonal relationships also influenced medication non‐adherence, underscoring the importance of psychoeducation for both clients and caregivers. Negative experiences with healthcare professionals, mistrust of services, and distressing medication side effects were additional barriers to continued use. Practical barriers, such as complex medication regimens, forgetfulness, and disorganisation, also contributed to medication non‐adherence, highlighting the potential value of reminder systems or structured medication support. Finally, symptom‐driven factors, including the perceived positive aspects of psychosis experiences, may increase reluctance to eliminate symptoms and thereby undermine medication use.

These findings therefore highlight that medication non‐adherence in FEP is a multifaceted phenomenon influenced by personal, relational, cognitive, cultural and systemic factors. Psychosocial interventions, such as CBTp, motivational interviewing, and culturally sensitive family‐focused approaches that address these collective influences offer the greatest potential to enhance and sustain medication adherence in FEP.

## Funding

The authors have nothing to report.

## Ethics Statement

The authors have abided by the Ethical Principles of Psychologists and Code of Conduct as set out by the BABCP and BPS.

## Conflicts of Interest

The authors declare no conflicts of interest.

## Supporting information


**Data S1:** PRISMA_2020_checklist medication paper 220126.

## Data Availability

Data sharing is not applicable to this article as no new data were created or analysed in this study.
